# Photocatalyzed C(sp^3^)–N Bond Activation of Isonitriles for Mizoroki–Heck Cross-Coupling with Vinyl Arenes

**DOI:** 10.1021/acs.orglett.5c02954

**Published:** 2025-08-26

**Authors:** Allanah B. Wood, Natsuki Mizuno, Sarah Jung, Alexander W. Schuppe

**Affiliations:** Department of Chemistry, 5718 Vanderbilt University, Nashville, Tennessee 37235, United States

## Abstract

We report a Mizoroki–Heck reaction enabled by the visible-light-induced Pd-catalyzed C–N bond cleavage of isonitriles. Through a one-carbon activation of amines, we eliminated the need for atom-inefficient activating groups, thereby allowing isonitriles to serve as alkyl radical precursors. The utility of this protocol was demonstrated with a variety of (hetero)­aromatic-containing isonitriles and differentially substituted vinyl arenes. This transformation expands the range of cross-coupling reactions that amine precursors can undergo.

## Graphical abstract

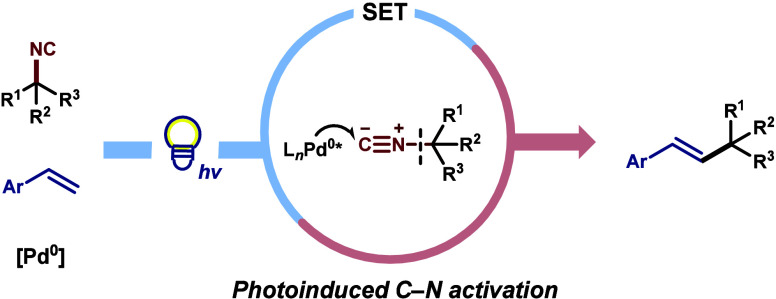

Palladium-catalyzed cross-coupling reactions have revolutionized the synthesis of complex molecules across diverse fields of chemistry.^1^ Perhaps one of the most useful of these transformations is the Mizoroki–Heck reaction, which prototypically couples aryl or vinyl (pseudo)­halide electrophiles with olefins through a facile two-electron oxidative addition (Figure 1A).^2^ Although the migratory insertion of aliphatic groups across olefins via a similar Pd-catalyzed process has attracted a significant amount of attention, the use of unactivated alkyl halides remains challenging due to the demanding nature of the oxidative addition step.^3^ Studies by Fu, Alexanian, and others demonstrated that unactivated alkyl halides could be harnessed in both inter- and intramolecular Heck couplings using aryl or alkyl olefins.^4^ However, despite the numerous developments over the decades, a barrier for the broad use of the alkyl Heck cross-coupling reaction is controlling the rate of undesired β-hydride elimination of the alkyl Pd species relative to desired migratory insertion. Gevorgyan and others have demonstrated how photoexcited Pd catalysis can overcome several limitations of this transformation and leverage a variety of unactivated coupling partners, including redox-active electrophiles.^5,6^ While photocatalyzed processes have expanded the scope of applicable substrates, the transformation of inert functional groups in the Mizoroki–Heck cross-coupling remains a synthetic challenge.

**Figure 1:**
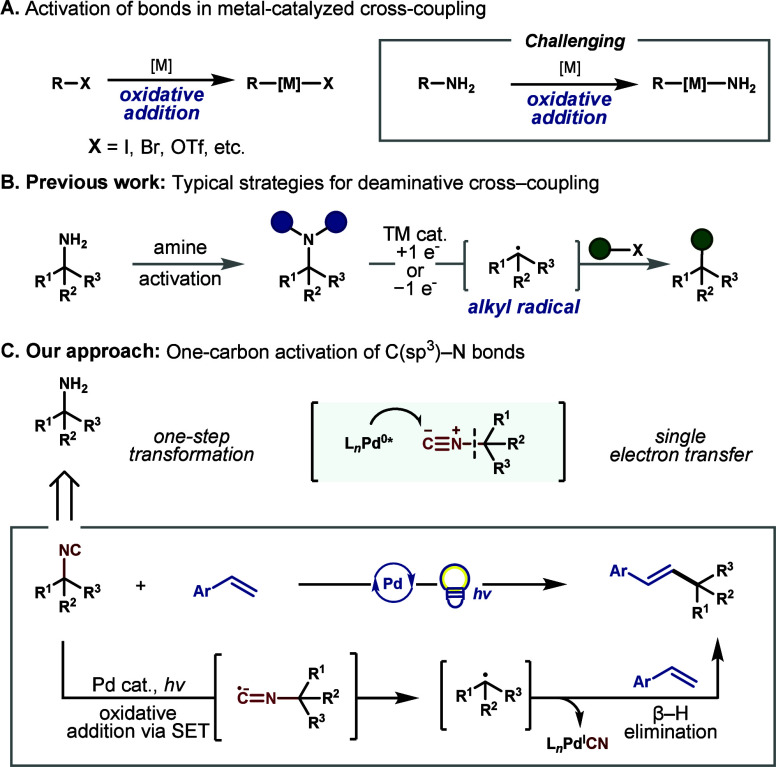
Development of C­(sp^3^)–N bond activation strategies.

Although alkyl amines are ubiquitous substructures in organic molecules, employing this functional handle in cross-coupling reactions remains limited due to the high bond dissociation energy and lack of polarization of C­(sp^3^)–N bonds.^7^ Accordingly, alkyl amines have been derivatized into redox-active auxiliaries or other intermediates (e.g., diazenes), which serve as precursors to alkyl radicals upon mesolytic or homolytic cleavage (Figure 1B).^8^ For instance, amines can be readily converted into Katritzky pyridinium salts, which can undergo deaminative functionalizations.^9^ Amines may also be transiently derivatized into redox-active imines as a complementary tactic for deaminative arylation and alkylation.^10^ Despite the utility of these approaches, one inherent drawback is the poor atom economy of the amine activating group.

To this end, our goal was to implement a one-carbon activation strategy that could enable a Mizoroki–Heck reaction upon cleavage of a relatively inert C–N bond (Figure 1C). We reasoned that isonitriles would be the ideal synthetic precursors, as the cleavage of these C­(sp^3^)–N bonds has established modes of forming alkyl radicals.^11^ Recently, our group, Tortosa, and others have reported complementary strategies for C–N bond scission via an imidoyl radical intermediate derived from isonitriles.^12^ Moreover, Chu recently disclosed a method for hydro- and alkylcyanation reactions facilitated by the C­(sp^3^)–N bond homolysis of isonitriles via a Ni-catalyzed metal-to-ligand charge transfer pathway.^13^ We envisioned that through careful tuning of the catalyst system we could develop a deaminative protocol to furnish Mizoroki–Heck products derived from isonitriles.

To accomplish this cross-coupling, we sought to activate the isonitrile C–N bond through mesolytic cleavage. The highly reducing nature of a photoexcited Pd(0) species could enable a single-electron transfer (SET) with an isonitrile, producing an alkyl radical for subsequent coupling.^6^ Although competitive deactivation and insertion of isonitriles with organo-Pd species are known phenomena, we theorized that we could avoid these deleterious pathways in the presence of an appropriate additive.^14^ Ultimately, such a method would promote inert C–N bond cleavage for the construction of new C­(sp^2^)–bonds.

We began our investigations of the C–N bond cleavage of isonitriles for a Mizoroki–Heck-type reaction using 1-isocyanoadamantane (**1a**) as our model substrate, which was prepared directly from 1-adamantanol, and styrene (**2a**). We determined that **3a** could be produced in 61% yield by employing B­(C_6_F_5_)_3_ in combination with Xantphos (**L1**), NMeCy_2_, a catalytic amount of Pd­(PPh_3_)_4_, and 427 nm LED irradiation (Table 1, entry 1). The addition of B­(C_6_F_5_)_3_ proved to be critical to the formation of the Heck coupling product, as the use of alternative Lewis acids resulted in significantly reduced or minimal yields of **3a** (entries 2–5, and see the Supporting Information). Moreover, decreasing the amount of B­(C_6_F_5_)_3_ greatly diminished the extent of formation of **3a** (entry 6). While *N*,*N*-diisopropylethylamine (DIPEA) produced **3a** in a comparable yield (entry 7), alternative organic or inorganic bases were less effective in this transformation (entries 8 and 9). Evaluation of a series of structurally similar phosphine ligands, including *t*-Bu-Xantphos (**L2**) and *N*-Xantphos (**L3**), did not yield **3a**, while *R*-MOP (**L4**) generated **3a** in 10% yield (entries 10–12). Although PhH proved to be the optimal solvent for the generation of **3a**, alternative mixtures of PhH with ethereal solvents, including 1,4-dioxane and methyl *tert*-butyl ether (MTBE), performed similarly (entries 13 and 14, respectively). However, during our investigations of alternative coupling reactions, we observed that a mixed hydrocarbon and ethereal solvent mixture often resulted in a higher yield of the Heck coupling products. We theorized that this may be due to diminishing undesired alkyl radical pathways (e.g., solvent addition) or attenuation of the Lewis acid additive. Control experiments omitting LED irradiation, a base, B­(C_6_F_5_)_3_, or Pd­(PPh_3_)_4_ did not yield **3a** (see the Supporting Information).

**Table 1:**
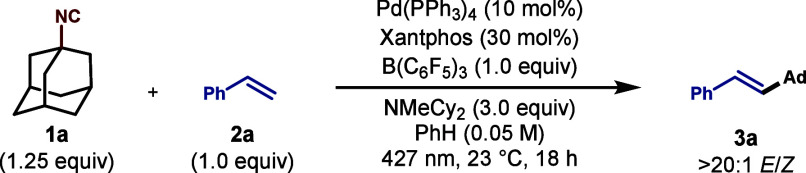
Optimization of the Heck Cross-Coupling^a^

**entry**	**variation from the standard conditions**	**yield (%)**
1	none	61
2	In(OAc)_3_	<5
3	BPh_3_	15
4	Sc(OTf)_3_	<5
5	TiCl_4_	0
6	B(C_6_F_5_)_3_ (0.5 equiv)	30
7	DIPEA	60
8	DBU	20
9	Cs_2_CO_3_	13
10	*t*-Bu*-*Xantphos (**L2**)	<5
11	*N*-Xantphos (**L3**)	<5
12	(*R*)-MOP (**L4**)	10
13	PhH/1,4-dioxane (3:1)	58
14	PhH/MTBE (3:1)	51

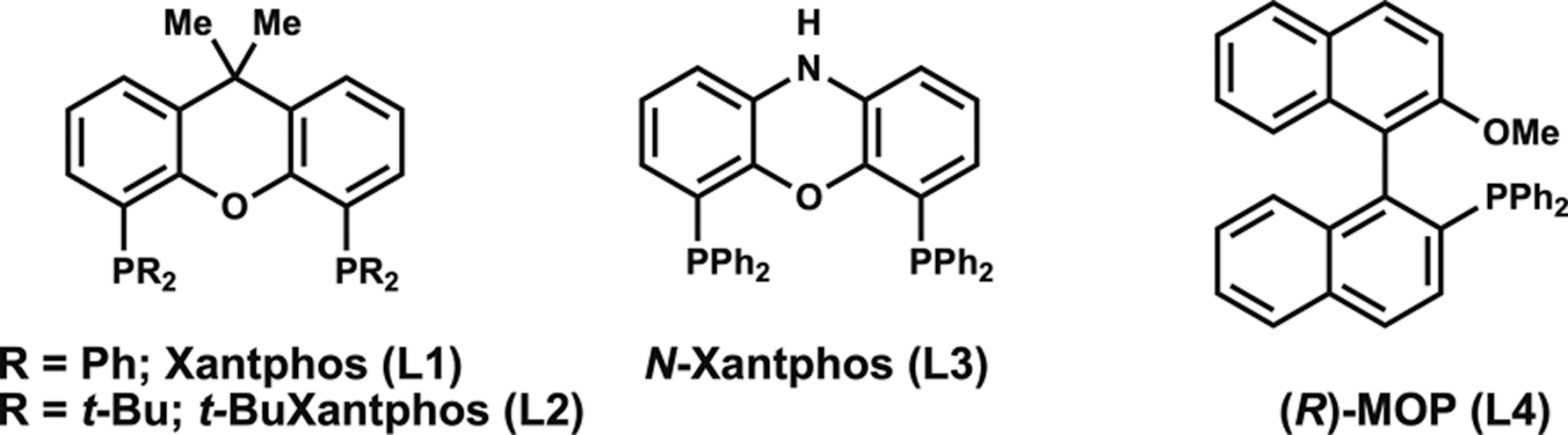

a Reaction conditions: 0.1 mmol of **2a** (1.0 equiv), **1a** (1.25 equiv), Pd­(PPh_3_)_4_ (10 mol %), Xantphos (**L1**, 30 mol %), B­(C_6_F_5_)_3_ (1.0 equiv), and NMeCy_2_ (3.0 equiv). Yields were determined by ^1^H NMR spectroscopy of the crude reaction mixtures using 1,1,2,2-tetrachloroethane (TCE) or CH_2_Br_2_ as an internal standard.

Having established the optimal reaction conditions for the photocatalyzed Heck coupling, we investigated the scope of vinyl arene coupling partners with isonitrile **1a** (Scheme 1).

**Scheme 1:**
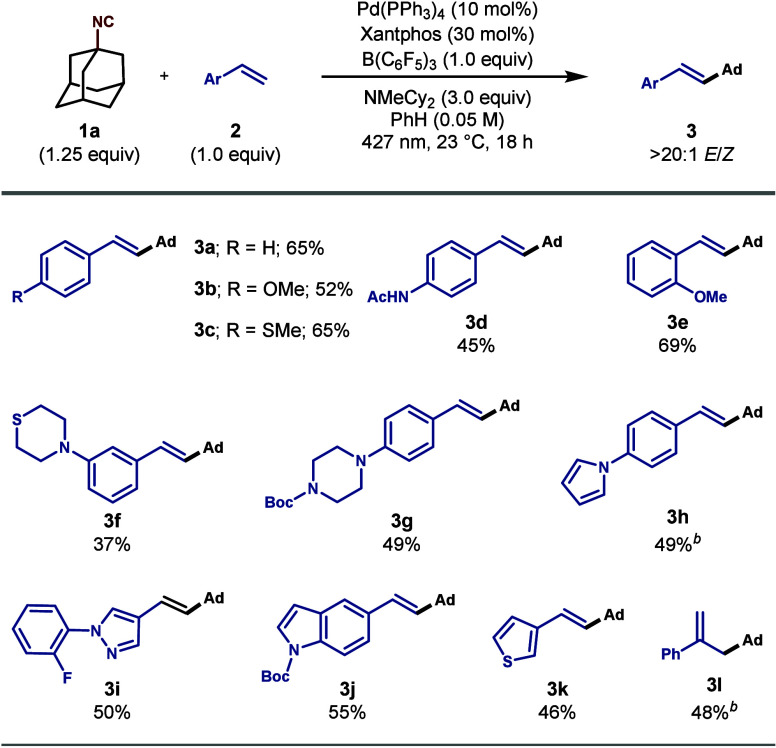
Scope of Vinyl Arenes^a^

a All reactions were conducted on a 0.3 mmol scale with respect to the vinyl arene, and yields refer to isolated yields following flash chromatography unless otherwise noted.
b The yield was determined by ^1^H NMR spectroscopy using CH_2_Br_2_ as an internal standard.

Styrene (**2a**) smoothly underwent the coupling reaction, and we found that *para*-substituted derivatives, including a thiomethyl (**3c**) and acetamide (**3d**), were compatible with our protocol. Additionally, both *para*- and *ortho*-substituted methoxy vinyl arenes could be formed under our method (**3b** and **3e**, respectively). While electron-rich and -neutral *para* substituents were well tolerated and enabled the formation of the corresponding cross-coupling adducts in good yields (**3a–3d**, **3g**, and **3h**), electron-deficient vinyl arene coupling partners (e.g., 1-(trifluoromethyl)-4-vinylbenzene) afforded negligible yields of **3**. Biologically relevant saturated heterocyclic structural elements, such as a thiomorpholine (**3f**) and a piperazine (**3g**), could be incorporated into the vinyl arene products. Additionally, various heteroaromatic-containing substructures were applicable to our protocol, including pyrrole (**3h**), pyrazole (**3i**), indole (**3j**), and thiophene (**3k**). The formation of a 1,1-disubstituted olefin was also feasible, as demonstrated by **3l**, which was isolated as a single isomer.

To further explore the scope of suitable substrates, we examined various isonitriles in combination with differentially substituted vinyl arenes (Scheme 2). We began by employing commercially available *tert*-butyl isocyanide and found that vinyl arenes containing acetamide (**3m**), pyrrole (**3n**), and benzodioxole (**3o**) substructures were all amenable to the reaction conditions. Our method efficiently produced vinyl arene products containing a pyridine (**3p**), a bicyclooctane ring (**3q**), esters (**3p**, **3q**, **3t**, and **3u**), a silyl ether (**3s**), a thiophene (**3t**), and an aryl fluoride (**3v**). Furthermore, we found that a secondary isonitrile, cyclohexyl isocyanide, successfully formed the desired product (**3r**) in good yield. To probe the scalability of our protocol, the Heck cross-coupling was performed on a 3 mmol scale and provided **3m** in comparable yield (Scheme 3).

**Scheme 2:**
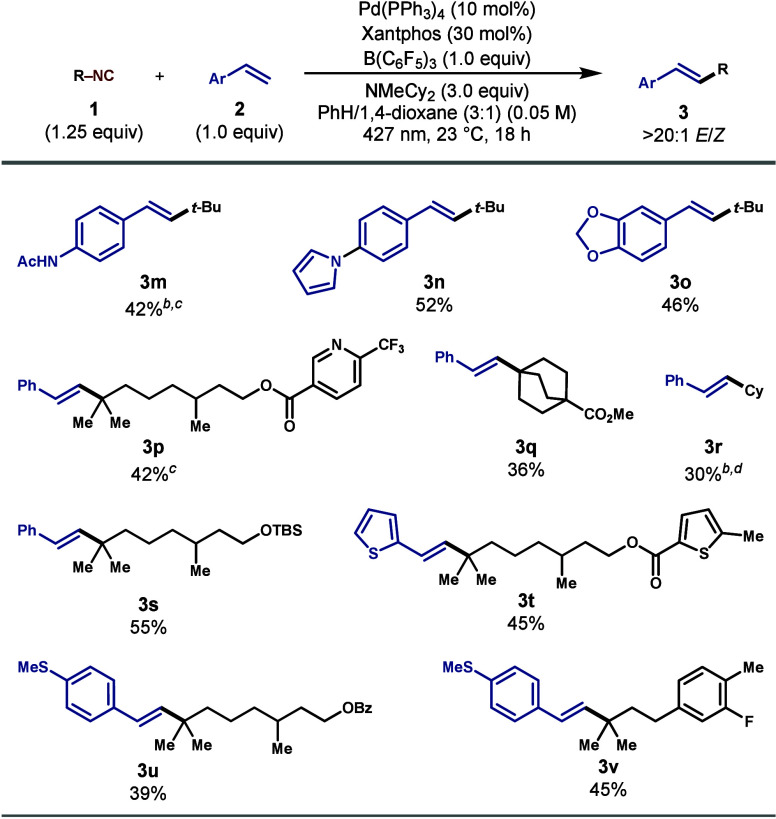
Scope of Isonitriles and Vinyl Arenes^a^

a All reactions were conducted on a 0.3 mmol scale with respect to the vinyl arene, and yields refer to isolated yields following flash chromatography unless otherwise noted.
b The yield was determined by ^1^H NMR spectroscopy using TCE or CH_2_Br_2_ as an internal standard.
c A 3:1 PhH/MTBE mixture (0.05 M) was utilized as the solvent.
d PhH (0.05 M) was utilized as the solvent.

**Scheme 3:**
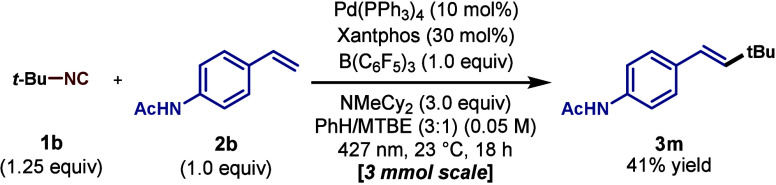
Large-Scale Heck Cross-Coupling Reaction

While speculative at this juncture, we propose a plausible catalytic cycle in Scheme 4 based on the results of our preliminary studies (see the Supporting Information). We hypothesize that isonitrile substrate **1** (ca. *E*_p,c_ = −1.38 V vs Fc^+^/Fc) is likely reduced via an outer-sphere single-electron transfer by a photoexcited Pd(0) catalyst (*E*_1/2_(Pd^+^/Pd*) = −1.72 V vs SCE in THF), generating radical anion **I**.^6c^ We observed that the addition of B­(C_6_F_5_)_3_ to **1** altered the redox potential of the isonitrile (ca. *E*_p,c_ = −1.1 V vs Fc^+^/Fc), which may therefore assist in the single-electron reduction process. Additionally, Stern–Volmer luminescence quenching studies revealed that isonitrile **1a** was capable of quenching the excited photocatalyst. Subsequent β-scission would afford alkyl radical species **II**. Radical substitution of **II** with vinyl arene **2** produces intermediate **III**, which likely rapidly furnishes **IV** upon radical recombination. Subsequent β-hydride elimination yields coupling product **3**. Reductive elimination of Pd­(II) hydride **V** is then facilitated by the base to regenerate the Pd(0) catalyst.

**Scheme 4:**
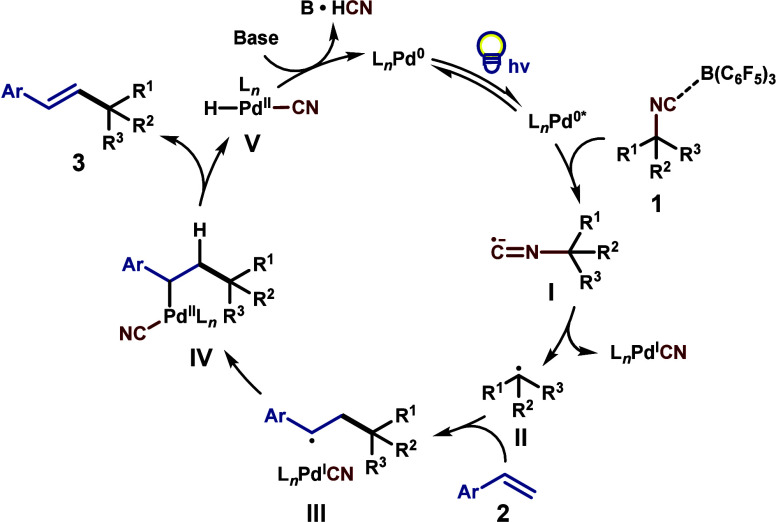
Proposed Catalytic Cycle

In summary, we have successfully developed an atom-economic C­(sp^3^)–N bond cleavage strategy that is effectively implemented in Mizoroki–Heck cross-coupling with a range of vinyl arenes. We leverage a highly reducing photoexcited Pd catalyst to readily engage isonitrile substrates. Overall, this catalytic transformation greatly diversifies the scope of possible alkyl substrates to participate in synthetically useful C–C bond-forming deaminative reactions.

## Supplementary Material



## Data Availability

The data underlying this study are available in the published article and its Supporting Information.

## Notes

Additional optimization and mechanistic experiments, full spectroscopic data for new compounds, and detailed experimental procedures (PDF)
