# Supplementary material for: Photocatalyzed C(sp3)–N Bond Activation of Isonitriles for Mizoroki–Heck Cross-Coupling with Vinyl Arenes
Source: Org Lett. 2025 Aug 26;27(35):9716–20. doi: 10.1021/acs.orglett.5c02954 (PMC12418498; doi:10.1021/acs.orglett.5c02954)
Supplement: Supplementary file 1 [file ol5c02954_si_001.pdf]

Photocatalyzed C(sp^3)–N Bond Activation of Isonitriles for Mizoroki-Heck Cross-Coupling with Vinyl Arenes

Allanah B. Wood‡, Natsuki Mizuno‡, Sarah Jung, and Alexander W. Schuppe*

Department of Chemistry, Vanderbilt University, Nashville, Tennessee, 37235, United States

Correspondence to: alexander.w.schuppe@vanderbilt.edu

Table of Contents

1. General Experimental Details **4**

2. Optimization of the Reaction Conditions **6**

2.1. **General Procedure A:** Optimization Studies 6

3. Synthesis and Characterization of Starting Materials **14**

3.1. **Synthesis and Characterization of Isonitriles and Synthetic Precursors SI-3–SI-9** 15

3.1.1. **General Procedure B :** Preparation of Substrates Through Steiglich Esterification 19

4. General Procedures for Photocatalyzed Mizoroki-Heck Cross Coupling with Vinyl Arenes **21**

4.1. **General Procedure C:** Photocatalyzed Mizoroki-Heck Cross Coupling with Vinyl Arenes 21

4.1.1. **General Graphical Procedure C:** Photocatalyzed Mizoroki-Heck Cross Coupling with Vinyl Arenes 22

4.2. **General Procedure D for Purification by Reversed-Phase Chromatography** . 24

5. Synthesis and Characterization of Mizoroki-Heck Coupling Products 3a–3v ... **25**

5.1 **General Procedure E:** Large-Scale Photocatalyzed Mizoroki-Heck Cross Coupling with Vinyl Arenes 38

5.1.1 **Graphical General Procedure E:** Large-Scale Photocatalyzed Mizoroki-Heck Cross Coupling with Vinyl Arenes 39

6. Mechanistic Experiments **40**

6.1. **Cyclic Voltammetry (CV) Experiments** 40

6.2. **Solvent Addition Byproduct** 43

6.3. **Frustrated Lewis Pairs Experiment** 46

6.4. **UV-Vis Absorption Spectroscopy** 47

6.5. **Stern-Volmer Fluorescence Quenching** 48

6.5.1. **Emission Quenching of $Pd(PPh_3)_4$ by isocyanoadamantane (1a) and $B(C_6F_5)_3$** 49

6.6. **Fluorescence Spectra** 51

6.7. **General Procedure F: Preparation of ^{31}P Experiment** 52

6.7.1. **Photoirradiated ^{31}P Experiment** 54

6.8. **^{11}B NMR/ ^{19}F NMR Studies** 55

6.9. **TEMPO Trapping Experiment** 58

7. Substrate Scope Limitations **59**

8. Associated Analytical Data **60**

8.1. **Associated NMR Spectra** 60
8.1.1. **NMR Spectra of Starting Materials** 60
8.1.2. **NMR Spectra of Products** 68
9. References 93

1. General Experimental Details

General Experimental Procedures: All reactions were performed in flame-dried glassware fitted with rubber or PTFE/silicone septa inside of a glovebox or under positive pressure of N_2, unless otherwise noted. Standard reactions were performed in glass reaction tubes with crimp-tops (Thermo Scientific, catalog no. CHCV20-14) equipped with a magnetic stir bar (Chemglass Life Sciences, catalog no. CG-2003-17, 12.7 x 3 mm) and sealed with a DWK Life Sciences PTFE/Silicone lined aluminum crimp vial seal (Thermo Scientific, catalog no. 15-111-703). Air- and moisture-sensitive liquids were transferred via syringe through rubber or PTFE/silicone septa. Solids were added under inert gas counter flow or were dissolved in the appropriate solvent. Reactions carried out at temperatures above room temperature were conducted in a pre-heated oil bath.

All reactions were magnetically stirred and monitored by ^1H NMR spectroscopy or analytical thin-layer chromatography (TLC), using glass-backed plates pre-coated with silica gel (250 μm, 60-Å pore diameter, Extra Hard Layer, SilicaPlate) impregnated with a fluorescent indicator (254 nm). TLC plates were visualized by exposure to ultraviolet light (UV) or were stained by submersion in iodine dispersed in SiO_2 (I_2), an acidic solution of *p*-anisaldehyde (PAA), an acidic solution of cerium ammonium molybdate (CAM), or an aqueous potassium permanganate solution ($KMnO_4$) and were developed by heating with a heat gun. Flash column chromatography was performed using SiliCycle SilicaFlash® or Sorbtech P60 silica gel (40–63 μm, 230–400 mesh, 60-Å pore diameter). Automated column chromatography was performed using a Biotage® Selekt Flash Chromatography System using prepacked Biotage® Sfär C18 D - Duo 100 Å 30 μm cartridges (30 g). Purification through preparative thin-layer chromatography was performed using glass-backed plates pre-coated with silica gel (1000 μm, 60-Å pore diameter) impregnated with a fluorescent indicator (254 nm). The yields refer to chromatographically and spectroscopically (^1H, ^{13}C and ^{19}F NMR) pure material. All ^1H NMR yields are corrected. For light irradiation, two Kessil PR160L-Blue LED lamps (λ_{max}= 427 nm, max 45 W) at 100% intensity were placed 3 cm away from the reaction vials in a custom-made temperature-controlled LED photoreactor setup. The reactor was cooled through the use of a 75 mm fan.

Materials: Unless noted otherwise, all reagents and starting materials were purchased from commercial sources and used as received (Millipore Sigma, TCI America, Combi-Blocks, Ambeed). $CDCl_3$ was purchased from Milipore Sigma. Benzene (PhH), tetrahydrofuran (THF), dichloromethane (CH_2Cl_2), 1,4–dioxane, methyl *tert*-butyl ether (MTBE), and methanol (MeOH) were obtained from Fisher Scientific and purified by successive filtrations though packed columns of neutral alumina or 4 Å molecular sieves under N_2 pressure. Solvents for extraction, crystallization, and flash column chromatography were purchased in ACS grade from Fisher Scientific.

Instrumentation: NMR spectra were measured on Bruker Avance III HD 400, 500, or 600 MHz spectrometers. Proton chemical shifts are expressed in parts per million (ppm, δ scale) and are referenced to the residual proton in the NMR solvent ($CDCl_3$: δ 7.26). ^1H NMR spectroscopic data are reported as follows: Chemical shift in ppm (multiplicity, coupling constants *J* (Hz), integration intensity). The multiplicities are abbreviated with s

(singlet), br s (broad singlet), d (doublet), t (triplet), at (apparent triplet), q (quartet), p (pentet), dd (doublet of doublets), qt (quartet of triplets), ddd (doublet of doublet of doublets), and m (multiplet). All ^{13}C spectra recorded are proton-decoupled. The carbon chemical shifts are expressed in parts per million (ppm, δ scale) and are referenced to the carbon resonance of the NMR solvent ($CDCl_3$: δ 77.16). ^{13}C NMR spectroscopic data are reported as follows: Chemical shift in ppm (multiplicity, coupling constants *J* (Hz)). All ^{19}F spectra were acquired without proton-decoupling. The ^{19}F chemical shifts are expressed in parts per million (ppm, δ scale). All ^{11}B spectra recorded are proton-decoupled and ^{11}B chemical shifts are expressed in parts per million (ppm, δ scale). ^{11}B and ^{19}F spectra were referenced to an external standard, $BF_3 \cdot Et_2O$ (^{11}B: δ = 0.0, ^{19}F: δ = −152.8). All ^{31}P spectra were acquired without proton-decoupling and ^{31}P chemical shifts are expressed in parts per million (ppm, δ scale). All raw ".fid" files were processed and analyzed using MestReNOVA 14.3 from Mestrelab Research S. L. High-resolution mass spectra were obtained on a LTQ Orbitrap XL™ Hybrid FT MassSpectrometer and an Agilent Technologies 6550 Q-TOF LC/MS system using an Agilent Zorbax 300 SB-C3 (2.1 × 150 mm, 5-μm particle size). Syringe pumps (BS-300) were purchased from Braintree Scientific, Inc. UV-Vis data was collected using a Hitachi U-3000 spectrophotometer. Fluorescence data was obtained using a Varian Cary Eclipse Fluorescence Spectrophotometer. FTIR spectra were obtained on a Nicolet iS5 spectrometer equipped with an iD5 diamond laminate ATR accessory from Thermo Scientific. FTIR spectra were acquired from thin-film, neat samples. If required, substances were dissolved in CH_2Cl_2 prior to direct application on the ATR unit. Data are reported as follows: frequency of absorption (cm^{-1}). Cyclic voltammograms were collected on a Pine Research WaveDriver 40 DC Bipotentiostat against an $Ag/AgNO_3$ reference electrode in MeCN and calibrated against ferrocenium (Fc^+).

2. Optimization of the Reaction Conditions

2.1. General Procedure A: Optimization Studies

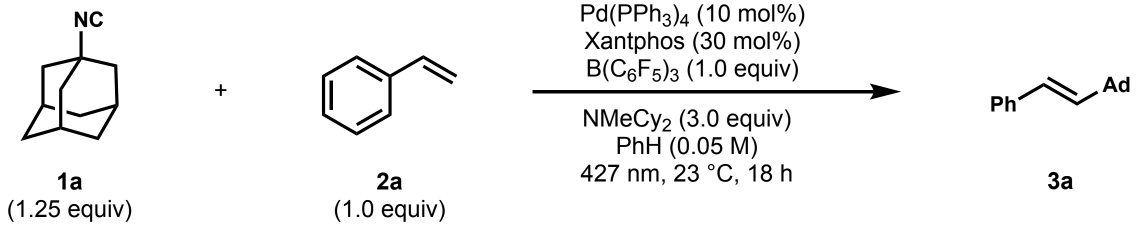

Inside a N_2-filled glovebox, to a flame-dried reaction vial (VWR, catalog no. 66011-041) equipped with a magnetic stir bar (Chemglass Life Sciences, catalog no. CG-2003-160, 10 x 3 mm) was charged 1-isocyanoadamantane (**1a**) (20.0 mg, 0.125 mmol, 1.25 equiv), $Pd(PPh_3)_4$ (12 mg, 0.010 mmol, 10 mol%), Xantphos (15 mg, 0.030 mmol, 30 mol%), and $B(C_6F_5)_3$ (51 mg, 0.10 mmol, 1.0 equiv). To the reaction mixture was added PhH (1.7 mL) and styrene (**2a**) (10 mg, 0.10 mmol, 1.0 equiv). The reaction mixture was allowed to stir. After 10 min, a solution of $NMeCy_2$ in PhH (0.30 mL, 0.30 mmol, 3.0 equiv, 1.0 M) was added dropwise down the walls of the reaction vessel to the mixture over *ca.* 5 min.

The reaction vessel was sealed with a PTFE-lined phenolic vial screw cap (Thermo Scientific, catalog no. 03-375-25A with 03-340-10G), removed from the glovebox, and placed in a custom-made photoreactor 3 cm away from two 45 W Kessil PR-160L 427 nm LEDs and one 75 mm fan. The reaction mixture was subjected to LED irradiation at 100% intensity with vigorous stirring. After 18h, the LEDs were turned off and the reaction mixture was diluted with sat. aq. $NaHCO_3$ (5 mL) and allowed to stir. After 10 min, the reaction mixture was transferred to a 16 x 150 mm test tube. The layers were separated and the aqueous layer was extracted with Et_2O (3 x 5 mL). The combined organic layers were washed with brine (5 mL), dried over Na_2SO_4, filtered, and concentrated *in vacuo* with the aid of a rotary evaporator. Yield was evaluated by ^1H NMR of the crude reaction mixture using 1,1,2,2-tetrachloroethane (TCE) (*ca.* 8.4 mg) or CH_2Br_2 (*ca.* 8.7 mg) as the internal standard.

Table 2.1. General Optimization Experiments and Control Reactions

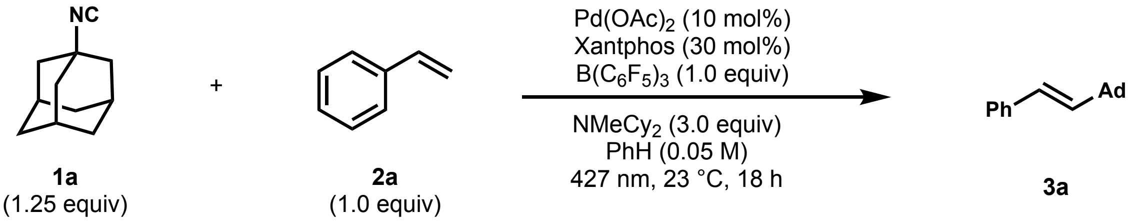

entry	variation from standard conditions	yield[a]
**1**	$PhCF_3$	27%
**2**	PhCl	35%
**3**	PhH/1,4-dioxane (5:1)	30%
**4**	370 nm	29%
**5**	390 nm	47%
**6**	440 nm	47%
**7**	Blue light	7%
**8**	No light	0%
**9**	No $NMeCy_2$	0%
**10**	No $Pd(OAc)_2$	0%

[a]All reactions performed on 0.05 mmol scale with respect to **2a**. Yields determined by ^{1}H NMR spectroscopy of the crude reaction mixtures utilizing 1,1,2,2-tetrachloroethane (TCE) as the internal standard.

Table 2.2. Base Screen

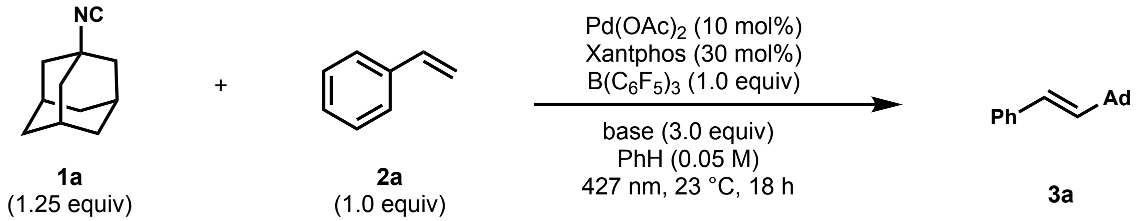

entry	variation from standard conditions	yield[a]
1	DBU	31%
2	TMEDA	30%
3	DABCO	18%
4	quinuclidine	8%
5	*N,N*-diisopropylethylamine	19%
6	2,6-lutidine	3%
7	2-*tert*-butyl-1,1,3,3-tetramethylguanidine	47%
8	Cs_2CO_3	13%

[a]All reactions performed on 0.05 mmol scale with respect to **2a**. Yields determined by ^{1}H NMR spectroscopy of the crude reaction mixtures utilizing 1,1,2,2-tetrachloroethane (TCE) as the internal standard.

Table 2.3. Additional Base Screen

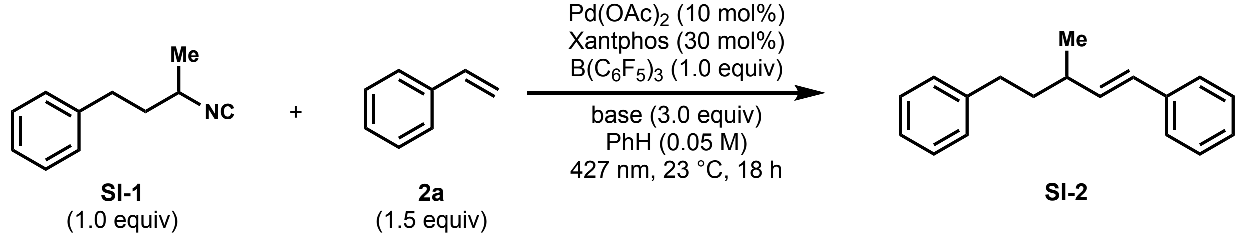

entry	variation from standard conditions	yield[a]
1	Proton-sponge	0%
2	$NMeCy_2$	26%
3	$NMeCy_2$ (4.5 equiv)	11%
4	*N*-methylpiperidine	17%
5	1,2,2,6,6-pentamethylpiperidine	23%
6	Et_3N	17%
7	$NBnMe_2$	15%
8	1-benzyl-1,4-dihydronicotinamide	0%
9	tetramethylethylenediamine	10%
10	*N*,*N*-diisopropylethylamine	22%
11	Cs_2CO_3	9%
12	K_2CO_3	5%
13	collidine	6%

[a]All reactions performed on 0.05 mmol scale with respect to **SI-1**. Yields determined by ^{1}H NMR spectroscopy of the crude reaction mixtures utilizing 1,1,2,2-tetrachloroethane (TCE) as the internal standard.

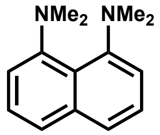

Proton-sponge

Table 2.4. Lewis Acid Equivalents Screen

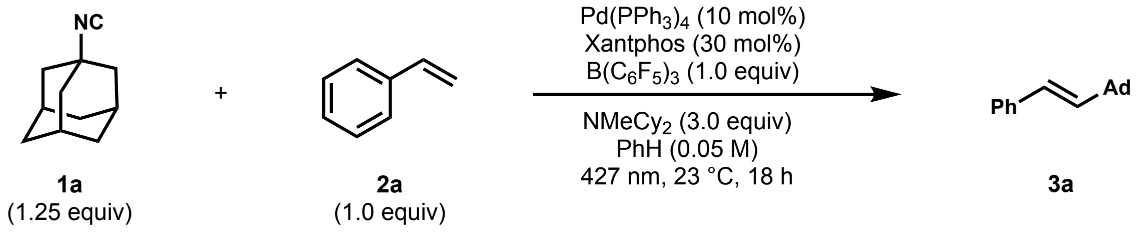

entry	variation from standard conditions	Run 1 yield[a]	Run 2 yield[a]	Average yield[a]
**1**	none	57%	54%	56%
**2**	0 equiv $B(C_6F_5)_3$	–	trace	–
**3**	0.25 equiv $B(C_6F_5)_3$	30%	15%	23%
**4**	0.50 equiv $B(C_6F_5)_3$	18%	20%	19%
**5**	0.75 equiv $B(C_6F_5)_3$	8%	42%	25%
**6**	1.25 equiv $B(C_6F_5)_3$	19%	49%	34%

[a]All reactions performed on 0.05 mmol scale with respect to **2a**. Yields determined by ^{1}H NMR spectroscopy of the crude reaction mixtures utilizing 1,1,2,2-tetrachloroethane (TCE) as the internal standard.

Table 2.5. Additional Lewis Acid Screen

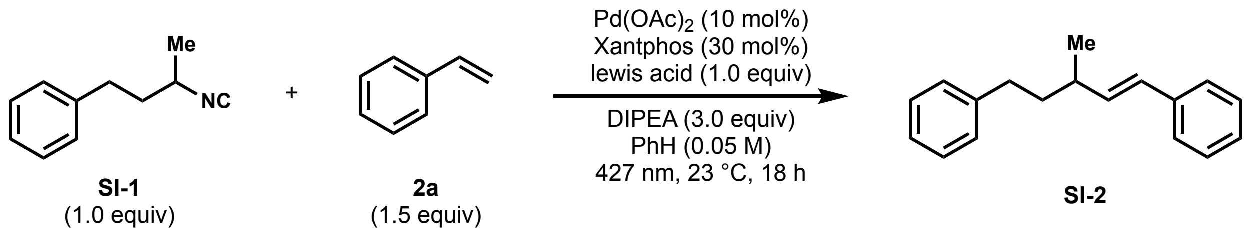

entry	variation from standard conditions	yield[a]
**1**	$ZnCl_2$	<5%
**2**	$BF_3 \cdot Et_2O$	<5%
**3**	LiCl	0%[b]
**4**	$In(acac)_3$	<5%
**5**	$Sc(OTf)_3$	0%
**6**	$Ti(O\textit{i}\text{-}Pr)_4$	0%
**7**	$FeCl_3$	0%
**8**	$B(C_6F_5)_3$ (0.5 equiv)	10%
**9**	$B(C_6F_5)_3$ (0.75 equiv)	28%
**10**	$B(C_6F_5)_3$ (0.9 equiv)	21%
**11**	$B(C_6F_5)_3$ (1.25 equiv)	23%
**12**	$B(C_6F_5)_3$ (1.5 equiv)	6%
**13**	$B(C_6F_5)_3$ (3.0 equiv)	0%

[a]All reactions performed on 0.05 mmol scale with respect to **SI-1**. Yields determined by ^{1}H NMR spectroscopy of the crude reaction mixtures utilizing 1,1,2,2-tetrachloroethane (TCE) as the internal standard.
[b]Reaction performed with THF as the solvent.

Table 2.6. Pd Precatalyst Screen

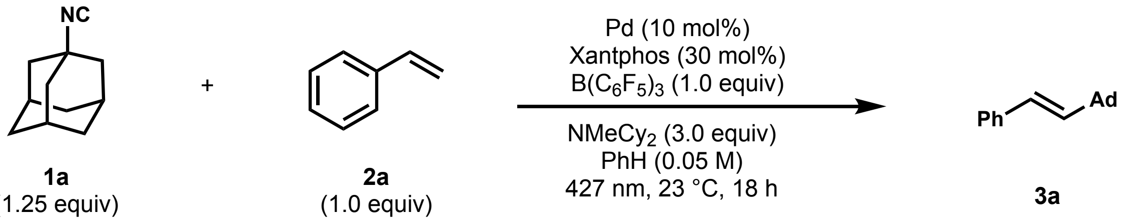

entry	variation from standard conditions	yield[a]
**1**	PdG3 *μ*-OMs dimer	32%
**2**	$[Pd(allyl)Cl]_2$	18%
**3**	$PdCl_2$	35%
**4**	$Pd(acac)_2$	45%
**5**	$Pd(dba)_2$	44%
**6**	Pd(*t*-$Bu_3P)_2$	35%

[a]All reactions performed on 0.05 mmol scale with respect to **2a**. Yields determined by ^{1}H NMR spectroscopy of the crude reaction mixtures utilizing 1,1,2,2-tetrachloroethane (TCE) as the internal standard.

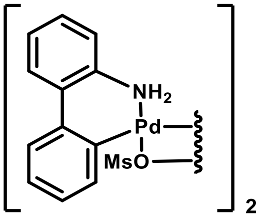

PdG3 *μ*-OMs dimer

Table 2.7. Additional Pd Precatalyst Screen

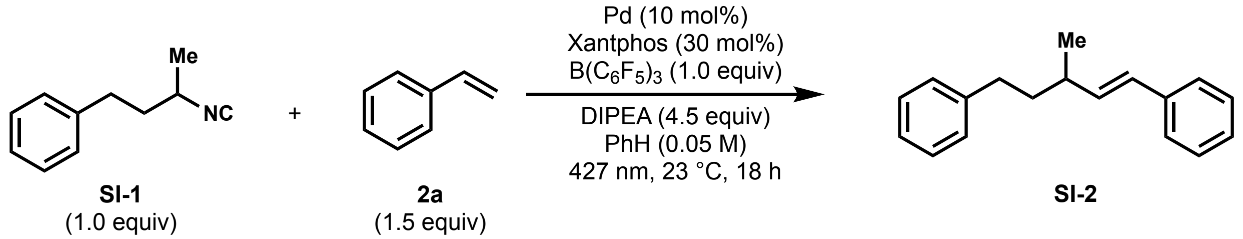

entry	variation from standard conditions	yield[a]
**1**	$Pd(OAc)_2$ (10 mol%)	28%
**2**	$Pd(OAc)_2$ (20 mol%)	15%
**3**	$Pd(OAc)_2$ (30 mol%)	9%
**4**	PdG3 *μ*-OMs dimer	27%
**5**	$[Pd(allyl)Cl]_2$	31%
**6**	$Pd(acac)_2$	12%
**7**	$Pd(dba)_2$	10%
**8**	$PdCl_2$	13%

[a]All reactions performed on 0.05 mmol scale with respect to **SI-1**. Yields determined by ^{1}H NMR spectroscopy of the crude reaction mixtures utilizing 1,1,2,2-tetrachloroethane (TCE) as the internal standard.

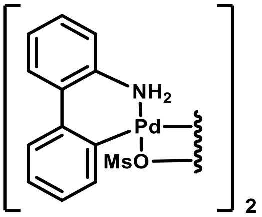

PdG3 *μ*-OMs dimer

3. Synthesis and Characterization of Starting Materials

The following starting materials used in this study were purchased or prepared according to the listed reference:[1–10]

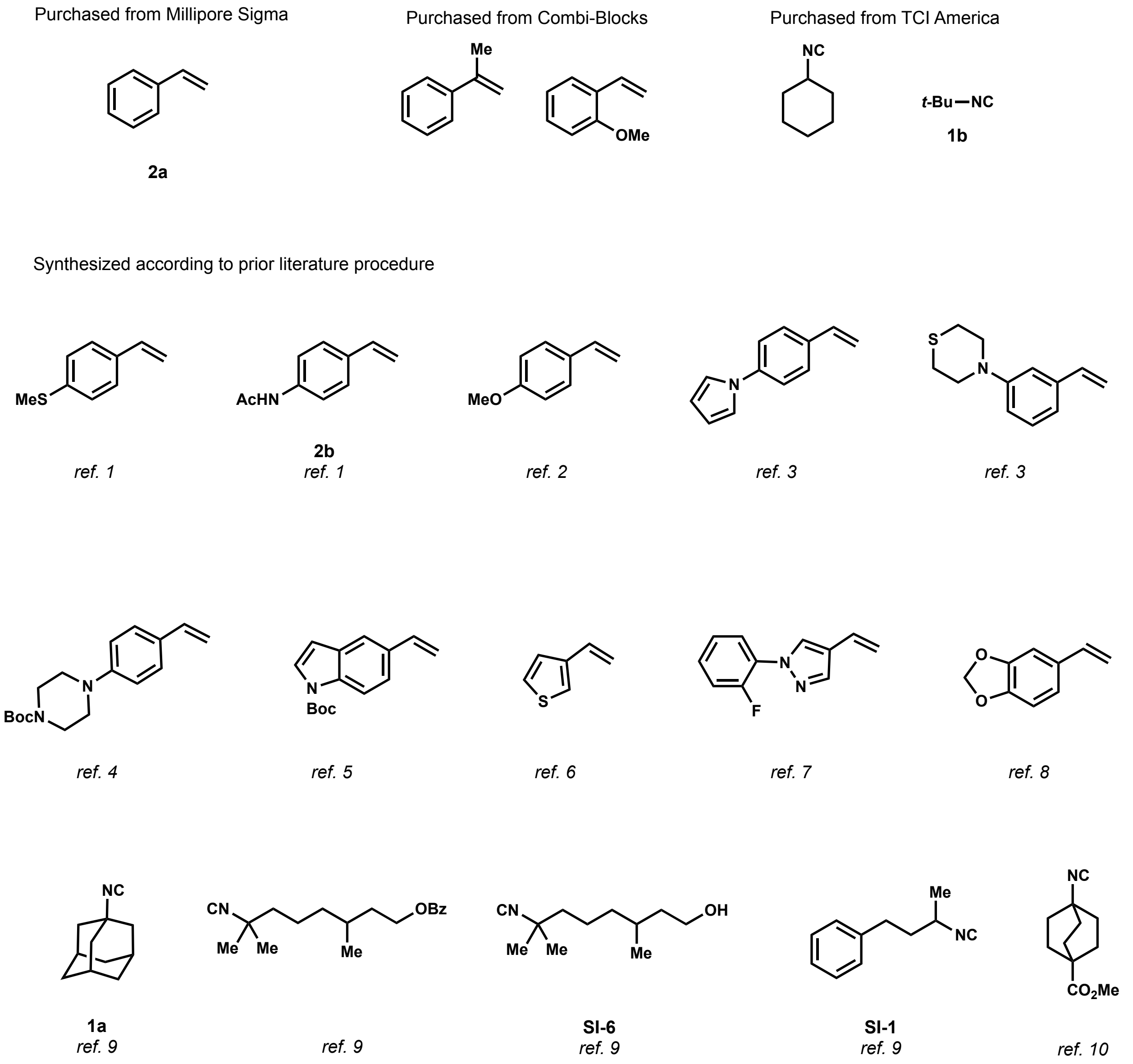

Figure SI-1: Commercial and previously synthesized starting materials.

3.1. Synthesis and Characterization of Isonitriles and Synthetic Precursors SI-3–SI-9

4-(3-Fluoro-4-methylphenyl)-2-methylbut-3-yn-2-amine (SI-3)

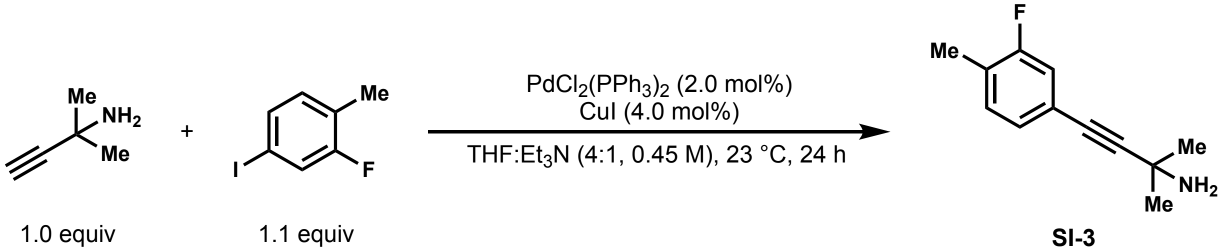

SI-3

To a flame-dried 50 mL round-bottom flask equipped with a magnetic stir bar was added bis(triphenylphosphine)palladium chloride (110 mg, 0.16 mmol, 2.0 mol%) and cuprous iodide (61 mg, 0.32 mmol, 4.0 mol%). The reaction flask was fitted with a rubber septum and connected to a dual manifold Schlenk line. The flask was then evacuated/backfilled with N_2 (this process was repeated a total of three times). The reaction mixture was then diluted with degassed THF and Et_3N (18 mL, 0.45 M, 4:1). Next, to the reaction mixture was added 2-fluoro-4-iodo-1-methylbenzene (1.2 mL, 8.8 mmol, 1.1 equiv) and 2-methylbut-3-yn-2-amine (0.84 mL, 8.0 mmol, 1.0 equiv). The reaction mixture was allowed to stir at room temperature.

After 24 h, the septum was removed from the reaction vessel and the reaction mixture was diluted with sat. aq. NH_4Cl (10 mL) and transferred to a separatory funnel. The layers were separated, and the aqueous layer was extracted with Et_2O (3 x 5 mL). The combined organic layers were washed with brine (10 mL), dried over Na_2SO_4, and concentrated *in vacuo* with the aid of a rotary evaporator. The residue was purified by flash column chromatography on silica gel (gradient elution: 30% Et_2O/2% Et_3N in hexanes to 50% Et_2O/2% Et_3N in hexanes) to afford 4-(3-fluoro-4-methylphenyl)-2-methylbut-3-yn-2-amine (**SI-3**) as an orange oil (977 mg, 64%).

^{1}H NMR (600 MHz, $CDCl_3$): δ 7.10–7.04 (m, 2H), 7.02 (dd, *J* = 10.3, 1.4 Hz, 1H), 2.25 (d, *J* = 1.7 Hz, 3H), 1.67 (br s, 2H), 1.48 (s, 6H).

^{13}C NMR (151 MHz, $CDCl_3$): δ 160.9 (d, *J* = 244.7 Hz), 131.3 (d, *J* = 5.9 Hz), 127.2 (d, *J* = 3.3 Hz), 125.2 (d, *J* = 17.2 Hz), 122.5 (d, *J* = 9.4 Hz), 118.0 (d, *J* = 23.7 Hz), 97.2, 79.2 (d, *J* = 3.0 Hz), 45.8, 31.9, 14.6 (d, *J* = 3.3 Hz).

^{19}F NMR (471 MHz, $CDCl_3$): δ −117.6.

IR (Diamond-ATR, neat) ṽ (cm^{-1}): 3358, 2974, 2359,1568,1506, 1214,1192, 1116, 869.

HRMS (ESI): m/z: $[M+H]^+$ calc'd for $C_{12}H_{15}FN^+$: 192.1183. Found: 192.1181.

4-(3-Fluoro-4-methylphenyl)-2-methylbutan-2-amine (SI-4)

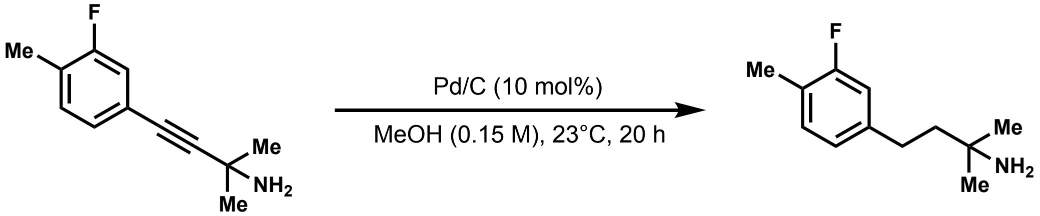

SI-3 SI-4

To a flame-dried 100 mL round-bottom flask equipped with a magnetic stir bar was added Pd/C (540 mg, 10% wt, 0.51 mmol, 10 mol%). The reaction flask was fitted with a rubber septum and connected to a dual manifold Schlenk line. The flask was then evacuated/backfilled with N_2 (this process was repeated a total of three times). To the reaction mixture was added a solution of **SI-3** (0.98 g, 5.1 mmol, 1.0 equiv) in MeOH (final concentration: 0.15 M). The reaction mixture was further diluted with MeOH (34 mL, 0.15 M). While the reaction mixture was stirring, the reaction mixture was sparged with N_2 (*ca*. 10 min) to remove air from the reaction mixture.

Following this, the flask was then carefully backfilled with H_2 using a needle connected to a H_2-filled balloon. The reaction mixture was then sparged with H_2 for 10 min before the H_2-filled balloon was then removed from the reaction solvent and placed in the headspace of the reaction vessel. After 20 h, the mixture was filtered through celite and concentrated *in vacuo* with the aid of a rotary evaporator as a colorless oil. The crude product (**SI-4**) was utilized without further purification.

2-Fluoro-4-(3-isocyano-3-methylbutyl)-1-methylbenzene (SI-5)

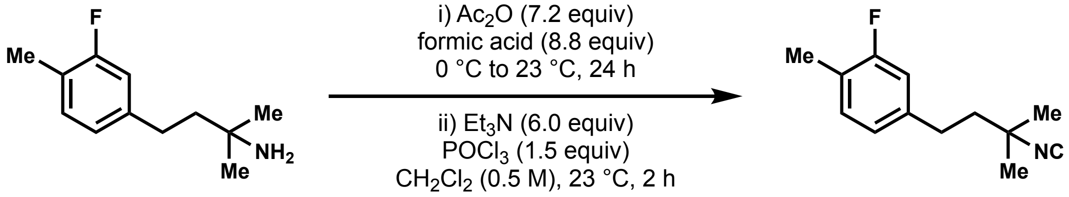

SI-4 SI-5

A flame-dried 10 mL microwave vial equipped with a magnetic stir bar was sealed with a crimp cap and connected to a dual manifold Schlenk line. The vial was then evacuated/backfilled with N_2 (this process was repeated a total of three times). To the vial was added acetic anhydride (3.5 mL, 37 mmol, 7.2 equiv) and formic acid (1.7 mL, 45 mmol, 8.8 equiv). The vial was then placed in an oil bath pre-heated to 55 °C and the reaction mixture was allowed to stir for 2 h.

A flame-dried 50 mL round-bottom flask equipped with a magnetic stir bar was charged with the crude product (**SI-4**) and fitted with a rubber septum. The flask was connected to a dual manifold Schlenk line then evacuated/backfilled with N_2 (this process was repeated a total of three times). Then the reaction vessel was placed in a 0 °C ice-water bath. After 2 h, the acetic formic anhydride solution was added dropwise via syringe

to **SI-4** and subsequently sonicated until the complete dissolution of **SI-4** was observed. The reaction mixture was warmed up to room temperature and allowed to stir. After 24 h, the mixture was concentrated *in vacuo* with the aid of a rotary evaporator.

The reaction vessel was then fitted with a rubber septum and connected to a dual manifold Schlenk line. The flask was then evacuated/backfilled with N_2 (this process was repeated a total of three times). The resulting residue was diluted with CH_2Cl_2 (10 mL, 0.5 M) and the reaction vessel was placed in a 0 °C ice-water bath. Then Et_3N (4.3 mL, 31 mmol, 6.0 equiv) was added, and the reaction mixture was allowed to stir for 5 min before phosphorus oxychloride (0.71 mL, 7.7 mmol, 1.5 equiv) was added dropwise via syringe over 1 min. The reaction was allowed to stir in a 0 °C ice-water bath. After 2 h, the crimp cap was removed from the reaction vessel and the reaction mixture was diluted with sat. aq. $NaHCO_3$ (5 mL) and transferred to a separatory funnel. The layers were separated, and the aqueous layer was extracted with CH_2Cl_2 (3 x 5 mL). The combined organic layers were washed with brine (10 mL), dried over Na_2SO_4, and concentrated *in vacuo* with the aid of a rotary evaporator. The residue was purified by flash column chromatography on silica gel (gradient elution: 2.5% EtOAc in hexanes to 5% EtOAc in hexanes) to afford 2-fluoro-4-(3-isocyano-3-methylbutyl)-1-methylbenzene (**SI-5**) as a colorless solid (740 mg, 71%).

^{1}H NMR (600 MHz, $CDCl_3$): δ 7.10 (t, *J* = 7.9 Hz, 1H), 6.89–6.83 (m, 2H), 2.78–2.72 (m, 2H), 2.24 (d, *J* = 1.7 Hz, 3H), 1.88–1.81 (m, 2H), 1.49–1.44 (m, 6H).

^{13}C NMR (151 MHz, $CDCl_3$): δ 161.4 (d, *J* = 244.8 Hz), 153.9 (t, *J* = 4.5 Hz), 140.6 (d, *J* = 7.3 Hz), 131.6 (d, *J* = 5.6 Hz), 123.8 (d, *J* = 3.3 Hz), 122.5 (d, *J* = 17.1 Hz), 114.9 (d, *J* = 22.1 Hz), 57.3 (t, *J* = 5.0 Hz), 44.2, 30.3, 29.1, 14.3 (d, *J* = 3.4 Hz).

^{19}F NMR (471 MHz, $CDCl_3$): δ −117.7.

IR (Diamond-ATR, neat) ṽ (cm^{-1}): 2984, 2359, 2131, 1512, 1422, 1253,1114, 819.

HRMS (ESI): m/z: $[M+H]^+$ calc'd for $C_{13}H_{17}FN^+$: 206.1340. Found: 206.1342.

***tert*-Butyl((7-isocyano-3,7-dimethyloctyl)oxy)dimethylsilane (SI-7)**

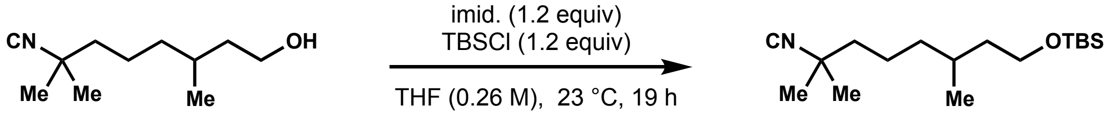

SI-6 **SI-7**

To a flame-dried 25 mL round-bottom flask equipped with a magnetic stir bar was added imidazole (220 mg, 3.3 mmol, 1.2 equiv). The reaction flask was then fitted with a rubber septum and connected to a dual manifold Schlenk line. The flask was then evacuated/backfilled with N_2 (this process was repeated a total of three times). The reaction was diluted with THF (11 mL, 0.26 M). To the reaction mixture was 7-isocyano-3,7-dimethyloctan-1-ol (**SI-6**) (0.5 g, 3.0 mmol, 1.0 equiv) and allowed to stir. After 10 min,

tert-butyldimethylsilyl chloride (490 mg, 3.3 mmol, 1.2 equiv) was added in one portion and allowed to stir at room temperature.

After 19 h, the reaction mixture was diluted with sat. aq. $NaHCO_3$ (5 mL) and Et_2O (5 mL) and the reaction was transferred to a separatory funnel. The layers were separated and the aqueous layer was extracted Et_2O (3 x 5 mL). The combined organic layers were washed with brine (5 mL), dried over Na_2SO_4 and concentrated *in vacuo* with the aid of a rotary evaporator. The residue was purified by flash column chromatography on silica gel (gradient elution: hexanes to 10 % Et_2O in hexanes) to afford *tert*-butyl((7-isocyano-3,7-dimethyloctyl)oxy)dimethylsilane (**SI-7**) as a colorless oil (670 mg, 83%).

^{1}H NMR (600 MHz, $CDCl_3$): δ 3.69–3.56 (m, 2H), 1.62–1.41 (m, 6H), 1.40 (dd, $J_{1,2}$ = $J_{1,3}$ = 2.0 Hz, 6H), 1.36–1.28 (m, 2H), 1.19–1.11 (m, 1H), 0.91–0.84 (m, 12H), 0.05 (s, 6H).

^{13}C NMR (151 MHz, $CDCl_3$): δ 153.0 (t, *J* = 4.7 Hz), 61.4, 57.5 (t, *J* = 4.8 Hz), 42.8, 40.0, 37.0, 29.4, 29.1, 26.1, 21.7, 19.7, 18.5, −5.1, −5.1.

IR (Diamond-ATR, neat) ṽ (cm^{-1}): 2931, 2359, 2337, 1252, 1089, 832, 772.

HRMS (ESI): m/z: $[M+H]^+$ calc'd for $C_{17}H_{36}NOSi^+$: 298.2561. Found: 298.2560.

3.1.1. General Procedure B: Preparation of Starting Materials Through Steiglich Esterification

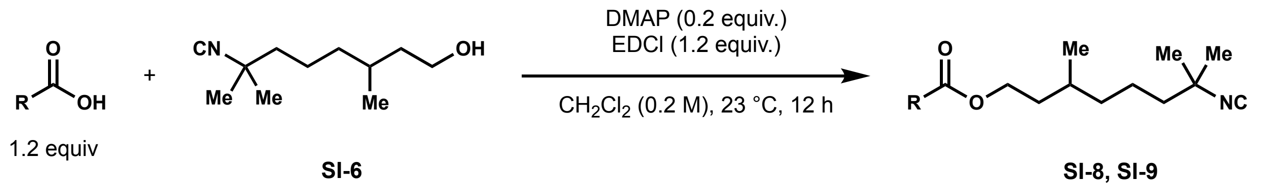

To a flame-dried 25 mL round-bottom flask equipped with a magnetic stir bar was added the corresponding carboxylic acid (1.2 equiv), 4-dimethylaminopyridine (0.2 equiv), and 1-(3-dimethylaminopropyl)-3-ethylcarbodiimide hydrochloride (1.2 equiv). The flask was then fitted with a rubber septum and connected to a dual manifold Schlenk line. The flask was then evacuated/backfilled with N_2 (this process was repeated a total of three times). After the reaction mixture was diluted with CH_2Cl_2 (0.2 M), the rubber septum was removed, and to the stirred reaction mixture was added 7-isocyano-3,7-dimethyloctan-1-ol (**SI-6**) (1.0 equiv) at room temperature, and the flask was resealed with the rubber septum and allowed to stir at room temperature.

After 12 h, the septum was removed and the reaction mixture was diluted with H_2O (5 mL). The layers were separated and the aqueous layer was extracted with CH_2Cl_2 (3 x 5 mL). The combined organic layers were washed with brine (10 mL), dried over Na_2SO_4, filtered, and concentrated *in vacuo* with the aid of a rotary evaporator. The crude mixture was then purified by flash column chromatography on silica gel to obtain the corresponding isonitrile product (**SI-8**, **SI-9**).

7-Isocyano-3,7-dimethyloctyl 6-(trifluoromethyl)nicotinate (SI-8)

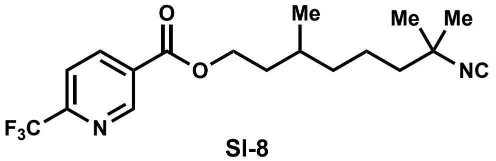

SI-8

Prepared according to **General Procedure B** using 6-(trifluoromethyl)pyridine-3-carboxylic acid (0.50 g, 2.6 mmol, 1.2 equiv) and 7-isocyano-3,7-dimethyloctan-1-ol (**SI-6**) (0.40 g, 2.2 mmol, 1.0 equiv). The residue was purified by flash column chromatography on silica gel (gradient elution: hexanes to 20% EtOAc in hexanes) to afford 7-isocyano-3,7-dimethyloctyl 6-(trifluoromethyl)nicotinate (**SI-8**) as a colorless oil (640 mg, 82%).

^{1}H NMR (600 MHz, $CDCl_3$): δ 9.30 (d, *J* = 1.5 Hz, 1H), 8.48 (dd, *J* = 8.1, 1.5 Hz, 1H), 7.79 (d, *J* = 8.1 Hz, 1H), 4.48–4.40 (m, 2H), 1.89–1.81 (m, 1H), 1.70–1.58 (m, 2H), 1.57–1.49 (m, 3H), 1.49–1.44 (m, 1H), 1.43–1.36 (m, 7H), 1.29–1.21 (m, 1H), 1.00 (d, *J* = 6.5 Hz, 3H).

^{13}C NMR (151 MHz, $CDCl_3$): δ 164.2, 153.2 (t, *J* = 4.5 Hz), 151.4 (q, *J* = 35.2 Hz), 151.1, 138.9, 128.9, 121.2 (q, *J* = 274.4 Hz), 120.4 (q, *J* = 2.6 Hz), 64.6, 57.5 (t, *J* = 4.9 Hz), 42.7, 36.8, 35.5, 30.1, 29.2, 29.1, 21.7, 19.5.

^{19}F NMR (471 MHz, $CDCl_3$): δ −68.3.

IR (Diamond-ATR, neat) ṽ (cm^{-1}): 2945, 2131, 1726, 1332, 1282,1119, 1084, 1023.

HRMS (ESI): m/z: $[M+H]^+$ calc'd for $C_{18}H_{24}F_3N_2O_2^+$: 357.1784. Found: 357.1784.

7-Isocyano-3,7-dimethyloctyl 5-methylthiophene-2-carboxylate (SI-9)

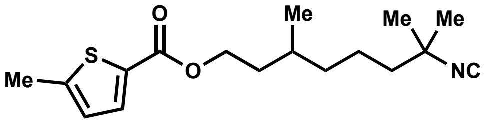

SI-9

Prepared according to **General Procedure B** using 5-methylthiophene-2-carboxylic acid (0.34 g, 2.4 mmol, 1.2 equiv) and 7-isocyano-3,7-dimethyloctan-1-ol (**SI-6**) (0.37 g, 2.0 mmol, 1.0 equiv). The residue was purified by flash column chromatography on silica gel (gradient elution: hexanes to 10% EtOAc in hexanes) to afford 7-isocyano-3,7-dimethyloctyl 5-methylthiophene-2-carboxylate (**SI-9**) as a colorless oil (480 mg, 79%).

^{1}H NMR (600 MHz, $CDCl_3$): δ 7.60 (d, *J* = 3.7 Hz, 1H), 6.76 (dd, *J* = 3.7, 0.9 Hz, 1H), 4.36–4.24 (m, 2H), 2.52 (d, *J* = 0.9 Hz, 3H), 1.82–1.74 (m, 1H), 1.69–1.60 (m, 1H), 1.59–1.41 (m, 6H), 1.41–1.38 (m, 6H), 1.27–1.17 (m, 1H), 0.97 (d, *J* = 6.6 Hz, 3H).

^{13}C NMR (151 MHz, $CDCl_3$): δ 162.4, 153.0 (t, *J* = 4.6 Hz),147.9, 133.8, 131.4, 126.4, 63.4, 57.5 (t, *J* = 4.9 Hz), 42.7, 36.8, 35.6, 29.9, 29.1, 29.1, 21.6, 19.6, 15.9.

IR (Diamond-ATR, neat) ṽ (cm^{-1}): 2928, 2359, 2130, 1470, 1254, 1093, 834, 774.

HRMS (ESI): m/z: $[M+H]^+$ calc'd for. $C_{17}H_{26}NO_2S^+$: 308.1679. Found: 308.1679.

4. General Procedures for Photocatalyzed Mizoroki-Heck Cross Coupling with Vinyl Arenes

4.1. General Procedure C: Photocatalyzed Mizoroki-Heck Cross Coupling with Vinyl Arenes

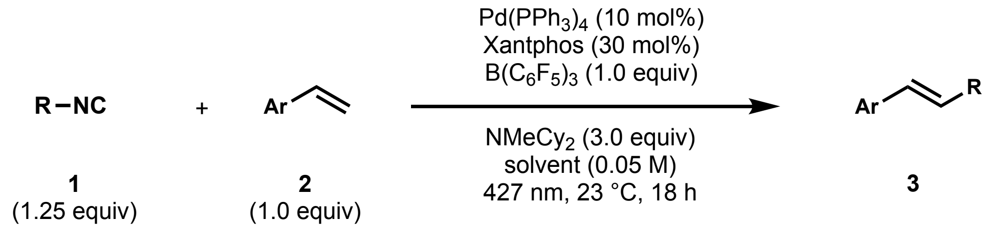

Inside a N_2-filled glovebox, a flame-dried reaction tube (Thermo Scientific, catalog no. CHCV20-14) equipped with a magnetic stir bar, labelled as "**A**", was charged with $Pd(PPh_3)_4$ (35 mg, 0.03 mmol, 10 mol%), Xantphos (52 mg, 0.09 mmol, 30 mol%), and $B(C_6F_5)_3$ (154 mg, 0.30 mmol, 1.0 equiv). If solid, the corresponding isonitrile (**1**) (0.375 mmol, 1.25 equiv, Figure **SI-2b**), and/or vinyl arene (**2**) (0.30 mmol, 1.0 equiv, Figure **SI-2b**) were added at this point. Reaction tube "**A**" was then fitted with a rubber septum. If the reaction condition required a mixed solvent, in a flame-dried 20 mL microwave vial equipped with a magnetic stir bar, labeled as "**B**", a solvent solution of either PhH/1,4–dioxane (3:1) or PhH/MTBE (3:1) was prepared (Figure **SI-2b**).

Then, the reaction tube "**A**" was diluted in either PhH (5.1 mL) or the solvent from tube "**B**" (5.1 mL). If liquid, the corresponding isonitrile (**1**) (0.375 mmol, 1.25 equiv), and/or vinyl arene (**2**) (0.30 mmol, 1.0 equiv, Figure **SI-2b**, Figure **SI-2c**) were added to reaction tube "**A** The reaction mixture was allowed to stir for 10 min (Figure **SI-2c**). During this period, a flame-dried reaction tube (Thermo Scientific, catalog no. CHCV20-14) equipped with a magnetic stir bar, labelled as "**C**", was charged with $NMeCy_2$ (195 mg, 1.0 mmol, 3.3 equiv) and diluted with PhH or the corresponding solvent from tube "**B**" (1.0 mL, 1.0 M), and allowed to stir to prepare a stock solution.

After the reaction mixture had stirred for 10 min, the stock solution of $NMeCy_2$ from reaction tube "**C**" (0.9 mL, 0.9 mmol, 3.0 equiv, 1.0 M) was added dropwise down the walls of reaction tube "**A**" over *ca.* 10 min (Figure **SI-2c**). The rubber septum was then removed from reaction tube "**A**" and then sealed with a PTFE/silicone lined aluminum crimp vial seal (Thermo Scientific, catalog no. 15-111-703).The reaction vessel was then removed from the glovebox, sealed with electrical tape, placed in a photoreactor, and subjected to LED irradiation using two 45 W Kessil PR-160L 427 nm LEDs at 100% intensity placed at distances of 3 cm with vigorous stirring (Figure **SI-2d**). After 18 h, the LEDs were turned off and the reaction mixture was diluted with sat. aq. $NaHCO_3$ (5 mL) and allowed to stir (Figure **SI-2e**). After 10 min, the reaction mixture was transferred to a separatory funnel. The layers were separated and the aqueous layer was extracted with Et_2O (3 x 5 mL). The combined organic layers were washed with brine (5 mL), dried over Na_2SO_4, filtered, and concentrated *in vacuo* with the aid of a rotary evaporator. The residue was then purified by either column chromatography or silica preparative thin-layer chromatography to yield the corresponding product **3**.

4.1.1. General Graphical Procedure C: Photocatalyzed Mizoroki-Heck Cross Coupling with Vinyl Arenes

(a) **(b)**

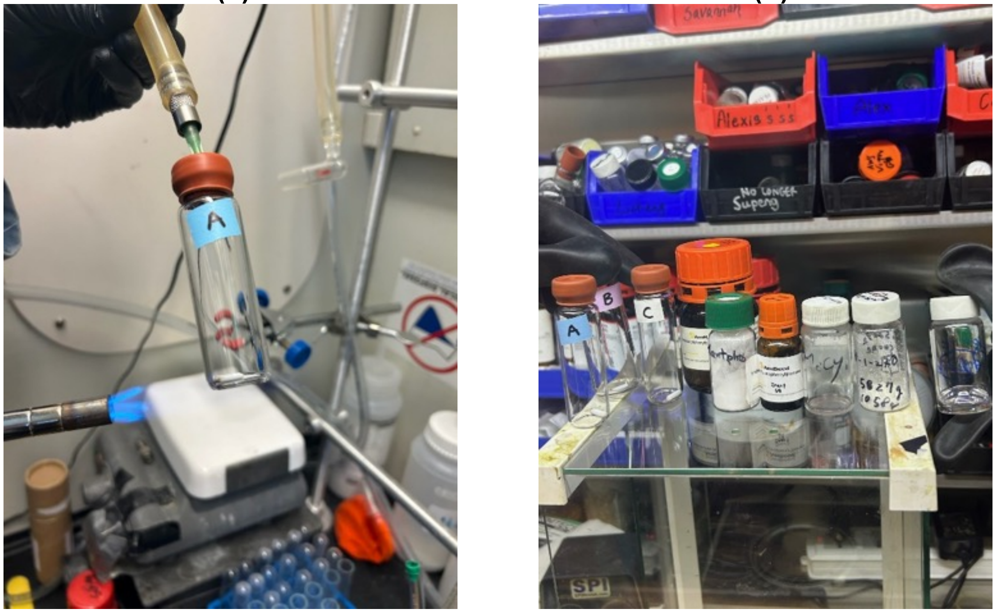

Figure SI-2a: (a) Reaction tubes "**A**–**C**" are each charged with a stir bar and flame-dried. **(b)** Required reagents, left to right: flame-dried reaction tubes "**A**–**C**" and magnetic stir bars, $Pd(PPh_3)_4$, Xantphos, $B(C_6F_5)_3$, $NMeCy_2$, isonitrile, and vinyl arene.

(a) **(b)**

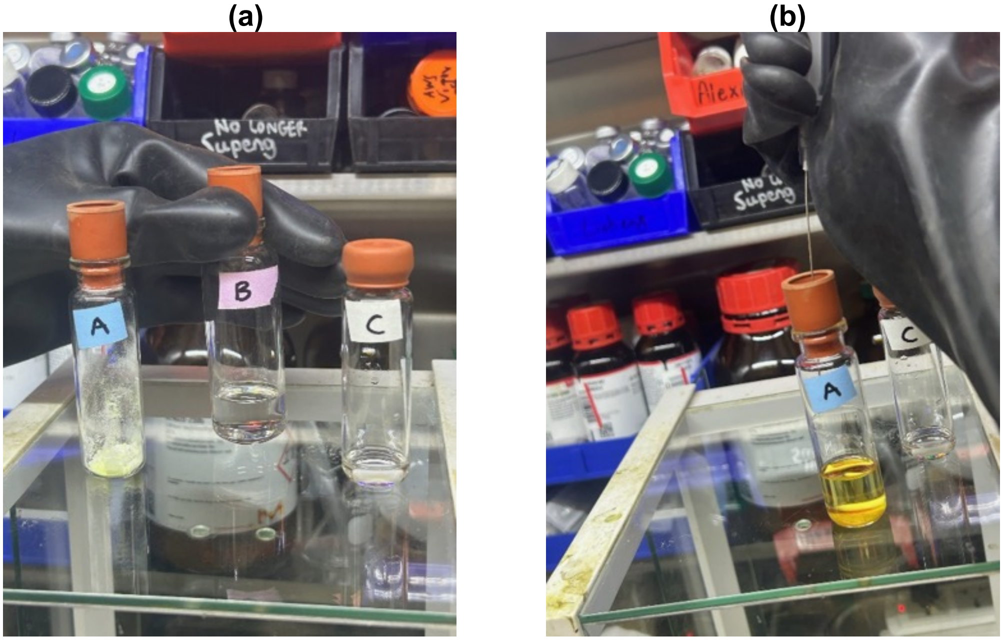

Figure SI-2b: (a) From left to right: Reaction tube "**A**" charged with $Pd(PPh_3)_4$ (35 mg, 0.03 mmol, 10 mol%), Xantphos (52 mg, 0.09 mmol, 30 mol%), $B(C_6F_5)_3$ (154 mg, 0.30 mmol, 1.0 equiv), solid isonitrile (**1**) (0.375 mmol, 1.25 equiv) and/or solid vinyl arene. (**2**) (0.30 mmol, 1.0 equiv). Reaction tube "**B**" was charged with the appropriate solvent. Reaction tube "**C**" containing a solution of $NMeCy_2$ (1.0 M in corresponding solvent from

reaction tube "**B**"). **(b)** The mixture in reaction tube "**A**" is diluted in solvent from reaction tube "**B**" (5.1 mL). If either the isonitrile or the vinyl arene were a liquid, they were added at this point.

(a) **(b)** **(c)**

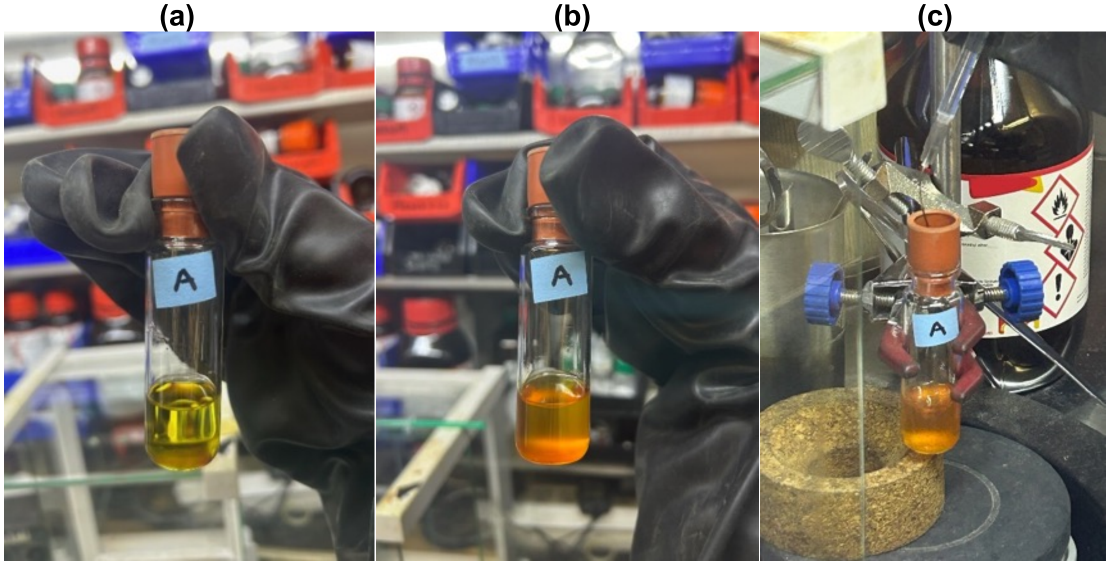

Figure SI-2c: The appearance of the reaction mixture in reaction tube "**A**" immediately before (**a**) and after (**b**) the 10 min stirring period. **(c)** A solution of $NMeCy_2$ (0.9 mL, 0.9 mmol, 3.0 equiv, 1.0 M) from reaction tube "**C**" was added dropwise down the walls of reaction to tube "**A**" over 10 min.

(a) **(b)**

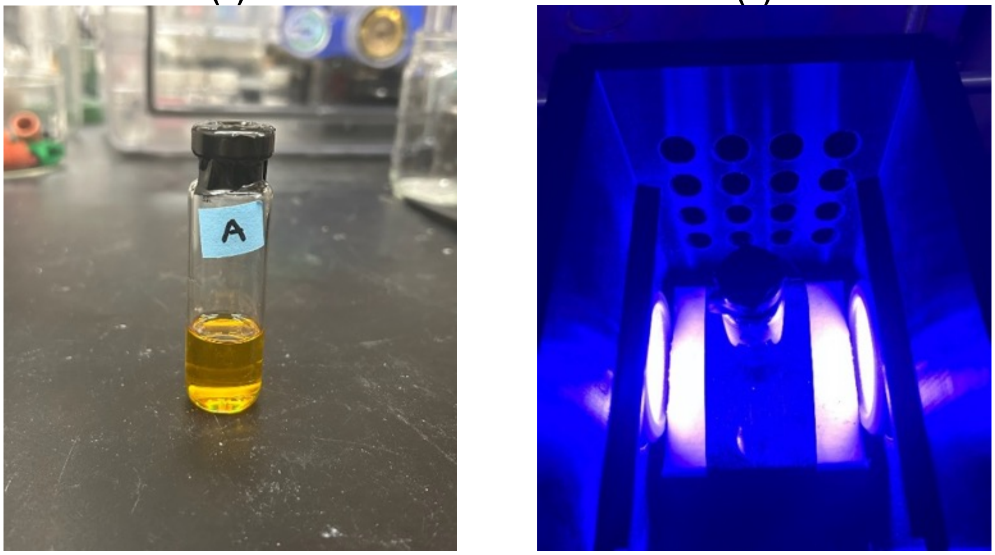

Figure SI-2d: (a) Reaction tube "**A**" was removed from the glovebox and then sealed with electrical tape. **(b)** The reaction vessel was placed in a photoreactor at distances of 3 cm. The two 427 nm LEDs were set to 100% intensity and the 75 mm fan was turned on (reaction temperature: *ca*. 30 ºC).

(a)

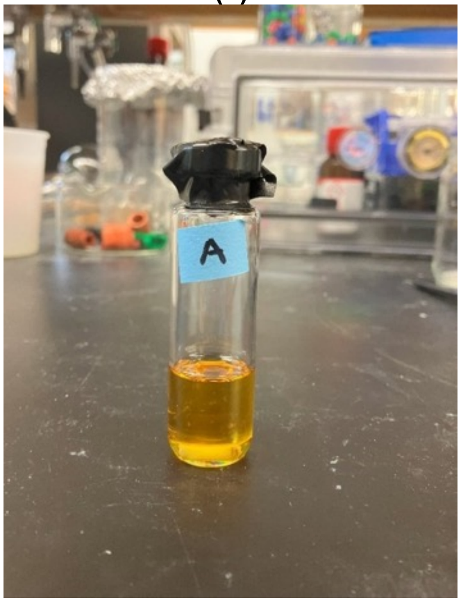

Figure SI-2e: (a) Appearance of reaction tube "**A**" after the reaction mixture had been irradiated for 18 h.

4.2 General Procedure D for Purification by Reversed-Phase Chromatography

The crude residue from **General Procedure C** was dry-loaded onto a 30 g C18 column (prepacked Biotage® Sfär C18 D - Duo 100 Å 30 µm cartridge). The residue was purified using an automated Biotage® Selekt Flash Chromatography System. After the chromatography was complete, the test tubes containing the product were combined into a 500 mL round-bottom flask and the test tubes were each washed with Et_2O (*ca.* 2 x 2 mL). The mixture was concentrated *in vacuo* with the aid of a rotary evaporator to remove the MeCN and Et_2O. Once only the aqueous phase remained, the flask was removed from the rotary evaporator, transferred to a separatory funnel and diluted with Et_2O (30 mL) and H_2O (50 mL). The layers were separated, and the aqueous layer was extracted with Et_2O (3 x 20 mL). The combined organic layers were washed with brine (25 mL), dried over Na_2SO_4, filtered and concentrated *in vacuo* with the aid of a rotary evaporator to yield the corresponding alkylated product.

5. Synthesis and Characterization of Mizoroki-Heck Coupling Products 3a–3v

(3*R*,5*R*,7*R*)-1-((*E*)-Styryl)adamantane (3a)

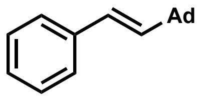

3a

Prepared according to **General Procedure C** using styrene (**2a**) (31 mg, 0.30 mmol) and 1-isocyanoadamantane (**1a**) (61 mg, 0.375 mmol). The residue was purified according to **General Procedure D** (30 g C18, gradient elution: H_2O (0.1% TFA) to MeCN) to yield **3a** as a colorless solid (47 mg, 65%).

All spectroscopic data for **3a** was consistent with that which was previously reported.[11]

^{1}H NMR (600 MHz, $CDCl_3$): δ 7.36 (d, *J* = 8.0 Hz, 2H), 7.28 (t, *J* = 7.6 Hz, 2H), 7.18 (tt, *J* = 7.3, 1.2 Hz, 1H), 6.24c (d, *J* = 16.3 Hz, 1H), 6.11 (d, *J* = 16.3 Hz, 1H), 2.03 (br s, 3H), 1.78–1.67 (m, 12H).

^{13}C NMR (151 MHz, $CDCl_3$): δ 142.3, 138.3, 128.6, 126.8, 126.1, 124.6, 42.4, 37.0, 35.3, 28.6.

IR (Diamond-ATR, neat) ṽ (cm^{-1}): 3022, 2892, 2846, 1656, 1492, 1448, 1096, 965.

(3*R*,5*R*,7*R*)-1-((*E*)-4-Methoxystyryl)adamantane (3b)

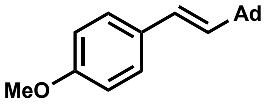

3b

Prepared according to **General Procedure C** using 1-methoxy-4-vinylbenzene (40 mg, 0.30 mmol) and 1-isocyanoadamantane (**1a**) (61 mg, 0.375 mmol). The residue was purified according to **General Procedure D** (30 g C18, gradient elution: H_2O (0.1% TFA) to MeCN) to yield **3b** as a colorless solid (42 mg, 52%).

All spectroscopic data for **3b** was consistent with that which was previously reported.[12]

^{1}H NMR (600 MHz, $CDCl_3$): δ 7.29 (d, *J* = 8.7 Hz, 2H), 6.83 (d, *J* = 8.7 Hz, 2H), 6.18 (d, *J* = 16.3 Hz, 1H), 5.97 (d, *J* = 16.3 Hz, 1H), 3.80 (s, 3H), 2.02 (br s, 3H), 1.77–1.66 (m, 12H).

^{13}C NMR (151 MHz, $CDCl_3$): δ 158.7, 140.3, 131.2, 127.2, 123.9, 114.0, 55.4, 42.5, 37.1, 35.2, 28.7.

IR (Diamond-ATR, neat) ṽ (cm^{-1}): 2989, 2898, 2844, 1605, 1510, 1465, 1179, 1098, 1033, 970.

(*E*)-(4-(2-(Adamantan-1-yl)vinyl)phenyl)(methyl)sulfane (3c)

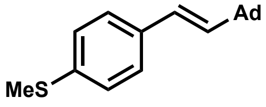

3c

Prepared according to **General Procedure C** using methyl(4-vinylphenyl)sulfane (45 mg, 0.30 mmol) and 1-isocyanoadamantane (**1a**) (61 mg, 0.375 mmol). The residue was purified according to **General Procedure D** (30 g C18, gradient elution: H_2O (0.1% TFA) to MeCN) to yield **3c** as a colorless solid (55 mg, 65%).

^{1}H NMR (600 MHz, $CDCl_3$): δ 7.28 (d, *J* = 8.4 Hz, 2H), 7.19 (d, *J* = 8.4 Hz, 2H), 6.19 (d, *J* = 16.3 Hz, 1H), 6.07 (d, *J* = 16.3 Hz, 1H), 2.47 (s, 3H), 2.02 (br s, 3H), 1.77–1.66 (m, 12H).

^{13}C NMR (151 MHz, $CDCl_3$): δ 141.9, 136.5, 135.6, 127.2, 126.6, 124.0, 42.4, 37.0, 35.3, 28.6, 16.4.

IR (Diamond-ATR, neat) ṽ (cm^{-1}): 2894, 2844, 2369, 2337, 1493, 1448, 1094, 965.

HRMS (ESI): m/z: $[M+H]^+$ calc'd for $C_{19}H_{25}S^+$: 285.1671. Found: 285.1699.

N-(4-((*E*)-2-((3*R*,5*R*,7*R*)-Adamantan-1-yl)vinyl)phenyl)acetamide (3d)

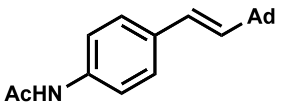

3d

Prepared according to **General Procedure C** using *N*-(4-vinylphenyl)acetamide (**2b**) (48 mg, 0.30 mmol) and 1-isocyanoadamantane (**1a**) (61 mg, 0.375 mmol). The residue was purified according to **General Procedure D** (30 g C18, gradient elution: H_2O (0.1% TFA) to MeCN) to yield **3d** as a colorless solid (40 mg, 45%).

^{1}H NMR (600 MHz, $CDCl_3$): δ 7.42 (d, *J* = 8.5 Hz, 2H), 7.30 (d, *J* = 8.5 Hz, 2H), 7.18 (br s, 1H), 6.19 (d, *J* = 16.3 Hz, 1H), 6.04 (d, *J* = 16.3 Hz, 1H), 2.17 (s, 3H), 2.02 (br s, 3H), 1.77–1.66 (m, 12H).

^{13}C NMR (151 MHz, $CDCl_3$): δ 168.2, 141.6, 136.6, 134.6, 126.6, 123.9, 119.9, 42.4, 37.0, 35.3, 28.6, 24.8.

IR (Diamond-ATR, neat) ṽ (cm^{-1}): 3295, 3250, 3186, 3117, 3024, 2890, 2360, 1665, 1599, 1407, 1323, 1096, 969.

HRMS (ESI): m/z: $[M+H]^+$ calc'd for $C_{20}H_{26}NO^+$: 296.2009. Found: 296.2006

(3*R*,5*R*,7*R*)-1-((*E*)-2-Methoxystyryl)adamantane (3e)

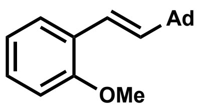

3e

Prepared according to **General Procedure C** using 1-methoxy-2-vinylbenzene (40 mg, 0.30 mmol) and 1-isocyanoadamantane (**1a**) (61 mg, 0.375 mmol). The residue was purified according to **General Procedure D** (30 g C18, gradient elution H_2O (0.1% TFA) to MeCN) to yield **3e** as a colorless solid (56 mg, 69%).

^{1}H NMR (600 MHz, $CDCl_3$): δ 7.45 (dd, *J* = 7.7, 1.7 Hz, 1H), 7.17 (ddd, *J* = 8.3, 7.4 Hz, 1.7 Hz, 1H), 6.90 (t, *J* = 7.4 Hz, 1H), 6.85 (d, *J* = 8.3 Hz, 1H), 6.60 (d, *J* = 16.4 Hz, 1H), 6.09 (d, *J* = 16.4 Hz, 1H), 3.84 (s, 3H), 2.03 (br s, 3H), 1.78–1.67 (m, 12H).

^{13}C NMR (151 MHz, $CDCl_3$): δ 156.5, 142.7, 127.8, 127.3, 126.1, 120.7, 119.0, 110.9, 55.6, 42.4, 37.1, 35.6, 28.7.

IR (Diamond-ATR, neat) ṽ (cm^{-1}): 2898, 2844, 1600, 1487, 1450, 1237, 1103, 1029, 971.

HRMS (ESI): m/z: $[M+H]^+$ calc'd for $C_{19}H_{25}O^+$: 269.1900. Found: 269.1903.

4-(3-((*E*)-2-((3R,5R,7R)-Adamantan-1-yl)vinyl)phenyl)thiomorpholine (3f)

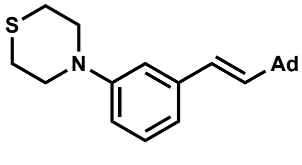

3f

Prepared according to **General Procedure C** using 4-(3-vinylphenyl)thiomorpholine (62 mg, 0.30 mmol) and 1-isocyanoadamantane (**1a**) (61 mg, 0.375 mmol). The residue was purified according to **General Procedure D** (30 g C18, gradient elution: H_2O (0.1% TFA) to MeCN) to yield **3f** as a yellow oil (38 mg, 37%).

^{1}H NMR (600 MHz, $CDCl_3$): δ 7.19 (t, *J* = 7.8 Hz, 1H), 6.95–6.84 (m, 2H), 6.75 (dd, *J* = 8.4, 2.2 Hz, 1H), 6.20 (d, *J* = 16.2 Hz, 1H), 6.08 (d, *J* = 16.2 Hz, 1H), 3.57–3.50 (m, 4H), 2.83-2.67 (m, 4H), 2.03 (br s, 3H), 1.77–1.66 (m, 12H).

^{13}C NMR (151 MHz, $CDCl_3$): δ 151.9, 142.2, 139.4, 129.4, 124.9, 118.0, 116.1, 115.3, 52.5, 42.4, 37.0, 35.3, 28.6, 27.1.

IR (Diamond-ATR, neat) ṽ (cm^{-1}): 2899, 2845, 1593, 1450, 1378, 1283,1169, 966.

HRMS (ESI): m/z: $[M+H]^+$ calc'd for $C_{22}H_{30}NS^+$: 340.2093. Found: 340.2094.

tert-Butyl 4-(4-((*E*)-2-((3*R*,5*R*,7*R*)-adamantan-1-yl)vinyl)phenyl)piperazine-1-carboxylate (3g)

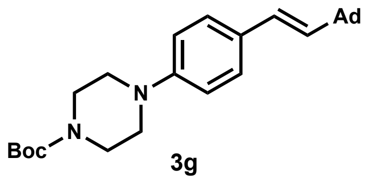

3g

Prepared according to **General Procedure C** using *tert*-butyl 4-(4-vinylphenyl)piperazine-1-carboxylate (87 mg, 0.30 mmol) and 1-isocyanoadamantane (**1a**) (61 mg, 0.375 mmol). The residue was purified according to **General Procedure D** (30 g C18, gradient elution: H_2O (0.1% TFA) to MeCN) to yield **3g** as a colorless solid (62 mg, 49%).

^{1}H NMR (600 MHz, $CDCl_3$): δ 7.28 (d, *J* = 8.7 Hz, 2H), 6.86 (d, *J* = 8.7 Hz, 2H), 6.17 (d, *J* = 16.2 Hz, 1H), 5.97 (d, *J* = 16.2 Hz, 1H), 3.61–3.52 (m, 4H), 3.15–3.06 (m, 4H), 2.01 (br s, 3H), 1.77–1.65 (m, 12H), 1.48 (s, 9H).

^{13}C NMR (151 MHz, $CDCl_3$): δ 154.9, 150.3, 140.1, 130.7, 126.9, 124.0, 116.8, 80.0, 49.7, 42.5, 37.1, 35.2, 28.7, 28.6.

IR (Diamond-ATR, neat) ṽ (cm^{-1}): 2974, 2899, 2845, 1687, 1607, 1514, 1413, 1364, 1228, 1163, 1163, 1120.

HRMS (ESI): m/z: $[M+H]^+$ calc'd for $C_{27}H_{39}N_2O_2^+$: 423.3006. Found: 423.3006.

1-(4-((*E*)-2-((3R,5R,7R)-Adamantan-1-yl)vinyl)phenyl)-1*H*-pyrrole (3h)

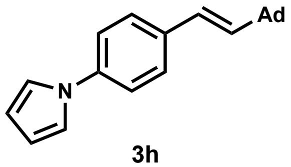

3h

Prepared according to **General Procedure C** using 1-(4-vinylphenyl)-1*H*-pyrrole (51 mg, 0.30 mmol) and 1-isocyanoadamantane (**1a**) (61 mg, 0.375 mmol). An NMR yield was determined by ^{1}H NMR spectroscopy of the crude reaction mixture using CH_2Br_2 (26.1

mg) as the internal standard (49% ^{1}H NMR yield). An analytically pure sample of **3h** was purified by preparative thin-layer chromatography (5% Et_2O in hexanes) to yield **3h** as a colorless solid.

^{1}H NMR (600 MHz, $CDCl_3$): δ 7.40 (d, *J* = 8.4 Hz, 2H), 7.32 (d, *J* = 8.4 Hz, 2H), 7.08 (dd, *J* = 2.6, 2.1 Hz, 2H), 6.34 (dd, *J* = 2.6, 2.1 Hz, 2H), 6.24 (d, *J* = 16.2 Hz, 1H), 6.10 (d, *J* = 16.2 Hz, 1H), 2.04 (br s, 3H), 1.79–1.67 (m, 12H).

^{13}C NMR (151 MHz, $CDCl_3$): δ 142.4, 139.4, 135.9, 127.1, 123.7, 120.6, 119.4, 110.4, 42.4, 37.0, 35.4, 28.6.

IR (Diamond-ATR, neat) ṽ (cm^{-1}): 2913, 2896, 2486,1528, 1449, 1331, 967.

HRMS (ESI): m/z: $[M+H]^+$ calc'd for $C_{22}H_{26}N^+$: 304.2060. Found: 304.2059.

3-((*E*)-2-((3*R*,5*R*,7*R*)-Adamantan-1-yl)vinyl)-1-(2-fluorophenyl)-1*H*-pyrazole (3i)

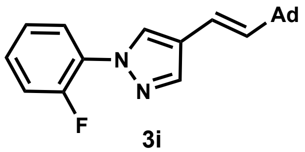

3i

Prepared according to **General Procedure C** using 1-(2-fluorophenyl)-3-vinyl-1*H*-pyrazole (57 mg, 0.30 mmol) and 1-isocyanoadamantane (**1a**) (61 mg, 0.375 mmol). The residue was purified according to **General Procedure D** (30 g C18, gradient elution: H_2O (0.1% TFA) to MeCN) to yield **3i** as colorless solid (48 mg, 50%).

^{1}H NMR (600 MHz, $CDCl_3$): δ 7.92 (d, *J* = 2.9 Hz, 1H), 7.90–7.85 (m, 1H), 7.78 (s, 1H), 7.26–7.19 (m, 3H), 6.13 (d, *J* = 16.4 Hz, 1H), 5.95 (d, *J* = 16.4 Hz, 1H), 2.03 (br s, 3H), 1.78–1.65 (m, 12H).

^{13}C NMR (151 MHz, $CDCl_3$): δ 153.4 (d, *J* = 248.7 Hz), 141.8, 138.8, 128.5 (d, *J* = 9.2 Hz), 127.7 (d, *J* = 10.6 Hz), 127.5 (d, *J* = 7.8 Hz), 125.0 (d, *J* = 3.3 Hz), 124.1, 123.1, 117.0 (d, *J* = 20.5 Hz), 113.9, 42.4, 37.0, 35.3, 28.6.

^{19}F NMR (471 MHz, $CDCl_3$): δ −125.1.

IR (Diamond-ATR, neat) ṽ (cm^{-1}): 2897, 2844, 1615, 1558, 1503, 1468, 1405, 1230, 1109, 950.

HRMS (ESI): m/z: $[M+H]^+$ calc'd for $C_{21}H_{24}FN_2^+$: 323.1918. Found: 323.1918.

tert-Butyl-5-((_E_)-2-((3_R_,5_R_,7_R_)-adamantan-1-yl)vinyl)-1_H_-indole-1-carboxylate (3j)

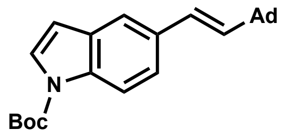

3j

Prepared according to **General Procedure C** using *tert*-butyl 5-vinyl-1*H*-indole-1-carboxylate (73 mg, 0.30 mmol) and 1-isocyanoadamantane (**1a**) (61 mg, 0.375 mmol). The residue was purified according to **General Procedure D** (30 g C18, gradient elution: H_2O (0.1% TFA) to MeCN) to yield **3j** as colorless solid (62 mg, 55%).

^{1}H NMR (600 MHz, $CDCl_3$): δ 8.03 (br s, 1H), 7.54 (s, 1H), 7.52 (d, *J* = 1.3 Hz, 1H), 7.35 (dd, *J* = 8.6, 1.3 Hz, 1H), 6.52 (d, *J* = 3.8 Hz, 1H), 6.32 (d, *J* = 16.3 Hz, 1H), 6.11 (d, *J* = 16.3 Hz, 1H), 2.03 (br s, 3H), 1.80–1.70 (m, 12H), 1.67 (s, 9H).

^{13}C NMR (151 MHz, $CDCl_3$): δ 149.9, 141.1, 133.2, 131.0, 127.7, 126.3, 124.8, 122.7, 118.4, 115.2, 107.5, 83.7, 42.5, 37.1, 35.3, 28.7, 28.4.

IR (Diamond-ATR, neat) ṽ (cm^{-1}): 2983, 2899, 2851, 1730, 1467, 1368, 1347, 1329, 1159, 1125, 1081, 1022, 965.

HRMS (ESI): m/z: $[M+H]^+$ calc'd for $C_{25}H_{32}NO_2^+$: 378.2428. Found: 378.2426.

3-((_E_)-2-((3_R_,5_R_,7_R_)-Adamantan-1-yl)vinyl)thiophene (3k)

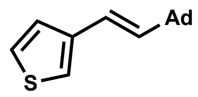

3k

Prepared according to **General Procedure C** using 3-vinylthiophene (34 mg, 0.30 mmol) and 1-isocyanoadamantane (**1a**) (61 mg, 0.375 mmol). The residue was purified according to **General Procedure D** (30 g C18, gradient elution: H_2O (0.1% TFA) to MeCN) to yield **3k** as colorless solid (34 mg, 46%).

^{1}H NMR (600 MHz, $CDCl_3$): δ 7.25 (dd, *J* = 5.1, 2.9 Hz, 1H), 7.20 (dd, *J* = 5.1, 1.1 Hz, 1H), 7.06 (dd, *J* = 2.9, 1.1 Hz, 1H), 6.26 (d, *J* = 16.2 Hz, 1H), 5.97 (d, *J* = 16.2 Hz, 1H), 2.02 (br s, 3H), 1.77–1.65 (m, 12H).

^{13}C NMR (151 MHz, $CDCl_3$): δ 142.3, 140.9, 125.9, 125.1, 120.5, 119.1, 42.4, 37.0, 35.2, 28.6.

IR (Diamond-ATR, neat) ṽ (cm^{-1}): 2897, 2844, 1450, 1106, 962.

HRMS (ESI): m/z: $[M+H]^+$ calc'd for $C_{16}H_{21}S^+$: 245.1358. Found: 245.1358.

(*3R,5R,7R*)-1-(2-phenylallyl)adamantane (3l)

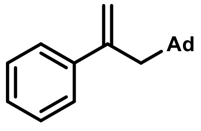

3l

Prepared according to **General Procedure C** using α-methylstyrene (36 mg, 0.30 mmol) and 1-isocyanoadamantane (**1a**) (61 mg, 0.375 mmol). An NMR yield was determined by ^{1}H NMR spectroscopy of the crude reaction mixture using CH_2Br_2 (26.1 mg) as the internal standard (48% ^{1}H NMR yield). An analytically pure sample of **3l** was purified by flash column chromatography on silica gel (hexanes) and subsequently purified according to **General Procedure D** (30 g C18, gradient elution: H_2O (0.1% TFA) to MeCN) to yield **3l** as a colorless solid.

All spectroscopic data for **3l** was consistent with that which was previously reported.[13]

^{1}H NMR (600 MHz, $CDCl_3$): δ 7.40 (d, *J* = 7.8 Hz, 2H), 7.30 (t, *J* = 7.4 Hz, 2H), 7.23 (t, *J* = 7.3 Hz, 1H), 5.26 (d, *J* = 2.0 Hz, 1H), 4.98 (d, *J* = 2.0 Hz, 1H), 2.34 (s, 2H), 1.86 (br s, 3H), 1.64–1.60 (m, 3H), 1.55–1.50 (m, 3H), 1.39–1.37 (m, 6H).

^{13}C NMR (151 MHz, $CDCl_3$): δ 146.1, 144.0, 128.2, 127.0, 126.6, 116.2, 49.9, 43.1, 37.1, 33.8, 28.9.

(*E*)-*N*-(4-(3,3-Dimethylbut-1-en-1-yl)phenyl)acetamide (3m)

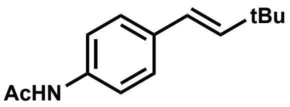

3m

Prepared according to **General Procedure C** using *N*-(4-vinylphenyl)acetamide (**2b**) (48 mg, 0.30 mmol) and 2-isocyano-2-methylpropane (**1b**) (31 mg, 0.375 mmol). An NMR yield was determined by ^{1}H NMR spectroscopy of the crude reaction mixture using CH_2Br_2 (26.1 mg) as the internal standard (42% ^{1}H NMR yield). An analytically pure sample of **3m** was purified by flash column chromatography on silica gel (gradient elution: 25% EtOAc in hexanes to 50% EtOAc in hexanes) to yield **3m** as a colorless solid.

^{1}H NMR (600 MHz, $CDCl_3$): δ 7.43 (d, *J* = 8.4 Hz, 2H), 7.31 (d, *J* = 8.4 Hz, 2H), 7.12 (br s, 1H), 6.26 (d, *J* = 16.1 Hz, 1H), 6.18 (d, *J* = 16.1 Hz, 1H), 2.17 (s, 3H), 1.11 (s, 9H).

^{13}C NMR (151 MHz, $CDCl_3$): δ 168.5, 141.3, 136.7, 134.4, 126.6, 124.0, 120.0, 33.5, 29.7, 24.7.

HRMS (ESI): m/z: $[M+H]^+$ calc'd for $C_{14}H_{20}NO^+$: 218.1539. Found: 218.1536.

(*E*)-1-(4-(3,3-Dimethylbut-1-en-1-yl)phenyl)-1*H*-pyrrole (3n)

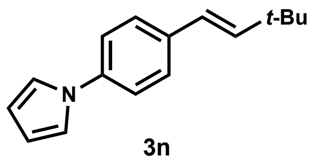

3n

Prepared according to **General Procedure C** using 1-(4-vinylphenyl)-1*H*-pyrrole (51 mg, 0.30 mmol) and 2-isocyano-2-methylpropane (**1b**) (31 mg, 0.375 mmol). The residue was purified according to **General Procedure D** (30 g C18, gradient elution: H_2O (0.1% TFA) to MeCN) to yield **3n** as colorless solid (35 mg, 52%).

^{1}H NMR (600 MHz, $CDCl_3$): δ 7.41 (d, *J* = 8.6 Hz, 2H), 7.33 (d, *J* = 8.6 Hz, 2H), 7.09 (at, *J* = 2.2 Hz, 2H), 6.35 (at, *J* = 2.2 Hz, 2H), 6.31 (d, *J* = 16.2 Hz, 1H), 6.25 (d, *J* = 16.2 Hz, 1H), 1.14 (s, 9H).

^{13}C NMR (151 MHz, $CDCl_3$): δ 142.1, 139.4, 135.7, 127.2, 123.7, 120.6, 119.4, 110.4, 33.6, 29.7.

IR (Diamond-ATR, neat) ṽ (cm^{-1}): 3048, 2959, 2906, 1608, 1521, 1480, 1330, 813.

HRMS (ESI): m/z: $[M+H]^+$ calc'd for $C_{16}H_{20}N^+$: 226.1590. Found: 226.1586.

(*E*)-5-(3,3-Dimethylbut-1-en-1-yl)benzo[*d*][1,3]dioxole (3o)

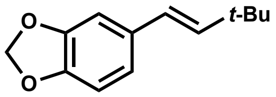

3o

Prepared according to **General Procedure C** using 5-vinylbenzo[*d*][1,3]dioxole (44 mg, 0.30 mmol) and 2-isocyano-2-methylpropane (**1b**) (31 mg, 0.375 mmol). The residue was purified according to **General Procedure D** (30 g C18, gradient elution: H_2O (0.1% TFA) to MeCN) to yield **3o** as pale yellow solid (28 mg, 46%).

^{1}H NMR (600 MHz, $CDCl_3$): δ 6.92 (d, *J* = 1.5 Hz, 1H), 6.78 (dd, *J* = 8.0, 1.5 Hz, 1H), 6.74 (d, *J* = 8.0 Hz, 1H), 6.21 (d, *J* = 16.1 Hz, 1H), 6.09 (d, *J* = 16.1 Hz, 1H), 5.93 (s, 2H), 1.10 (s, 9H).

^{13}C NMR (151 MHz, $CDCl_3$): δ 148.1, 146.6, 140.4, 132.7, 124.3, 120.5, 108.3, 105.6, 101.0, 33.4, 29.8.

IR (Diamond-ATR, neat) ṽ (cm^{-1}): 3028, 2956, 2778, 1607, 1502, 1489, 1236.

HRMS (ESI): m/z: $[M+H]^+$ calc'd for $C_{13}H_{17}O_2^+$: 205.1223. Found: 205.1220.

(*E*)-3,7,7-Trimethyl-9-phenylnon-8-en-1-yl 6-(trifluoromethyl)nicotinate (3p)

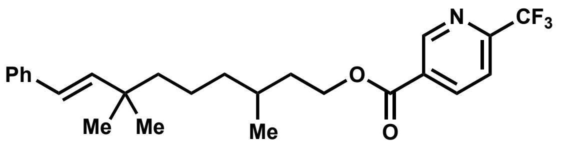

3p

Prepared according to **General Procedure C** using styrene (**2a**) (31 mg, 0.30 mmol) and 7-isocyano-3,7-dimethyloctyl 6-(trifluoromethyl)nicotinate (**SI-8**) (134 mg, 0.375 mmol). The residue was purified by silica preparative thin-layer chromatography (5% Et_2O in hexanes) to yield **3p** as a colorless oil (55 mg, 42%).

^{1}H NMR (600 MHz, $CDCl_3$): δ 9.29 (d, *J* = 1.7 Hz, 1H), 8.45 (dd, *J* = 8.1, 1.7 Hz, 1H), 7.75 (d, *J* = 8.1 Hz, 1H), 7.36 (d, *J* = 7.3 Hz, 2H), 7.29 (t, *J* = 7.8 Hz, 2H), 7.18 (tt, *J* = 7.3, 1.3 Hz, 1H), 6.27 (d, *J* = 16.2 Hz, 1H), 6.17 (d, *J* = 16.2 Hz, 1H), 4.47–4.37 (m, 2H), 1.86–1.78 (m, 1H), 1.66–1.56 (m, 2H), 1.40–1.28 (m, 4H), 1.28–1.22 (m, 1H), 1.22–1.15 (m, 1H), 1.09 (s, 6H), 0.95 (d, *J* = 6.5 Hz, 3H).

^{13}C NMR (151 MHz, $CDCl_3$): δ 164.2, 151.3 (q, *J* = 35.1 Hz), 151.1, 140.8, 138.8, 138.2, 128.9, 128.6, 126.9, 126.1, 125.9, 121.2 (q, *J* = 274.6 Hz), 120.3 (q, *J* = 2.5 Hz), 64.8, 43.6, 37.8, 36.4, 35.6, 30.1, 27.4, 27.3, 22.1, 19.7.

^{19}F NMR (471 MHz, CDCl3): δ −68.2.

IR (Diamond-ATR, neat) ṽ (cm^{-1}): 3028, 2957, 2930, 1726, 1600, 1462, 1332, 1281, 1118, 1084, 1023.

HRMS (ESI): m/z: $[M+H]^+$ calc'd for $C_{25}H_{31}F_3NO_2^+$: 434.2301. Found: 434.2298.

Methyl (*E*)-4-styryl)bicyclo[2.2.2]octane-1-carboxylate (3q)

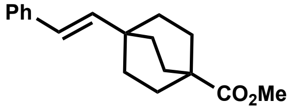

3q

Prepared according to **General Procedure C** using styrene (**2a**) (31 mg, 0.30 mmol) and methyl 4-isocyanobicyclo[2.2.2]octane-1-carboxylate (73 mg, 0.375 mmol). The residue was purified according to **General Procedure D** (30 g C18, gradient elution: H_2O (0.1% TFA) to MeCN) and subsequently silica preparative thin-layer chromatography (20% EtOAc in hexanes) to yield **3q** as colorless solid (29 mg, 36%).

All spectroscopic data for **3q** was consistent with that which was previously reported.[14]

^{1}H NMR (600 MHz, $CDCl_3$): δ 7.33 (d, *J* = 7.9 Hz, 2H), 7.28 (t, *J* = 7.9 Hz, 2H), 7.19 (t, *J* = 7.3 Hz, 1H), 6.24 (d, *J* = 16.3 Hz, 1H), 6.13 (d, *J* = 16.3 Hz, 1H), 3.66 (s, 3H), 1.89–1.81 (m, 6H), 1.67–1.59 (m, 6H).

^{13}C NMR (151 MHz, $CDCl_3$): δ 178.6, 139.3, 138.0, 128.6, 127.1, 126.1, 126.0, 51.8, 39.3, 33.4, 30.9, 28.5.

IR (Diamond-ATR, neat) ṽ (cm^{-1}): 3021, 2949, 2914, 2859, 2359, 2341, 1730, 1237, 1071.

(*E*)-(2-Cyclohexylvinyl)benzene (3r)

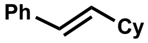

3r

Prepared according to **General Procedure C** using styrene (**2a**) (31 mg, 0.30 mmol) and isocyanocyclohexane (41 mg, 0.375 mmol). Due to volatility of the product, an NMR yield was determined by ^{1}H NMR spectroscopy of the crude reaction mixture using CH_2Br_2 (26.1 mg) as the internal standard (30% ^{1}H NMR yield). An analytically pure sample of **3r** was purified by silica preparative thin-layer chromatography (pentane) and isolated as a colorless oil.

All spectroscopic data for **3r** was consistent with that which was previously reported.[15]

^{1}H NMR (600 MHz, $CDCl_3$): δ 7.36–7.33 (m, 2H), 7.28 (t, *J* = 7.7 Hz, 2H), 7.18 (tt, *J* = 7.3, 1.3 Hz, 1H), 6.34 (d, *J* = 16.0 Hz, 1H), 6.18 (dd, *J* = 16.0, 7.0 Hz, 1H), 2.16–2.09 (m, 1H), 1.83–1.74 (m, 4H), 1.71–1.65 (m, 1H), 1.32 (qt, *J* = 12.8, 3.3 Hz, 2H), 1.24–1.14 (m, 3H).

^{13}C NMR (151 MHz, $CDCl_3$): δ 138.2, 137.0, 128.6, 127.3, 126.9, 126.1, 41.3, 33.1, 26.3, 26.2.

IR (Diamond-ATR, neat) ṽ (cm^{-1}): 2954, 2926, 2857, 1729, 1600, 1332, 1120.

(*E*)-*tert*-Butyldimethyl((3,7,7-trimethyl-9-phenylnon-8-en-1-yl)oxy)silane (3s)

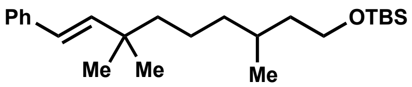

3s

Prepared according to **General Procedure C** using styrene (**2a**) (31 mg, 0.30 mmol) and *tert*-butyl((7-isocyano-3,7-dimethyloctyl)oxy)dimethylsilane (**SI-7**) (112 mg, 0.375 mmol). The residue was purified according to **General Procedure D** (30 g C18, gradient elution: H_2O (0.1% TFA) to MeCN) and subsequently silica preparative thin-layer chromatography (30% CH_2Cl_2 in hexanes) to yield **3s** as colorless oil (62 mg, 55%).

^{1}H NMR (500 MHz, $CDCl_3$): δ 7.36 (d, *J* = 7.6 Hz, 2H), 7.29 (t, *J* = 7.6 Hz, 2H), 7.18 (t, *J* = 7.0 Hz, 1H), 6.27 (d, *J* = 16.3 Hz, 1H), 6.18 (d, *J* = 16.3 Hz, 1H), 3.70–3.56 (m, 2H), 1.53–1.47 (m, 1H), 1.39–1.17 (m, 8H), 1.08 (s, 6H), 0.89 (s, 9H), 0.85 (d, *J* = 6.3 Hz, 3H), 0.04 (s, 6H).

^{13}C NMR (126 MHz, $CDCl_3$): δ 141.1, 138.3, 128.6, 126.8, 126.2, 125.7, 61.7, 43.7, 40.1, 38.0, 36.4, 29.6, 27.4, 27.4, 26.1, 22.2, 19.9, 18.5.

IR (Diamond-ATR, neat) ṽ (cm^{-1}): 3025, 2954, 2927, 2856, 1471, 1449, 1384, 1361, 1254, 1094, 834, 810.

HRMS (ESI): m/z: $[M+H]^+$ calc'd for $C_{24}H_{43}OSi^+$: 375.3078. Found: 375.3077

(*E*)-3,7,7-Trimethyl-9-(thiophen-3-yl)non-8-en-1-yl 5-methylthiophene-2-carboxylate (3t)

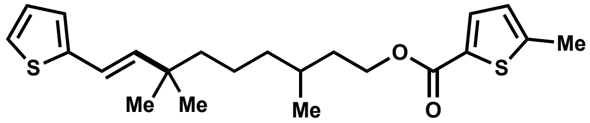

3t

Prepared according to **General Procedure C** using 3-vinylthiophene (33 mg, 0.30 mmol) and 7-isocyano-3,7-dimethyloctyl 5-methylthiophene-2-carboxylate (**SI-9**) (184 mg, 0.60 mmol). The residue was purified according to **General Procedure D** (30 g C18, gradient elution: H_2O (0.1% TFA) to MeCN) to yield **3t** as a yellow oil (53 mg, 45%).

^{1}H NMR (600 MHz, $CDCl_3$): δ 7.59 (d, *J* = 3.7 Hz, 1H), 7.24 (dd, *J* = 5.1, 2.9 Hz, 1H), 7.20 (dd, *J* = 5.1, 1.0 Hz, 1H), 7.06 (d, *J* = 2.9 Hz, 1H), 6.75 (dd, *J* = 3.7, 1.0 Hz, 1H), 6.29 (d, *J* = 16.3 Hz, 1H), 6.04 (d, *J* = 16.3 Hz, 1H), 4.34–4.24 (m, 2H), 2.51 (s, 3H), 1.80–1.70 (m, 1H), 1.64–1.58 (m, 1H), 1.55–1.46 (m, 1H), 1.38–1.20 (m, 6H), 1.06 (s, 6H), 0.92 (d, *J* = 6.7 Hz, 3H).

^{13}C NMR (151 MHz, $CDCl_3$): δ 162.5, 147.9, 140.9, 140.8, 133.8, 131.5, 126.4, 125.9, 125.1, 120.5, 120.2, 63.6, 43.5, 37.8, 36.3, 35.7, 30.0, 27.4, 22.1, 19.7, 15.9.

IR (Diamond-ATR, neat) ṽ (cm^{-1}): 2956, 2928, 2866, 2358, 1706, 1463, 1282, 1259, 1091.

HRMS (ESI): m/z: $[M+H]^+$ calc'd for $C_{22}H_{31}O_2S_2^+$: 391.1760. Found: 391.1760.

(*E*)-3,7,7-Trimethyl-9-(4-(methylthio)phenyl)non-8-en-1-yl benzoate (3u)

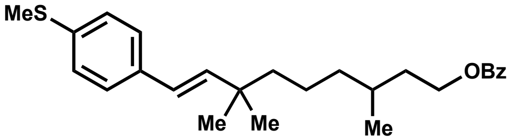

3u

Prepared according to **General Procedure C** using methyl(4-vinylphenyl)sulfane (45 mg, 0.30 mmol) and 7-isocyano-3,7-dimethyloctyl benzoate (108 mg, 0.375 mmol). The residue was purified according to **General Procedure D** (30 g C18, gradient elution: H_2O (0.1% TFA) to MeCN) to yield **3u** as a yellow oil (48 mg, 39%).

^{1}H NMR (600 MHz, $CDCl_3$): δ 8.03 (dd, *J* = 8.2, 1.3 Hz, 2H), 7.55 (tt, *J* = 7.7, 1.3 Hz, 1H), 7.43 (t, *J* = 7.7 Hz, 2H), 7.28 (d, *J* = 8.3 Hz, 2H), 7.19 (d, *J* = 8.3 Hz, 2H), 6.22 (d, *J* = 16.2 Hz, 1H), 6.13 (d, *J* = 16.2 Hz, 1H), 4.39–4.29 (m, 2H), 2.47 (s, 3H), 1.83–1.75 (m, 1H), 1.67–1.59 (m, 1H), 1.59–1.55 (m, 1H), 1.37–1.28 (m, 4H), 1.27–1.22 (m, 1H), 1.20–1.13 (m, 1H), 1.07 (s, 6H), 0.94 (d, *J* = 6.8 Hz, 3H).

^{13}C NMR (151 MHz, $CDCl_3$): δ 166.8, 140.5, 136.6, 135.4, 132.9, 130.7, 129.7, 128.5, 127.2, 126.6, 125.2, 63.7, 43.6, 37.8, 36.4, 35.7, 30.0, 27.4, 27.4, 22.1, 19.8, 16.3.

IR (Diamond-ATR, neat) ṽ (cm^{-1}): 3074, 3030, 2956, 2928, 1716, 1601, 1271, 1112.

HRMS (ESI): m/z: $[M+H]^+$ calc'd for. $C_{26}H_{35}O_2S^+$: 411.2352. Found: 411.2349.

(E)-(4-(5-(3-Fluoro-4-methylphenyl)-3,3-dimethylpent-1-en1yl)phenyl)(methyl) sulfane (3v)

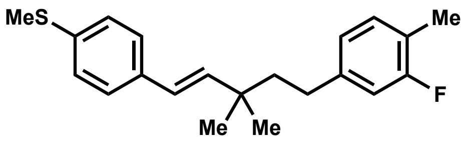

3v

Prepared according to **General Procedure C** using methyl(4-vinylphenyl)sulfane (45 mg, 0.30 mmol) and 2-fluoro-4-(3-isocyano-3-methylbutyl)-1-methylbenzene (**SI-5**) (77 mg, 0.375 mmol). The residue was purified according to **General Procedure D** (30 g C18, gradient elution: H_2O (0.1% TFA) to MeCN) to yield **3v** as a colorless oil (45 mg, 45%).

^{1}H NMR (600 MHz, $CDCl_3$): δ 7.30 (d, J = 8.4 Hz, 2H), 7.21 (d, J = 8.4 Hz, 2H), 7.05 (t, J = 8.0 Hz, 1H), 6.85–6.79 (m, 2H), 6.28 (d, J = 16.2 Hz, 1H), 6.17 (d, J = 16.2 Hz, 1H), 2.54–2.50 (m, 2H), 2.49 (s, 3H), 2.22 (d, J = 1.5 Hz, 3H), 1.69–1.64 (m, 2H), 1.16 (s, 6H).

^{13}C NMR (151 MHz, $CDCl_3$): δ 161.4 (d, J = 243.9 Hz), 143.0 (d, J = 7.2 Hz), 139.7, 136.9, 135.1, 131.3 (d, J = 5.5 Hz), 127.1, 126.6, 125.8, 123.7 (d, J = 3.1 Hz), 121.8 (d, J = 17.1 Hz), 114.8 (d, J = 21.6 Hz), 45.2, 36.6, 30.9, 27.4, 16.3, 14.3 (d, J = 3.4 Hz).

^{19}F NMR (471 MHz, CDCl3): δ −118.3.

IR (Diamond-ATR, neat) ṽ (cm^{-1}): 3022, 2956, 2922, 2861, 1628, 1579, 1511, 1252, 1111, 969, 803.

HRMS (ESI): m/z: $[M+H]^+$ calc'd for $C_{21}H_{26}FS^+$: 329.1734. Found: 329.1736.

5.1. General Procedure E: Large-Scale Photocatalyzed Mizoroki-Heck Cross Coupling with Vinyl Arenes

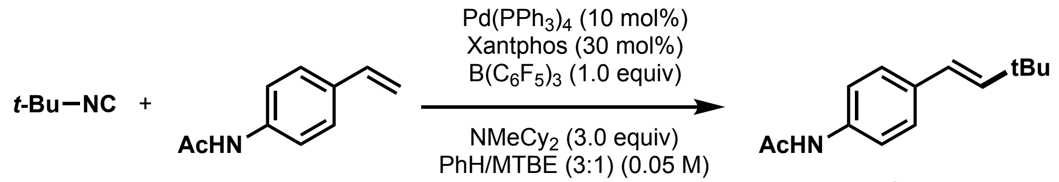

1b (1.25 equiv) **2b** (1.0 equiv) **3m** 268 mg, 41%

Inside a N_2-filled glovebox, a flame-dried 250 mL round-bottom flask "**A**" equipped with a magnetic stir bar was charged with $Pd(PPh_3)_4$ (0.35 g, 0.30 mmol, 10 mol%), Xantphos (0.52 g, 0.90 mmol, 30 mol%), $B(C_6F_5)_3$ (1.5 g, 3.0 mmol, 1.0 equiv), and *N*-(4-vinylphenyl)acetamide (**2b**) (0.48 g, 3.0 mmol, 1.0 equiv). Reaction flask "**A**" was fitted with a rubber septum and then removed from the glovebox. Reaction flask "**A**" was connected to a dual manifold Schlenk line and then evacuated/backfilled with N_2 (this process was repeated a total of three times). At this point, in a flame-dried 50 mL round-bottom flask "**B**" equipped with a magnetic stir bar, a solvent solution of PhH/MTBE (3:1) was prepared.

Then, the reaction flask "**A**" was diluted in the solvent from flask "**B**" (51 mL), and 2-isocyano-2-methylpropane (**1b**) (0.31 g, 0.42 mL, 3.75 mmol, 1.25 equiv) was added to reaction flask "**A**" via syringe. The reaction mixture was allowed to stir for 10 min. During this period, a flame-dried round-bottom flask "**C**" equipped with a magnetic stir bar was charged with $NMeCy_2$ (1.95 g, 10.0 mmol, 3.3 equiv) and diluted with the solvent from flask "**B**" (10 mL, 1.0 M), and allowed to stir.

After the reaction mixture had stirred for 10 min, the solution of $NMeCy_2$ from reaction flask "**C**" (9.0 mL, 9.0 mmol, 3.0 equiv, 1.0 M) was added dropwise down the walls of reaction flask "**A**" over *ca.* 75 min via a syringe pump (Braintree Scientific, Inc., BS-300) (Figure **SI-3a**).The reaction mixture was subjected to LED irradiation using four 45 W Kessil PR-160L 427 nm LEDs at 100% intensity placed at distances of 5 cm (Figure **SI-3b**) with vigorous stirring. After 18 h, the LEDs were turned off and the reaction mixture was diluted with sat. aq. $NaHCO_3$ (50 mL) and allowed to stir. After 10 min, the reaction mixture was transferred to a separatory funnel. The layers were separated and the aqueous layer was extracted with Et_2O (3 x 50 mL). The combined organic layers were washed with brine (50 mL), dried over Na_2SO_4, filtered, and concentrated *in vacuo* with the aid of a rotary evaporator. The residue was purified by flash column chromatography on silica gel (gradient elution: hexanes to 22% EtOAc in hexanes) followed by aqueous extraction with 1 M HCl (3 x 10 mL) to yield **3m** as a colorless solid (268 mg, 41%).

5.1.1. Graphical General Procedure E: Large-Scale Photocatalyzed Mizoroki-Heck Cross Coupling with Vinyl Arenes

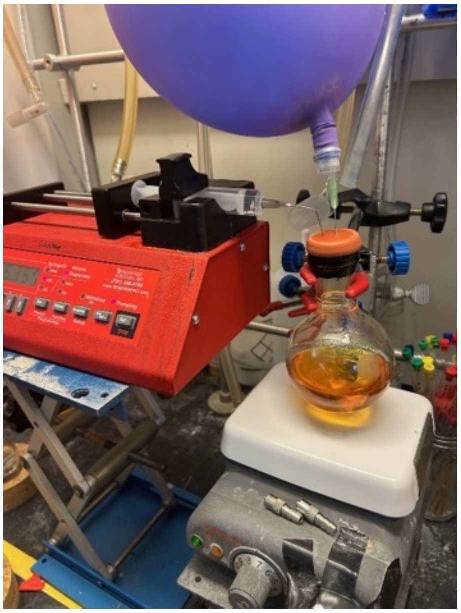

Figure SI-3a: The solution of $NMeCy_2$ (9.0 mL, 9.0 mmol, 3.0 equiv, 1.0 M) was added dropwise down the walls of reaction flask “**A**” over ca. 75 min via a syringe pump.

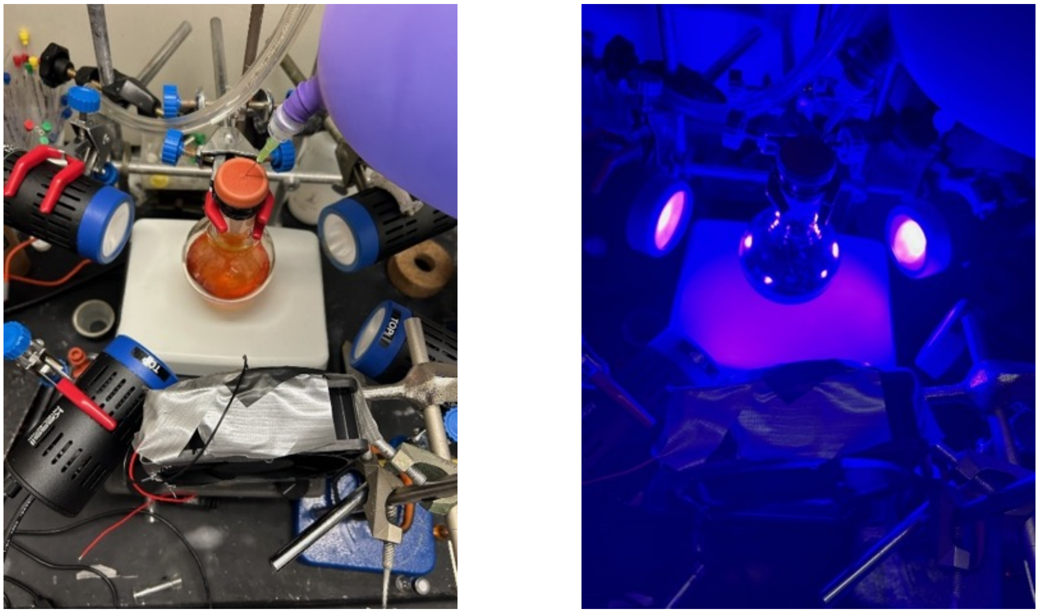

Figure SI-3b: Left: The reaction flask was placed 5 cm away from four 45 W Kessil PR-160L 427 nm LEDs. Right: The reaction mixture was subjected to LED irradiation at 100% intensity with vigorous stirring for 18 h.

6. Mechanistic Experiments
6.1 Cyclic Voltammetry (CV) Experiments

Procedure for Acquiring Cyclic Voltammograms:

To a flame-dried 25 mL three-neck round-bottom flask equipped with a magnetic stir bar was added tetra-*n*-butylammonium hexafluorophosphate (190 mg, 0.50 mmol, 3.4 equiv). The flask was fitted with three rubber septa and and connected to a dual manifold Schlenk line. The flask was then evacuated/backfilled with N_2 once. Then, MeCN (9.87 mL, 0.015 M) and cyclohexyl isocyanide (16 mg, 0.15 mmol, 1.0 equiv) were added via syringe. The resulting mixture was stirred vigorously for 5 min before the septa were removed. A glassy carbon working electrode, a platinum wire counter electrode, and an $Ag/AgNO_3$ reference electrode were attached through the three necks of the flask. The electrodes were connected to a Pine Research WaveDriver 40 DC Bipotentiostat through steel alligator clips, the stirring was stopped and the acquisition of the cyclic voltammogram was initiated. Cyclic voltammogram acquisition was performed at a rate of 50 mV/s, starting at 0 mV, with an initial rising segment to +2000 mV, followed by a falling segment to −2000 mV, and finally a rising segment to 0 mV. The resulting cyclic voltammograms were calibrated to ferrocenium/ferrocene by adding a small portion (*ca.* 2 mg) of ferrocene to the mixture, followed by stirring for 1 min before acquiring the cyclic voltammogram in the same manner. The cyclic voltammogram for the mixture of cyclohexyl isocyanide and $B(C_6F_5)_3$ (76 mg, 0.15 mmol, 1.0 equiv) was also acquired in the same manner.

Results show that cyclohexyl isocyanide is reduced at $E_{p,c}$ = −1.38 V vs Fc^+/Fc (**Figure SI-4a)** and the mixture of cyclohexyl isocyanide and $B(C_6F_5)_3$ is reduced at $E_{p,c}$ = −1.1 V vs Fc^+/Fc (**Figure SI-4b**). This implies that $B(C_6F_5)_3$ slightly lowered the reduction potential of cyclohexyl isocyanide, which may facilitate C–N bond cleavage.

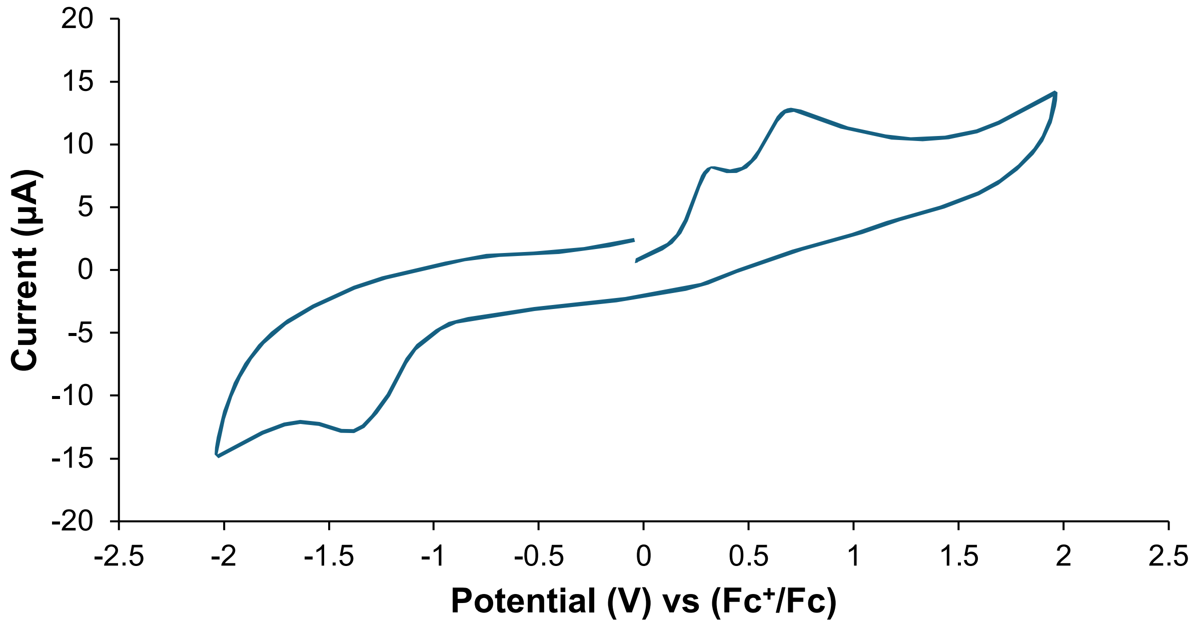

Figure SI-4a: Cyclic voltammogram of cyclohexyl isocyanide. Data was plotted according to the IUPAC convention. Voltammograms recorded using a reaction mixture of cyclohexyl isocyanide (16 mg, 0.15 mmol, 1.0 equiv, 0.015 M) and tetra-*n*-butylammonium hexafluorophosphate (190 mg, 0.50 mmol, 3.4 equiv, 0.05 M) in MeCN (9.87 mL, 0.015 M). The plot was acquired using ν = 50 mV/s with a glassy carbon working electrode, a platinum wire counter electrode, and an $Ag/AgNO_3$ reference electrode. Acquisition was performed starting at 0 mV, with an initial rising segment to +2000 mV, followed by a falling segment to −2000 mV, and finally a rising segment to 0 mV. The resulting cyclic voltammograms were calibrated according to ferrocenium/ferrocene.

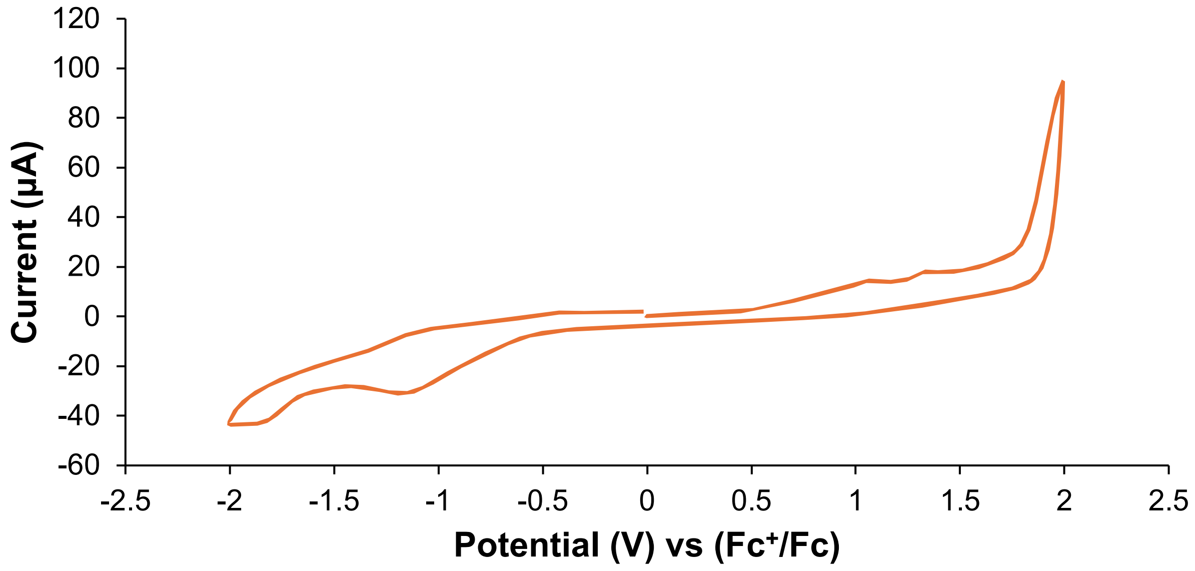

Figure SI-4b: Cyclic voltammogram of mixture of cyclohexyl isocyanide and $B(C_6F_5)_3$. Data was plotted according to the IUPAC convention. Voltammograms recorded using a reaction mixture of cyclohexyl isocyanide (16 mg, 0.15 mmol, 1.0 equiv, 0.015 M), tetra-*n*-butylammonium hexafluorophosphate (190 mg, 0.50 mmol, 3.4 equiv, 0.05 M) and $B(C_6F_5)_3$ (76 mg, 0.15 mmol, 1.0 equiv, 0.015 M) in MeCN (9.87 mL, 0.015 M). The plot was acquired using ν = 50 mV/s with a glassy carbon working electrode, a platinum wire counter electrode, and an $Ag/AgNO_3$ reference electrode. Acquisition was performed starting at 0 mV, with an initial rising segment to +2000 mV, followed by a falling segment to −2000 mV, and finally a rising segment to 0 mV. The resulting cyclic voltammograms were calibrated according to ferrocenium/ferrocene.

6.2. Solvent Addition Byproduct

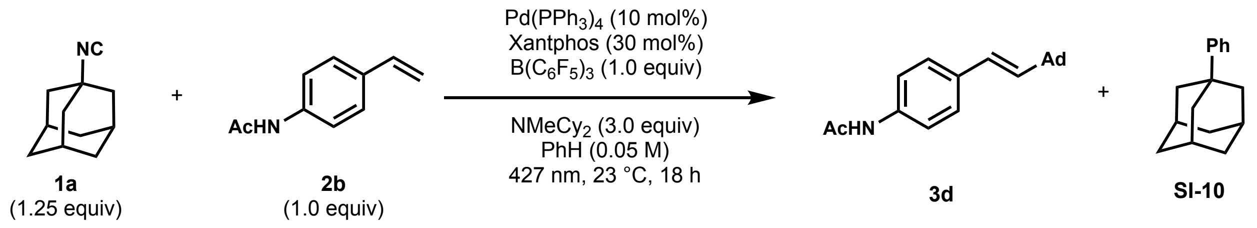

To determine the structure of a prevalent byproduct (**SI-10**), which is the resultant alkyl radical engaging with PhH, an experiment was set up according to **General Procedure C** using *N*-(4-vinylphenyl)acetamide (**2b**) (16 mg, 0.10 mmol, 1.0 equiv) and 1-isocyanoadamantane (**1a**) (20.2 mg, 0.125 mmol, 1.25 equiv). An analytically pure sample of **SI-10** was purified by silica preparative thin-layer chromatography (hexanes) and isolated as a colorless solid (**SI-5a**–**SI-5b**).

Note: A ^{1}H NMR yield could not be accurately determined from the crude reaction mixture.

All spectroscopic data for **SI-10** was consistent with that which was previously reported.[16]

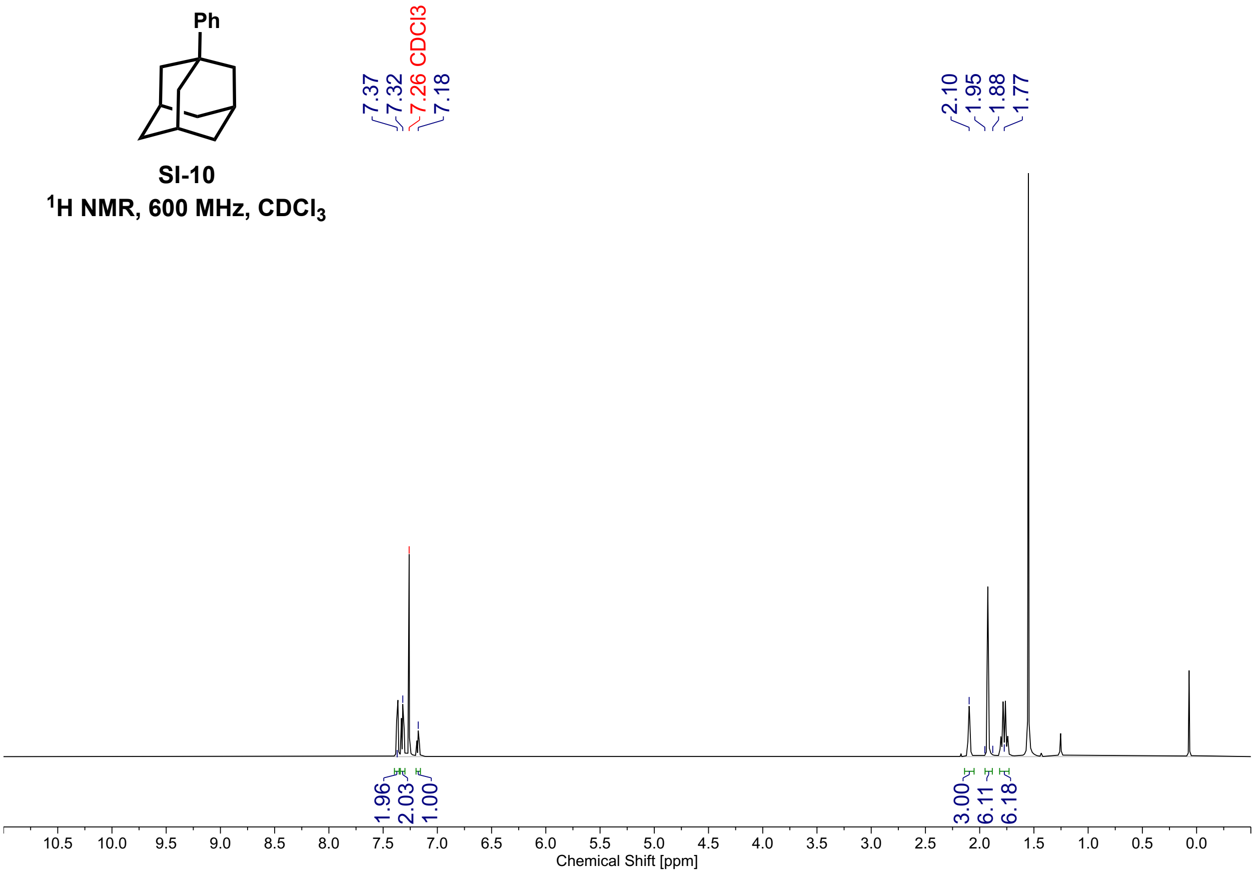

Figure SI-5a: ^{1}H NMR of byproduct **SI-10**.

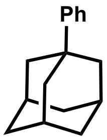

SI-10

^{13}C NMR, 151 MHz, $CDCl_3$

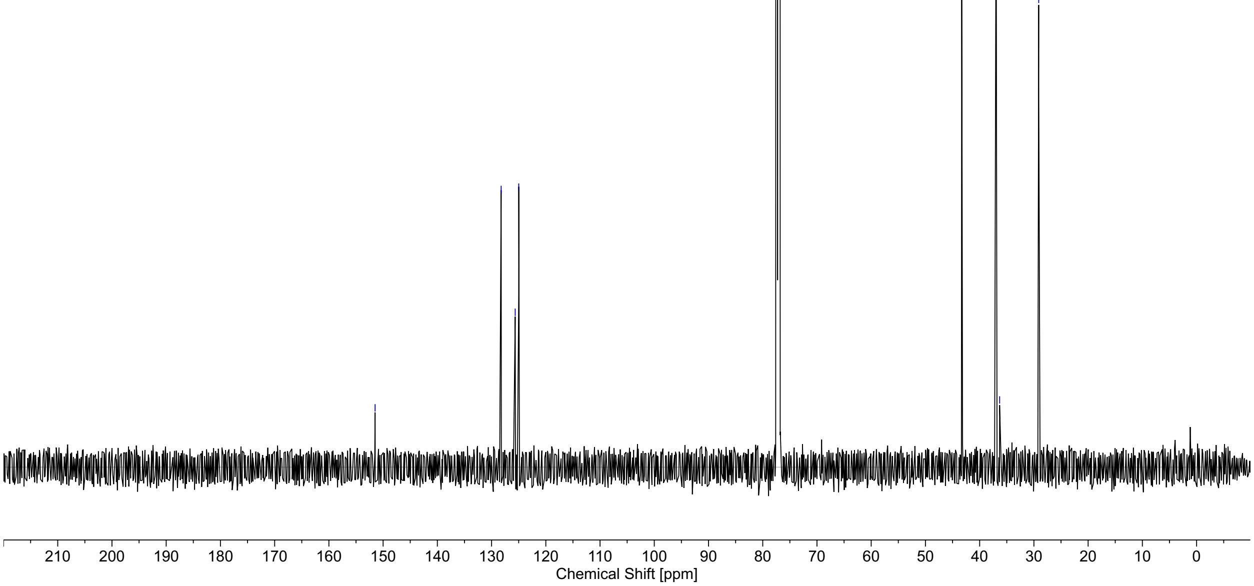

Figure SI-5b: ^{13}C NMR of byproduct **SI-10**.

6.3. Frustrated Lewis Pairs Experiment

Given the previously established ability of $B(C_6F_5)_3$ to form a radical pair with PR_3 or NR_3 (For P: R = Mes, *t*-Bu; For N: R = Ph or *p*-Me-Ph), we sought to exclude the possibility that a radical pair was the operative reducing species to produce the alkyl radical.[17]

<u>Procedure for Determining the Presence of Frustrated Lewis Pairs</u>

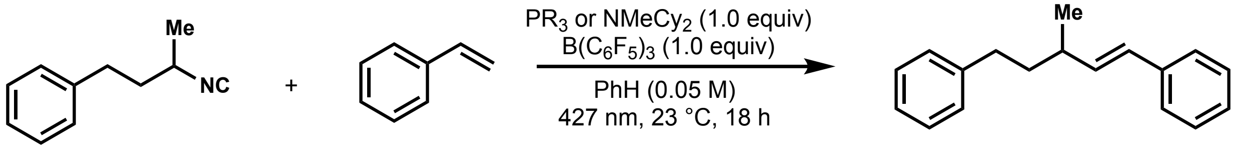

SI-1 (1.0 equiv) **2a** (1.0 equiv) **SI-2**

Inside a N_2-filled glovebox, a flame-dried reaction vial (VWR, catalog no. 66011-041) equipped with a magnetic stir bar (Chemglass Life Sciences, catalog no. CG-2003-160, 10 x 3 mm) was charged $B(C_6F_5)_3$ (26 mg, 0.05 mmol, 1.0 equiv) and, if using, PR_3 (0.05 mmol, 1.0 equiv). To the reaction mixture was added PhH (1.0 mL, 0.05 M) and, if using, $NMeCy_2$ (9.8 mg, 0.05 mmol, 1.0 equiv), and the solution was allowed to stir. After 10 min, to the resulting solution, (3-isocyanobutyl)benzene (**SI-1**) (8.0 mg, 0.05 mmol, 1.0 equiv) and styrene (**2a**) (5.2 mg, 0.05 mmol, 1.0 equiv) was added.

The reaction vessel was sealed with a PTFE-lined phenolic vial screw cap (Thermo Scientific, catalog no. 03-375-25A with 03-340-10G), removed from the glovebox, and placed in a photoreactor 3 cm away from two 45 W Kessil PR-160L 427 nm LEDs and one 75 mm fan. The reaction mixture was subjected to LED irradiation at 100% intensity with vigorous stirring. After 18h, the LEDs were turned off and the reaction mixture was filtered through a packed pad of Celite (ca. 20 mg) eluting with EtOAc (3 x 5 mL), and then concentrated *in vacuo* with the aid of a rotary evaporator. Yield was evaluated by ^{1}H NMR of the crude reaction mixture using 1,1,2,2-tetrachloroethane (TCE) (*ca.* 4.2 mg) as the internal standard.

Table 6.3.1. Frustrated Lewis Pairs Experimental Results

entry	PR_3 or $NMeCy_2$	Yield of SI-2
**1**	PPh_3	0%
**2**	$PMes_3$	0%
**3**	Tris(2,4,6-trimethoxyphenyl)phosphine	0%
**4**	Xantphos	0%
**5**	$NMeCy_2$	0%

6.4. UV-Vis Absorption Spectroscopy

<u>Preparation of samples for UV-Vis data collection:</u>

All solutions were prepared at 0.001 M in PhH. All samples were collected utilizing quartz cuvettes (Starna Cells, Inc., Cat. No.: 3-Q-10-GL14-S, 10 mm path). All samples were measured in absorbance mode on a Hitachi U3000 spectrophotometer, utilizing the UV Solutions (program no.: 1344331-15) software by Hitachi, with a wavelength range from 250–700 nm and a slit width of 2.0 mm.

In order to verify that the Pd(0) catalyst is the photoabsorbent species, UV-Vis spectra were acquired. Results demonstrate $Pd(PPh_3)_4$ shows absorbance between 400–500 nm, whereas $B(C_6F_5)_3$, styrene (**2a**), and 1-isocyanoadamantane (**1a**) lack absorbance in that wavelength region.

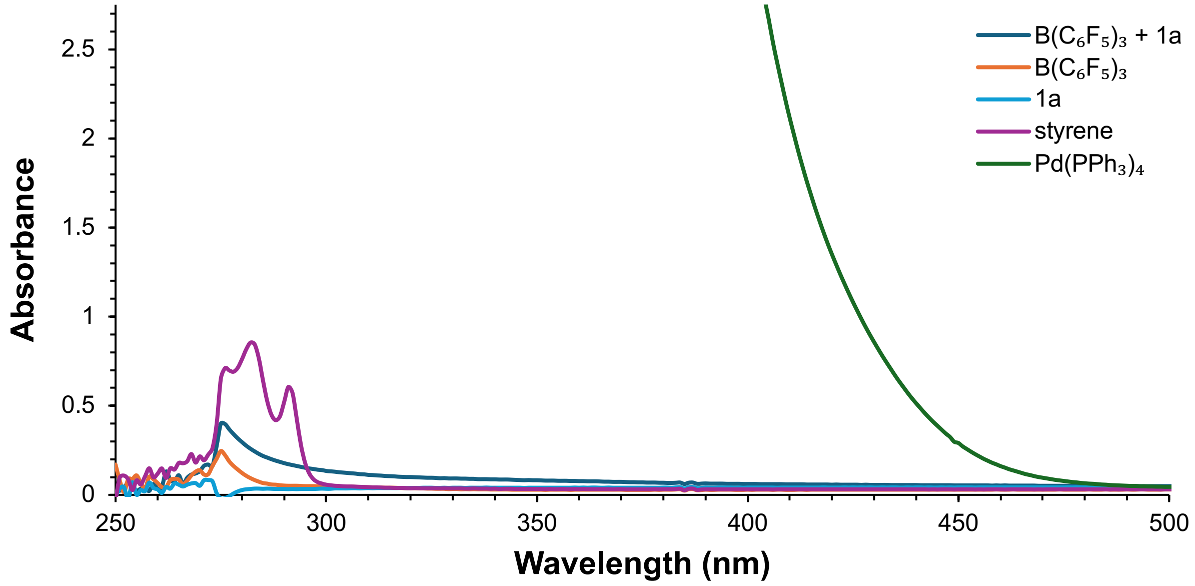

Figure SI-6: Absorption spectra of $Pd(PPh_3)_4$, $B(C_6F_5)_3$, styrene (**2a**), and 1-isocyanoadamantane (**1a**).

6.5. Stern-Volmer Fluorescence Quenching

Preparation of $B(C_6F_5)_3$ and isocyanoadamantane (**1a**) stock solutions:

Inside a N_2-filled glovebox, a 20 mL scintillation vial was charged with $B(C_6F_5)_3$ (51.2 mg, 0.10 mmol) and PhH (2.0 mL, 0.05 M). The resulting solution was shaken to ensure full homogeneity to yield a stock solution of $B(C_6F_5)_3$.

Inside a N_2-filled glovebox, a 20 mL scintillation vial was charged with isocyanoadamantane (**1a**) (16.1 mg, 0.10 mmol) and PhH (2.0 mL, 0.05 M). The resulting solution was shaken to ensure full homogeneity to yield a stock solution of isocyanoadamantane (**1a**).

Preparation of $Pd(PPh_3)_4$ stock solution:

Inside a N_2-filled glovebox, a 20 mL scintillation vial was charged with $Pd(PPh_3)_4$ (57.8 mg, 0.05 mmol) and PhH (2.0 mL, 0.025 M). The resulting solution was shaken to ensure full homogeneity to yield a stock solution of $Pd(PPh_3)_4$.

Preparation of quenching samples:

Into a 10 mm cuvette (VWR, Cat. No.: 414004-064, VWR Cell Fluoro Flat 10 mm), $Pd(PPh_3)_4$ stock solution (200 µL) and PhH (2.3 mL, final concentration: 0.002 M) were added. The resulting solution was shaken to ensure full homogeneity. Then, the cuvette was placed into a Varian Cary Eclipse Fluorescence Spectrophotometer. The fluorescence was measured utilizing fluorescence mode with an excitation wavelength at 427 nm, 10.0 nm excitation slit width, and 10.0 nm emission slit width. This process was repeated in its entirety with a modification of the amount of isocyanoadamantane (**1a**) solution added (25, 50, 75, 100, 125, 150, and 200 µL) to make 0.25, 0.5, 0.75, 1.0, 1.25, 1.5, and 2.0 equiv solutions of quencher, respectively. This process was also further repeated in its entirety with a modification of the amount of $B(C_6F_5)_3$ added (100 µL) to make a 1.0 equiv solution of quencher.

According to the Stern-Volmer equation, the ratio of catalyst emission intensity in the absence (I_0) and presence (I) of quencher, respectively, was plotted against the substrate concentration to obtain the Stern-Volmer fluorescence quenching constants. Based on the upward deviation observed, we concluded that there is likely a combination of static and dynamic quenching present in the reaction. We speculate that the static quenching may arise from coordination of the isonitrile (**1**) to the Pd^{0*} species, which could result in quenching of Pd^{0*} due to the formation of a new, non-fluorescent species. Based on the evidence that the addition of $B(C_6F_5)_3$ to the Pd^{0*} species also results in observation of fluorescence quenching, we hypothesize that there may be two operative mechanistic pathways for $C(sp^3)$–N bond cleavage. One possibility is that a single electron transfer occurs from the Pd^{0*} species to the isonitrile (**1**), which may be facilitated by coordination of $B(C_6F_5)_3$ to the isonitrile. Another proposed pathway is that there is an initial single electron transfer from the Pd^{0*} species to $B(C_6F_5)_3$, producing $[B(C_6F_5)_3]^{\cdot-}$, followed by reduction of the isonitrile (**1**). β-scission followed by either of these processes would then afford alkyl radical **II**.

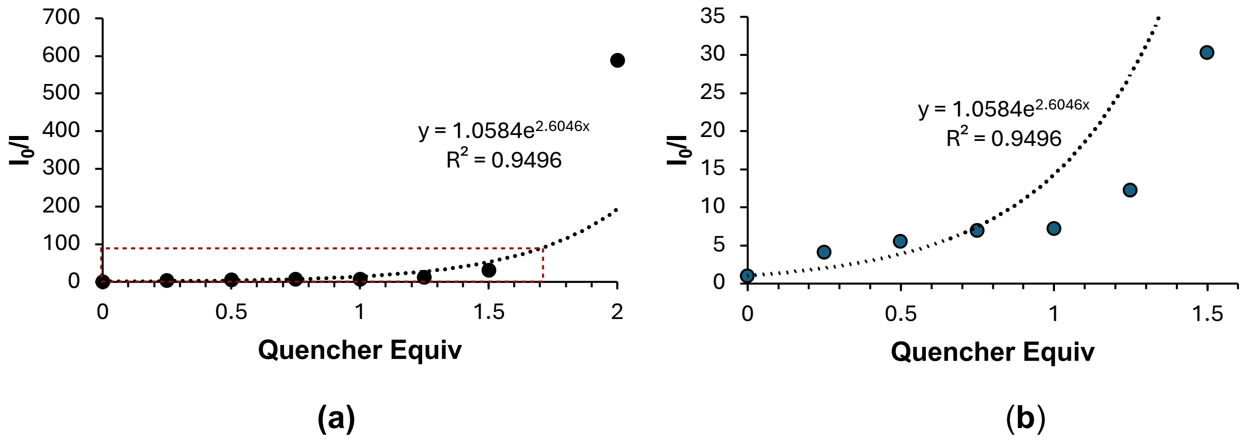

Figure SI-7: (**a**). Stern-Volmer plot for the fluorescence quenching experiment. (**b**). Zoomed Stern-Volmer plot for the fluorescence quenching experiment.

6.5.1. Emission Quenching of $Pd(PPh_3)_4$ by isocyanoadamantane (1a) and $B(C_6F_5)_3$.

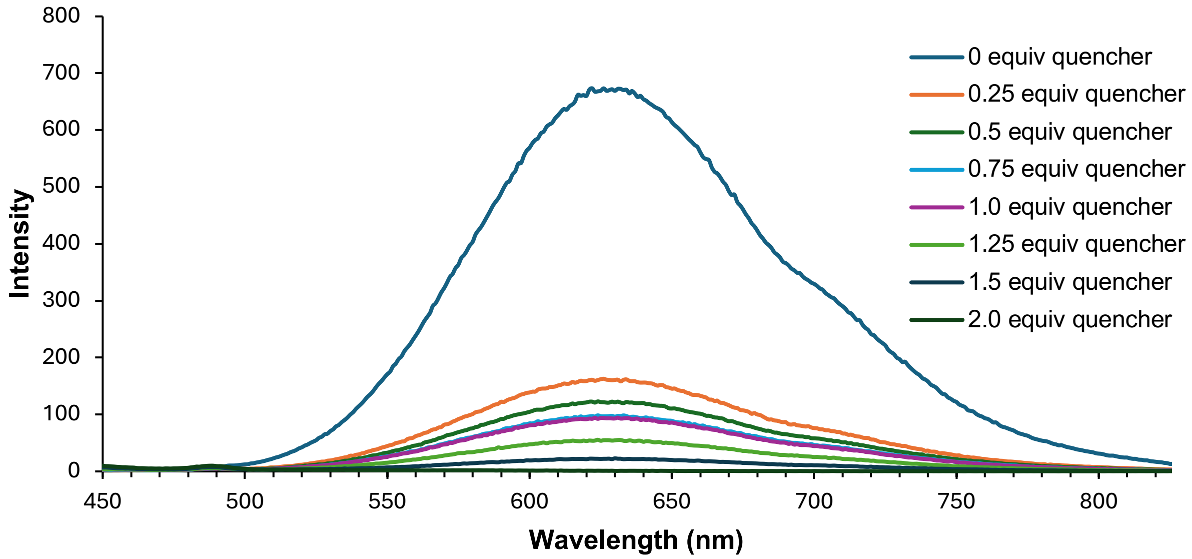

Figure SI-8a: Emission Quenching of $Pd(PPh_3)_4$ by isocyanoadamantane (**1a**) after irradiation at 427 nm at 25 °C.

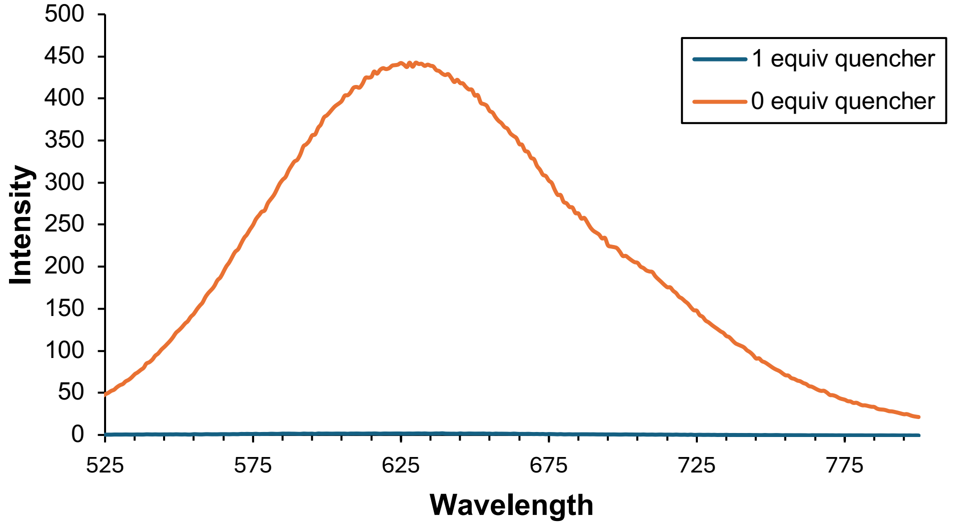

Figure SI-8b: Emission Quenching of $Pd(PPh_3)_4$ by $B(C_6F_5)_3$ after irradiation at 427 nm at 25 °C.

6.6. Fluorescence Spectra

Preparation of $Pd(PPh_3)_4$ stock solution:

Inside a N_2-filled glovebox, a 20 mL scintillation vial was charged with $Pd(PPh_3)_4$ (57.8 mg, 0.05 mmol) and PhH (2.0 mL, 0.025 M). The resulting solution was shaken to ensure full homogeneity to yield a stock solution of $Pd(PPh_3)_4$. A quartz cuvette was filled with the stock solution (200 µL) and PhH (2.3 mL, final concentration: 0.002 M) and placed into a Varian Cary Eclipse Fluorescence Spectrophotometer. The excitation fluorescence spectra were measured with an emission wavelength at 631 nm, 20 nm excitation slit width, and 20 nm emission slit width. The emission fluorescence spectra was measured with an excitation wavelength at 427 nm, 20 nm excitation slit width, and 20 nm emission slit width.

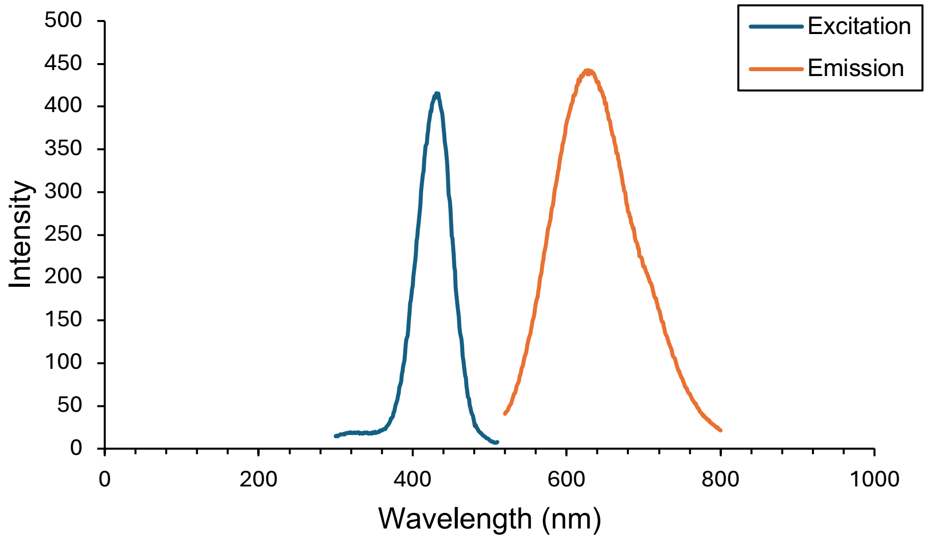

Figure SI-9: Excitation and emission spectra of $Pd(PPh_3)_4$.

6.7. General Procedure F: Preparation of ^{31}P Experiment

Preparation of $Pd(PPh_3)_4$ stock solution:

Inside a N_2-filled glovebox, a 20 mL scintillation vial was charged with $Pd(PPh_3)_4$ (46.2 mg, 40.0 μmol) and C_6D_6 (2.0 mL, 0.02 M). The resulting solution was shaken to ensure full homogeneity to yield a stock solution of $Pd(PPh_3)_4$.

Inside a N_2-filled glovebox, a 20 mL scintillation vial was charged with isocyanoadamantane (**1a**) (16.1 mg, 0.10 mmol) and C_6D_6 (2.0 mL, 0.05 M). The resulting solution was shaken to ensure full homogeneity to yield a stock solution of isocyanoadamantane (**1a**).

Preparation of isocyanoadamantane (**1a**) samples:

Inside a N_2 filled glovebox, $Pd(PPh_3)_4$ stock solution (300 μL) was added to an oven-dried NMR tube. To the reaction mixture was added C_6D_6 (300 μL, final concentration: 0.01 M). The NMR tube was shaken to ensure full homogeneity. Next, the NMR tube was sealed with a cap, removed from the glovebox, and subjected to ^{31}P analysis. This process was repeated in its entirety with a modification of the amount of isocyanoadamantane (**1a**) solution added (60, 120, 180 μL) to make 0.5, 1.0, 1.5 equiv solutions of isocyanoadamantane (**1a**), respectively.

The results demonstrate no change in the ^{31}P spectrum of $Pd(PPh_3)_4$, regardless of the concentration of isocyanoadamantane (**1a**). This experiment therefore suggests ligand exchange of $Pd(PPh_3)_4$ with the isonitrile is unlikely under non-photoinduced conditions.

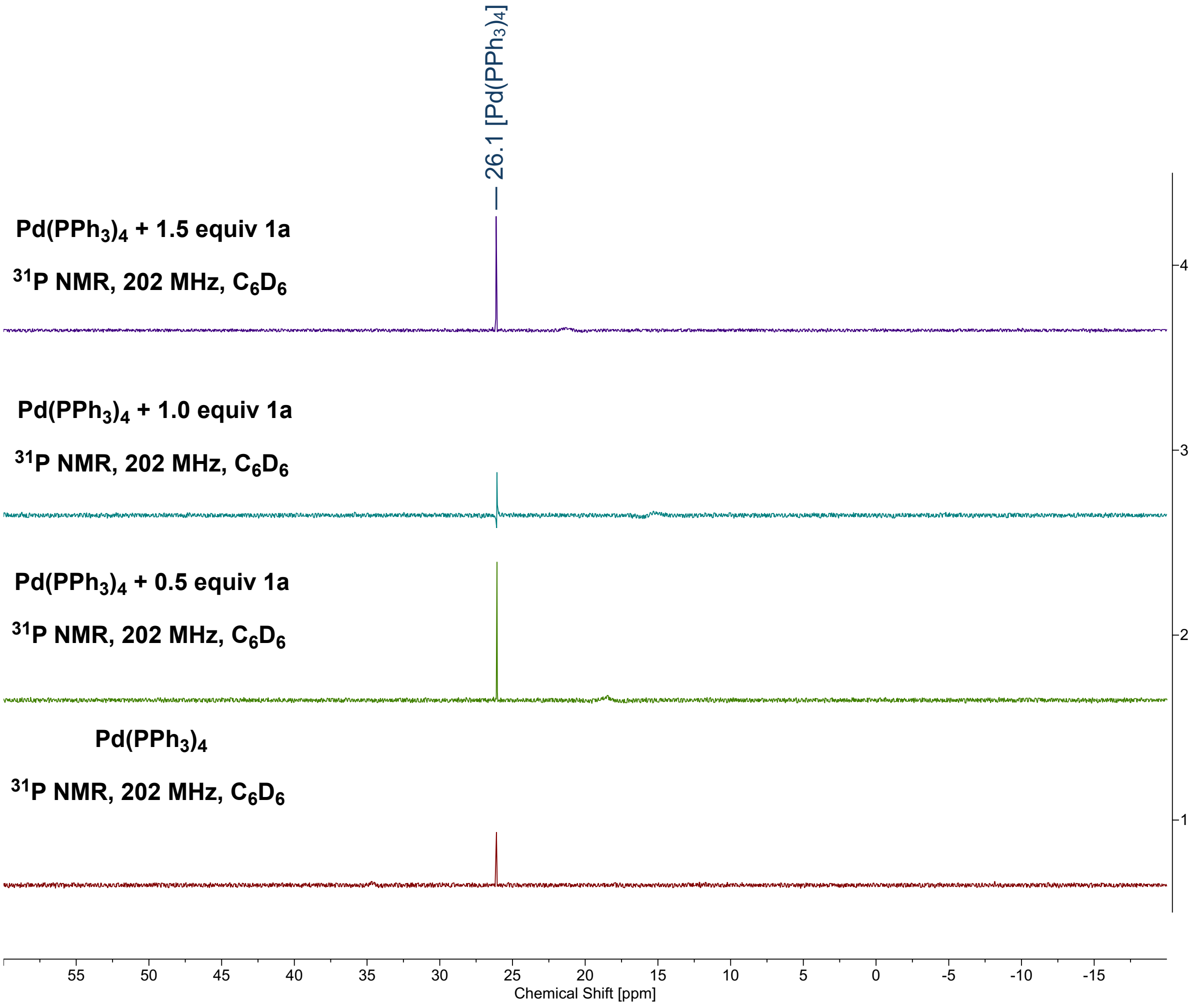

Figure SI-10: Results of ^{31}P experiment.

6.7.1. Photoirradiated ^{31}P Experiment

A sample was prepared according to **General Procedure F** containing isocyanoadamantane (**1a**) (1.5 equiv) and $Pd(PPh_3)_4$ (1 equiv). The sample was subjected to LED irradiation using two 45 W Kessil PR-160L 427 nm LEDs at 100% intensity placed at a distance of 3 cm from the NMR tube. Subsequently, the sample was analyzed by ^{31}P analysis after a period of 5, 10, 20, and 30 mins.

The results indicate that after 30 min of light irradiation, a new ^{31}P peak appears at δ = 24.0 ppm. This signal aligned with $Pd(CN)_2(PPh_3)_2$, which is in accordance with previously reported values.[18]

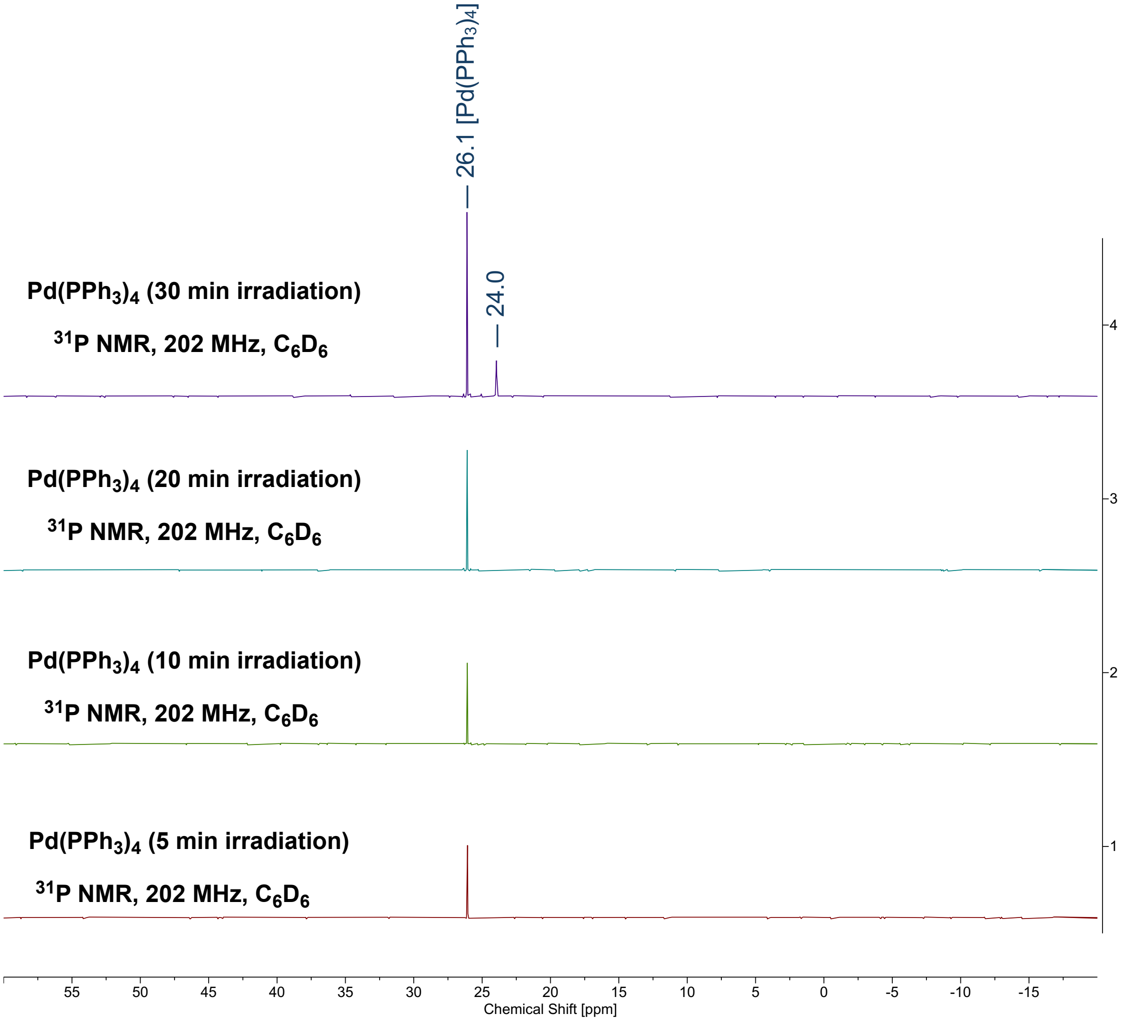

Figure SI-11: Results of ^{31}P irradiation experiment.

6.8. ^{11}B NMR/ ^{19}F NMR Studies

Preparation of crude reaction mixture:

This experiment was prepared according to **General Procedure C** using styrene (**2a**) (10 mg, 0.1 mmol), 1-isocyanoadamantane (**1a**) (20.2 mg, 0.125 mmol, 1.25 equiv), and C_6D_6 (1.00 mL, 0.05 M). After 18 h, 600 μL of the solution was transferred via syringe into an NMR tube under N_2 atmosphere, sealed, removed from the glovebox, and subjected to^{11}B NMR and ^{19}F NMR analysis.

General Procedure G: Preparation of ^{11}B NMR/ ^{19}F NMR Studies

Inside a N_2 filled glovebox, to an oven-dried reaction vial (VWR, catalog no. 66011-041) equipped with a magnetic stir bar was added $B(C_6F_5)_3$ (15 mg, 0.030 mmol, 1.0 equiv), additive (0.030 mmol, 1.0 equiv) and C_6D_6 (0.6 mL, 0.05 M). The reaction mixture was allowed to stir for 5 min at room temperature. Next, the solution was transferred via a syringe into an NMR tube, sealed, removed from the glovebox, and subjected to ^{11}B NMR and ^{19}F NMR analysis.

The ^{11}B spectra and ^{19}F spectra obtained suggest that $B(C_6F_5)_3$ may form a Lewis base adduct with either Xantphos or $NMeCy_2$ during the course of the reaction. Moreover, the results suggest that $B(C_6F_5)_3$ may form a complex with the isonitrile (**1**), which supports the hypothesis that $B(C_6F_5)_3$ may facilitate a single electron reduction of the isonitrile (**1**).

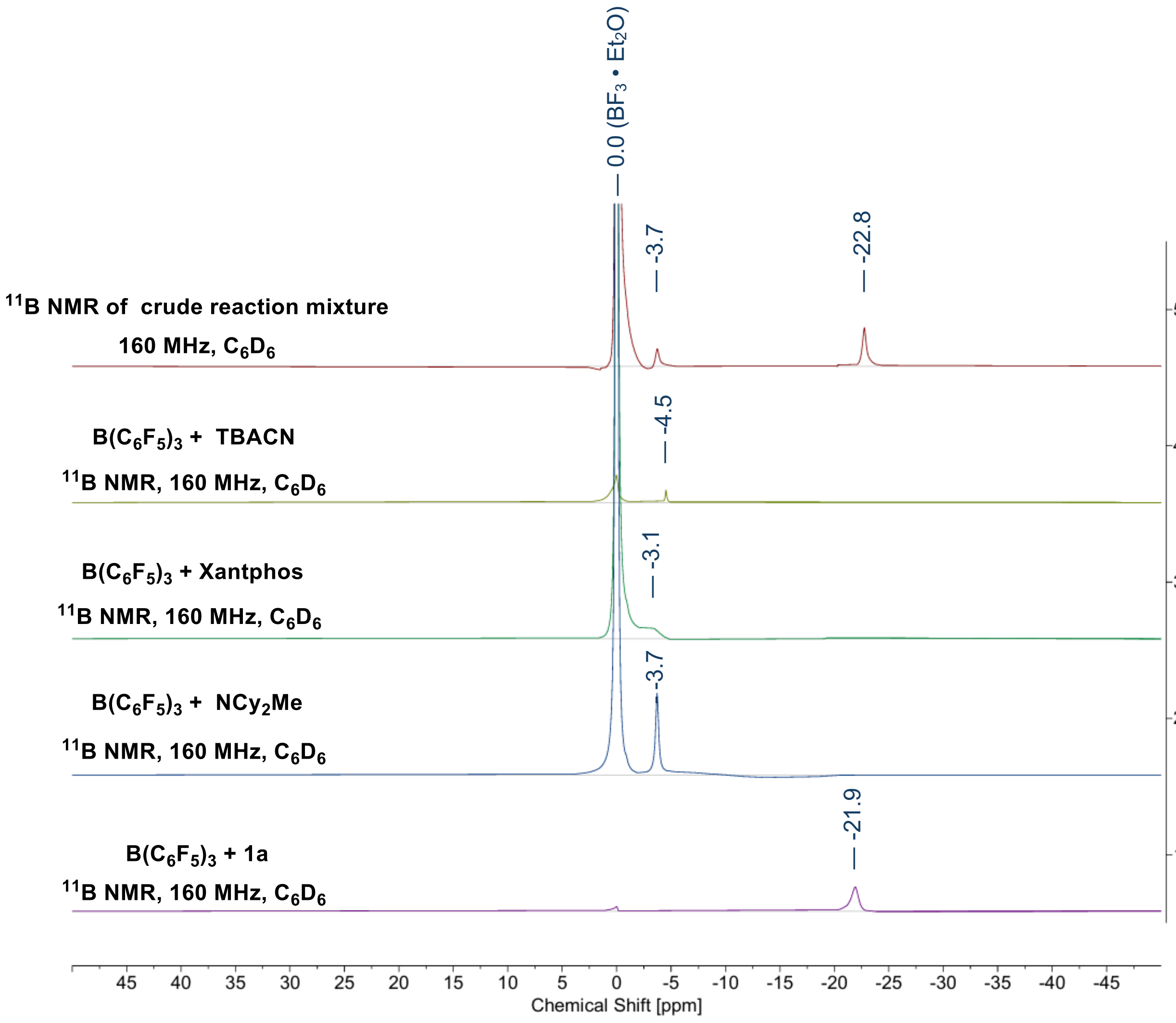

Figure SI-12a: ^{11}B NMR of crude reaction mixture and the mixture of $B(C_6F_5)_3$ with additives.

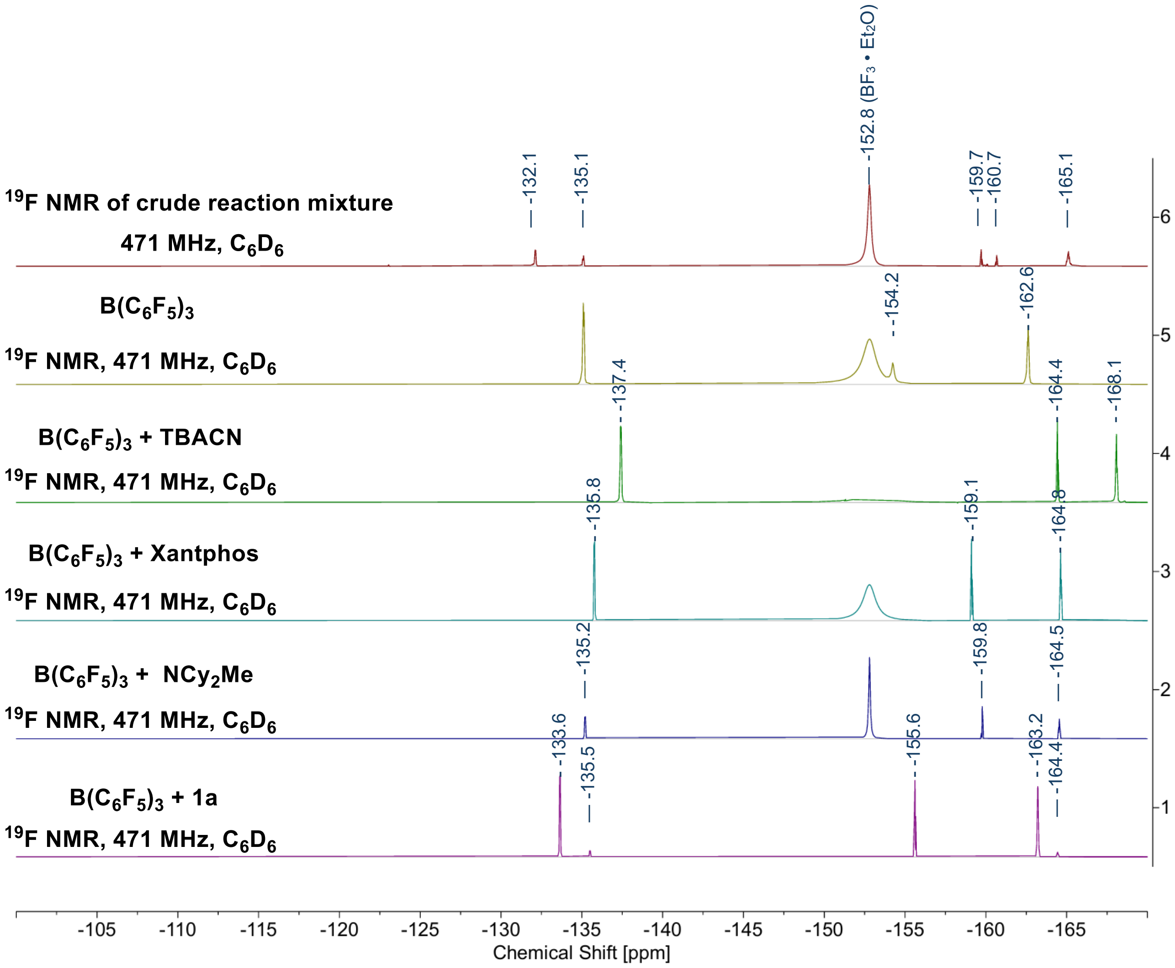

Figure SI-12b: ^{11}F NMR of crude reaction mixture, $B(C_6F_5)_3$ and the mixture of $B(C_6F_5)_3$ with additives.

6.9. TEMPO Trapping Experiment

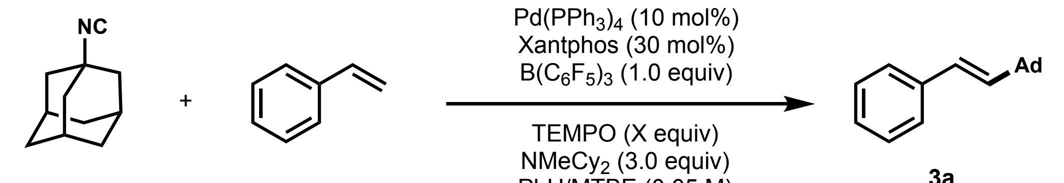

1a (1.25 equiv) **2a** (1.0 equiv) **3a**

Prepared according to modified **General Procedure C** using 1-isocyanoadamantane (**1a**) (20.0 mg, 0.125 mmol, 1.25 equiv) and 2,2,6,6-tetramethyl-1-piperidinyloxy (TEMPO). Yield of **3a** was determined by ^{1}H NMR spectroscopy of the crude reaction mixture using CH_2Br_2 as the internal standard.

Table 6.9.1. TEMPO Trapping Results

entry	TEMPO (equiv)	yield
**1**	0.1	37%
**2**	1.0	7%
**3**	2.0	0%

7. Substrate Scope Limitations

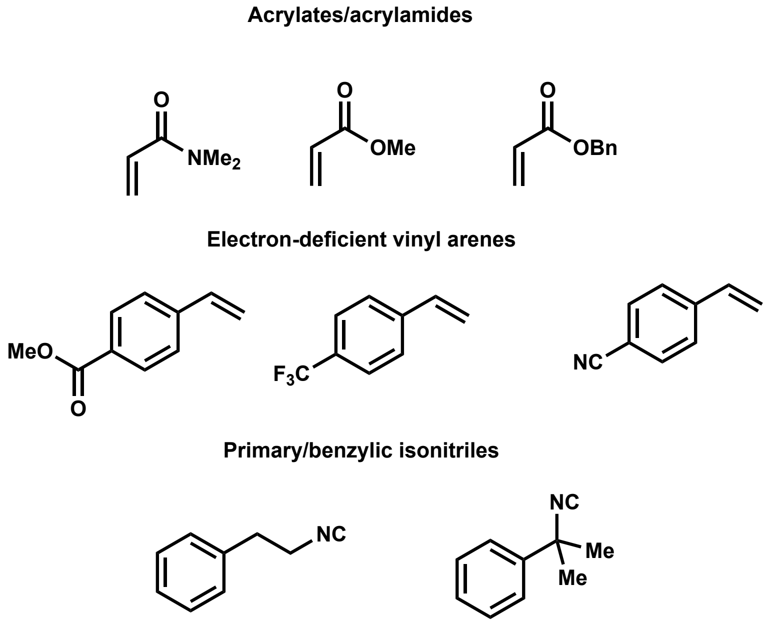

Figure SI-13: Substrate scope limitations of photocatalyzed Mizoroki-Heck cross-coupling.[a,b]

[a]All reactions performed on 0.05 mmol scale with respect to vinyl arene. Yields determined by ^{1}H NMR spectroscopy of the crude reaction mixtures utilizing 1,1,2,2-tetrachloroethane (TCE) or CH_2Br_2 as the internal standard.

[b]A yield of <5% was observed for all of the above substrates.

8. Associated Analytical Data

8.1. Associated NMR Spectra

8.1.1. NMR Spectra of Starting Materials

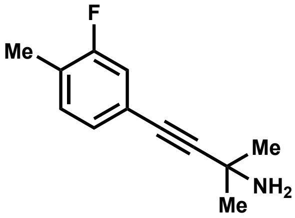

SI-3

^{1}H NMR, 600 MHz, $CDCl_3$

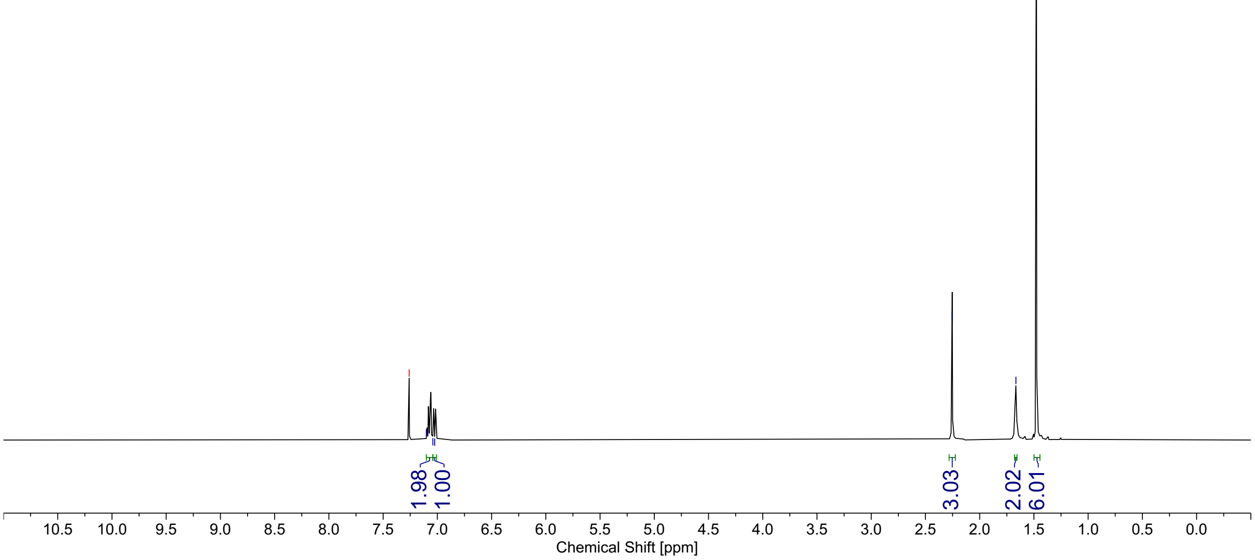

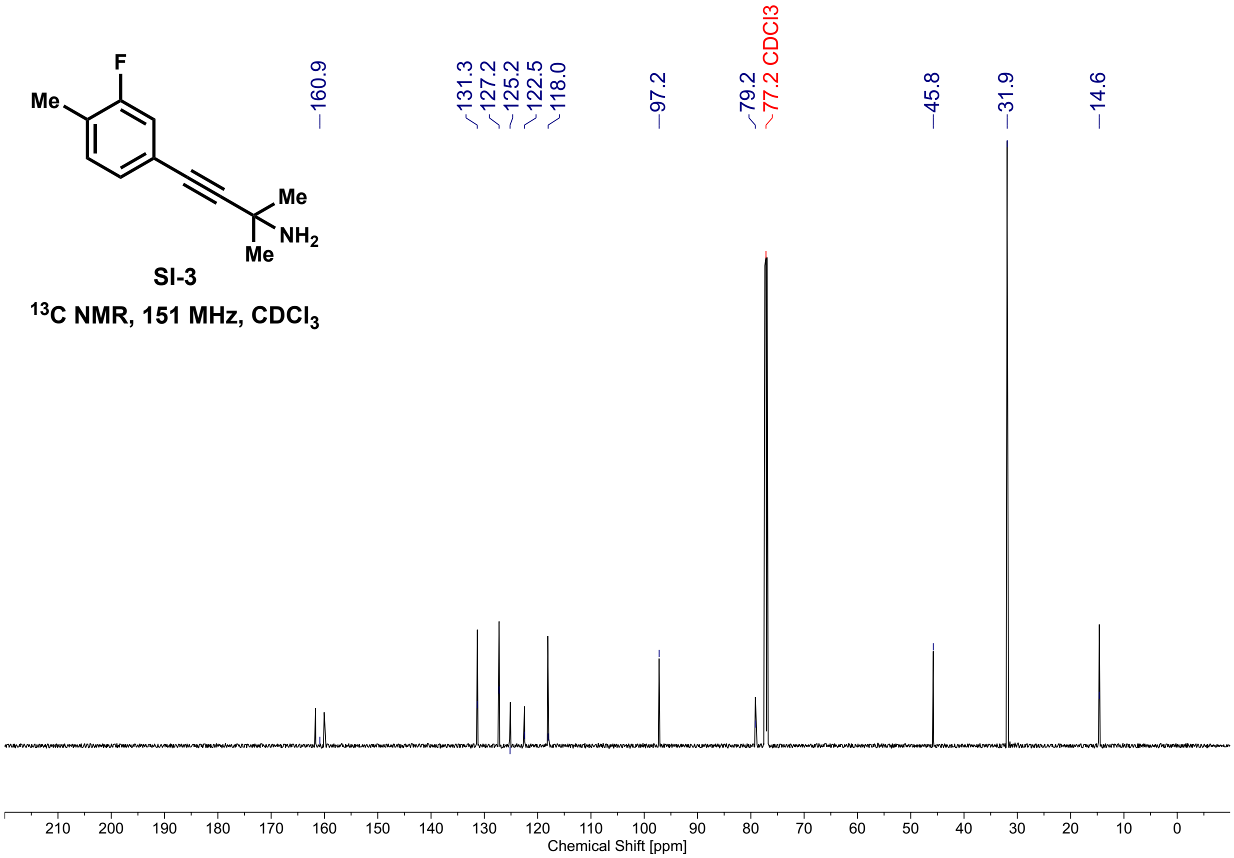
F
Me
Me
NH2
Me
SI-3
13C NMR, 151 MHz, CDCl3
160.9
131.3
127.2
125.2
122.5
118.0
97.2
79.2
77.2 CDCl3
45.8
31.9
14.6
Chemical Shift [ppm]

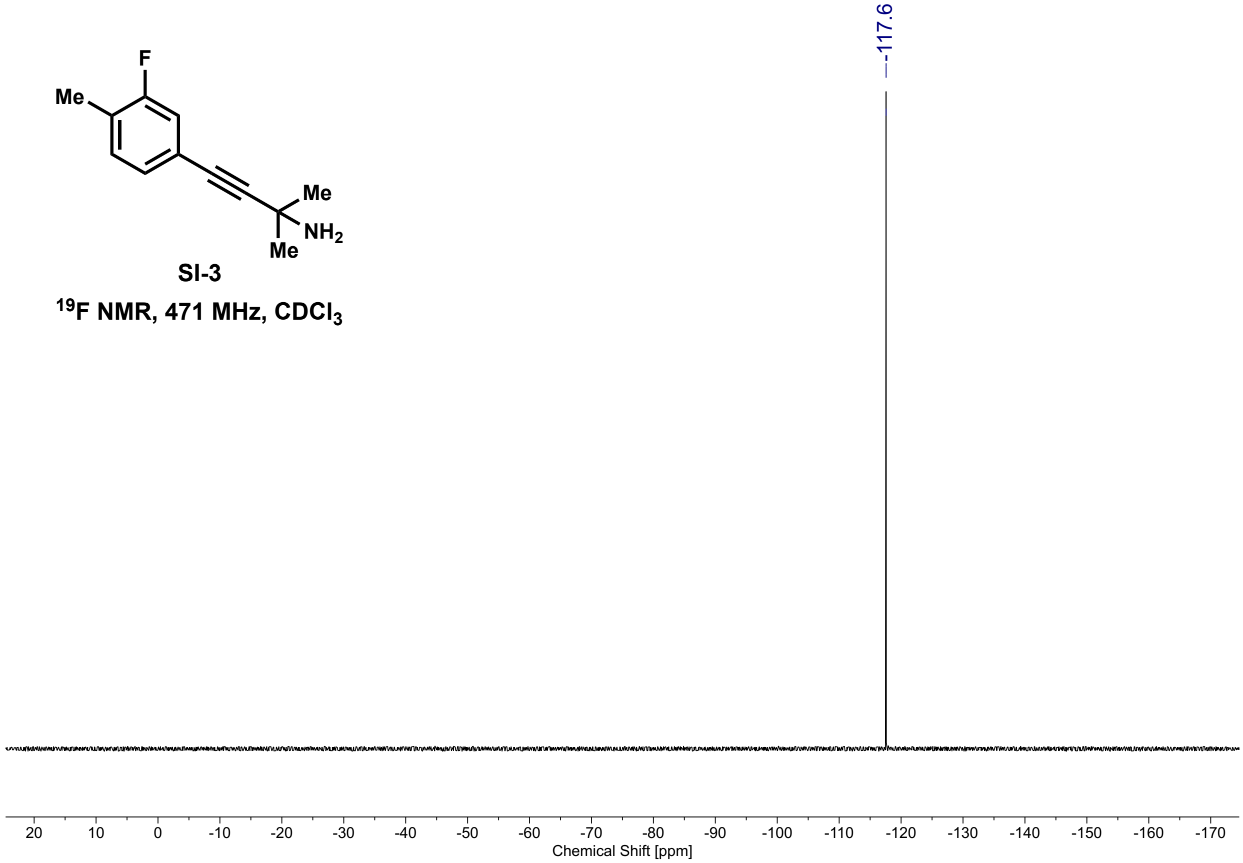
F
Me
Me
NH2
Me
SI-3
19F NMR, 471 MHz, CDCl3
-117.6
Chemical Shift [ppm]

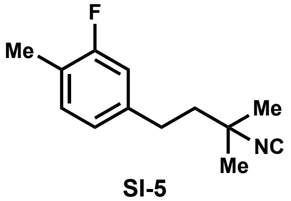

SI-5

^{1}H NMR, 600 MHz, $CDCl_3$

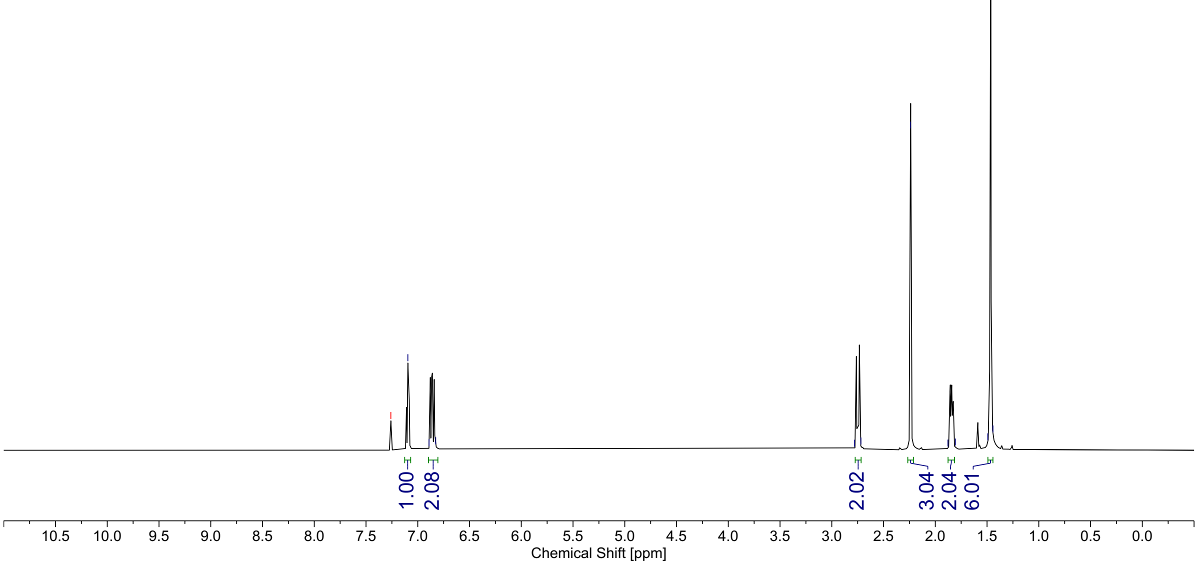

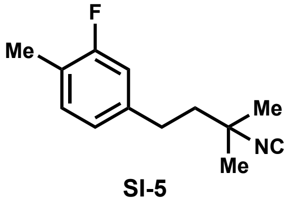

SI-5

^{13}C NMR, 151 MHz, $CDCl_3$

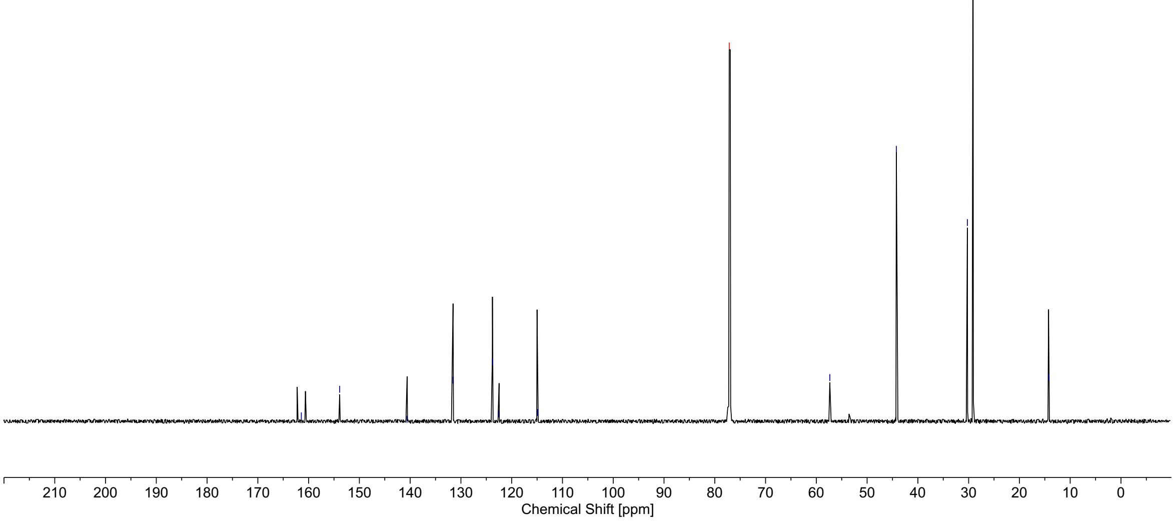

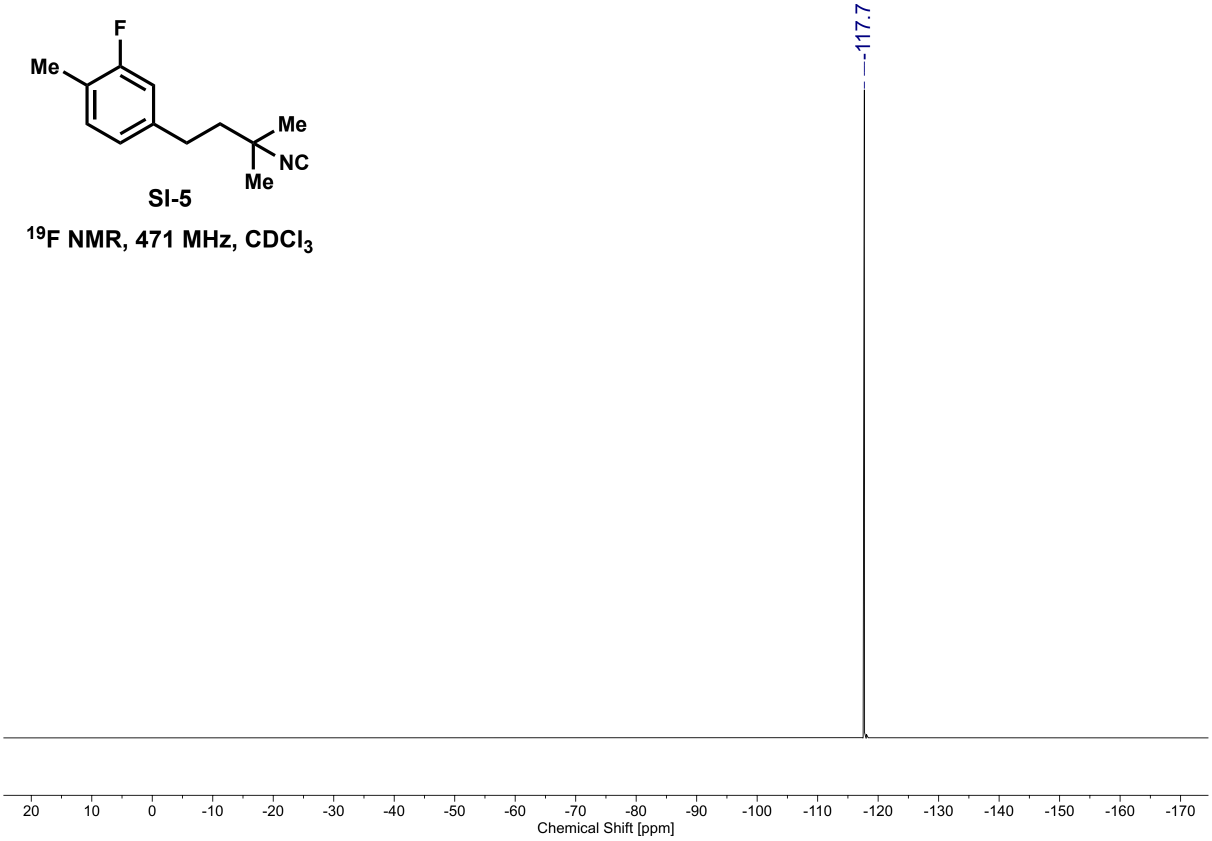

F
Me
Me
NC
Me
SI-5
19F NMR, 471 MHz, CDCl3
-117.7
20 10 0 -10 -20 -30 -40 -50 -60 -70 -80 -90 -100 -110 -120 -130 -140 -150 -160 -170
Chemical Shift [ppm]

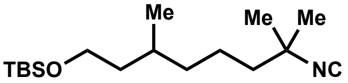

SI-7

^{1}H NMR, 600 MHz, $CDCl_3$

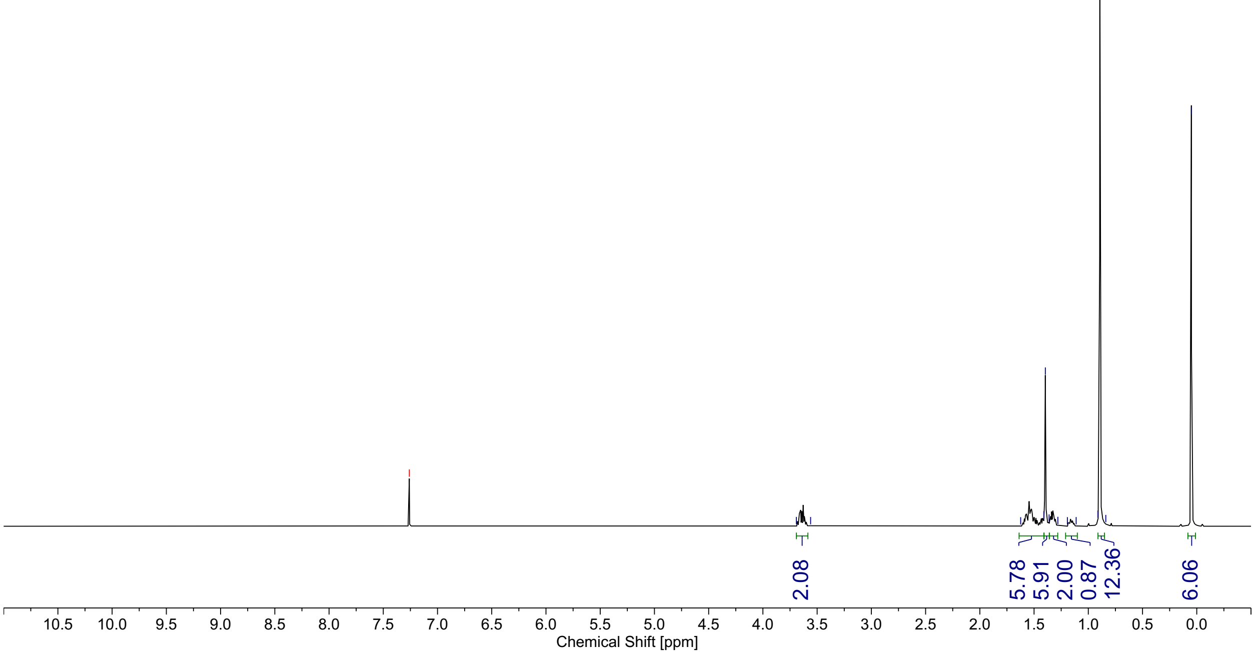

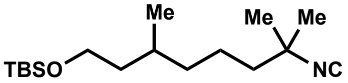

SI-7

^{13}C NMR, 151 MHz, $CDCl_3$

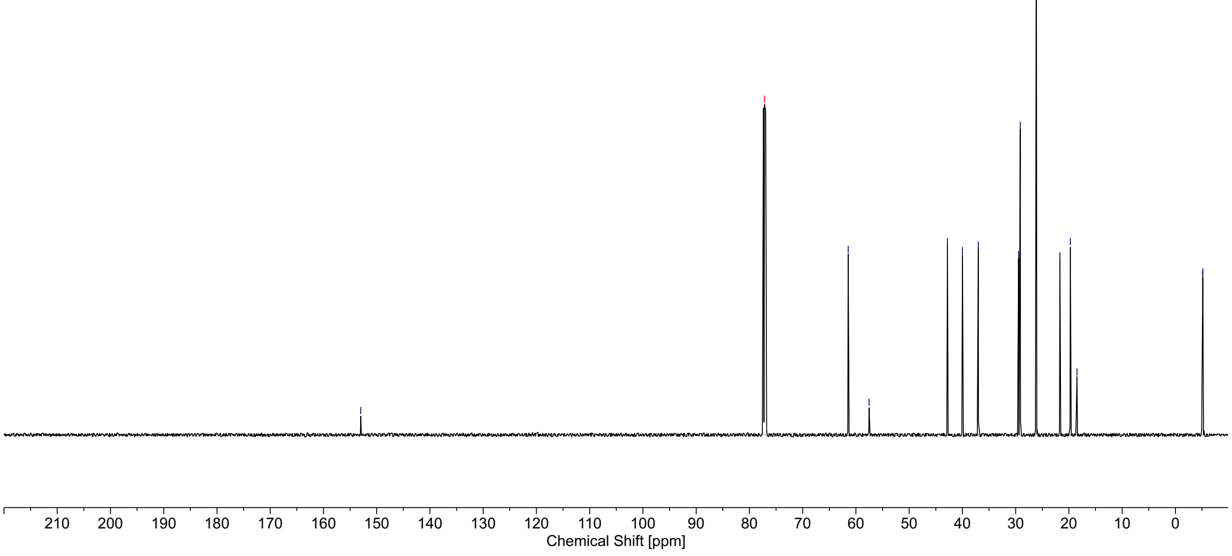

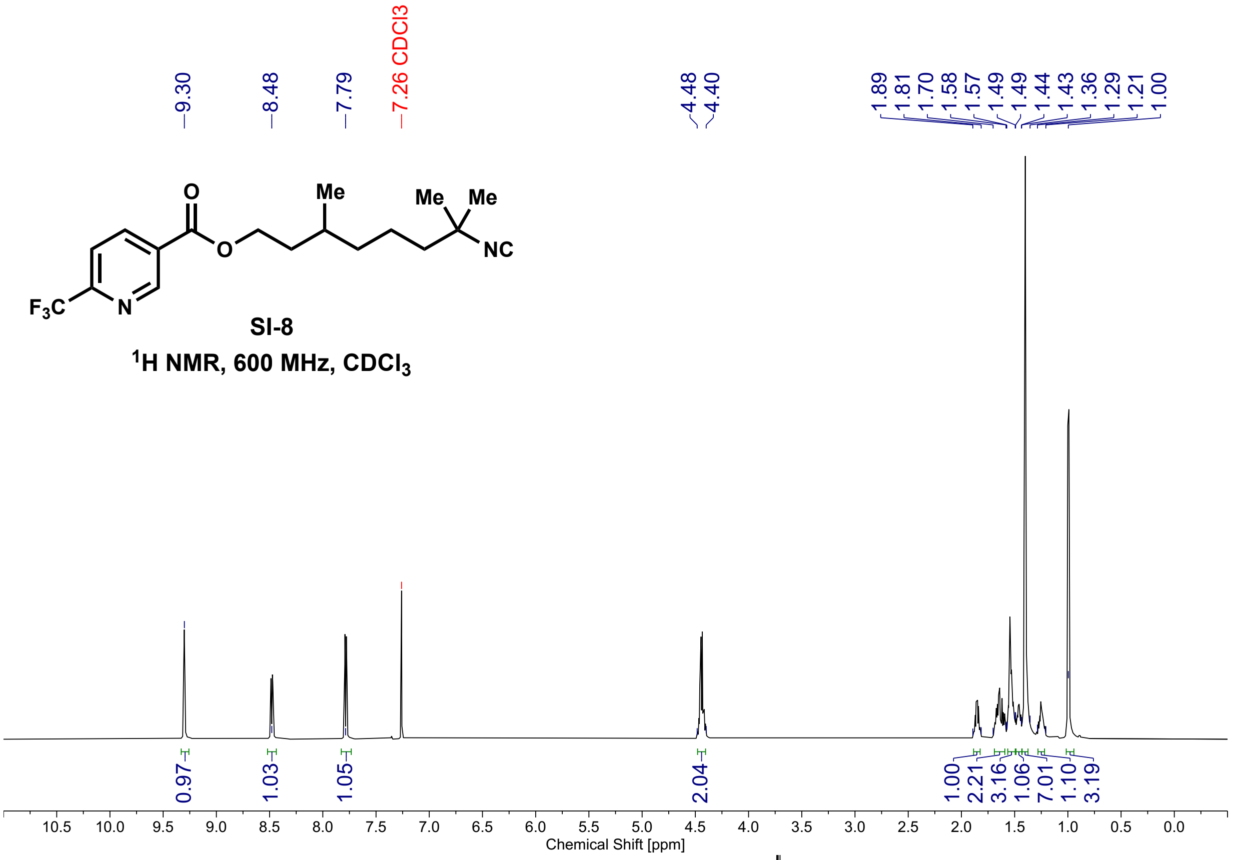

SI-8

^{1}H NMR, 600 MHz, $CDCl_3$

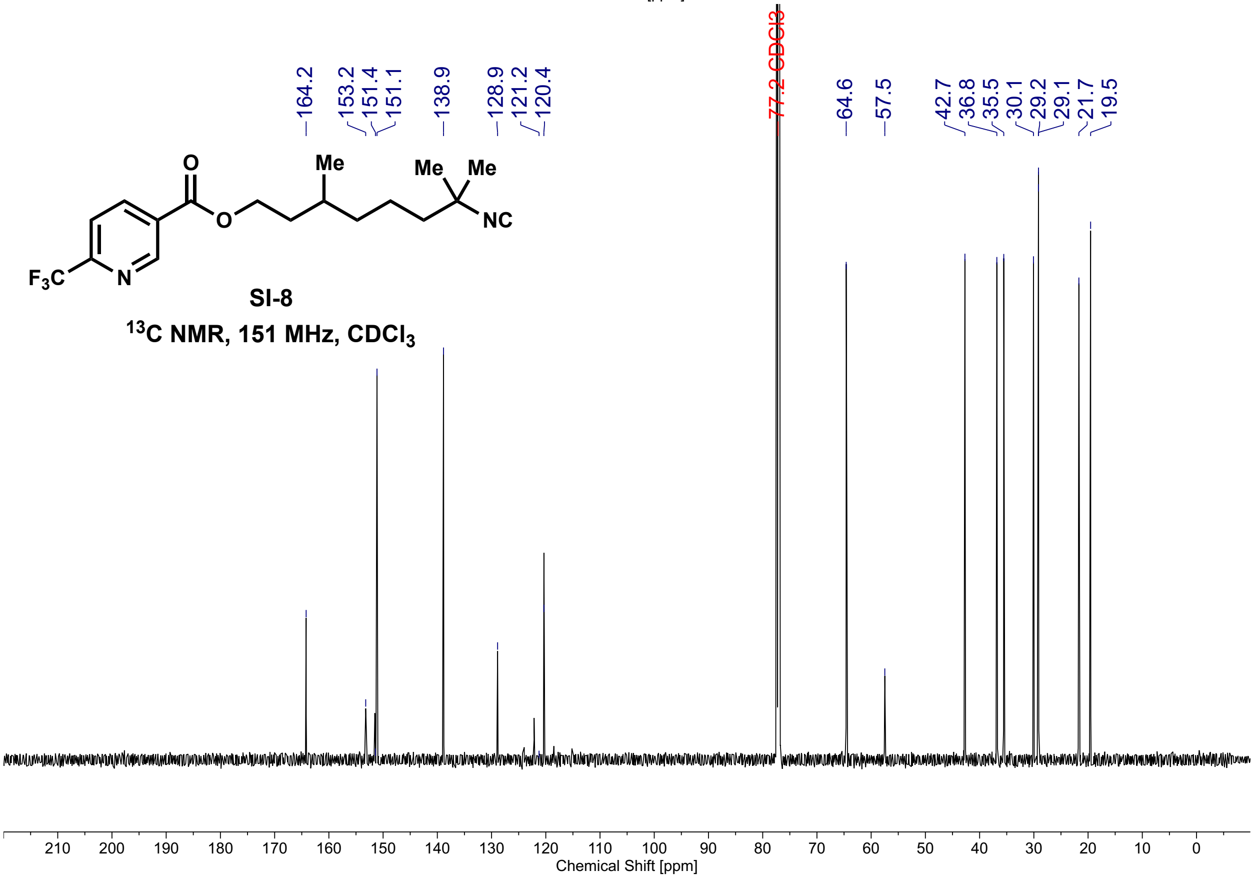

SI-8

^{13}C NMR, 151 MHz, $CDCl_3$

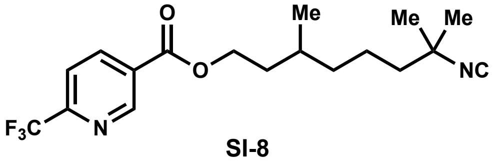

SI-8

^{19}F NMR, 471 MHz, $CDCl_3$

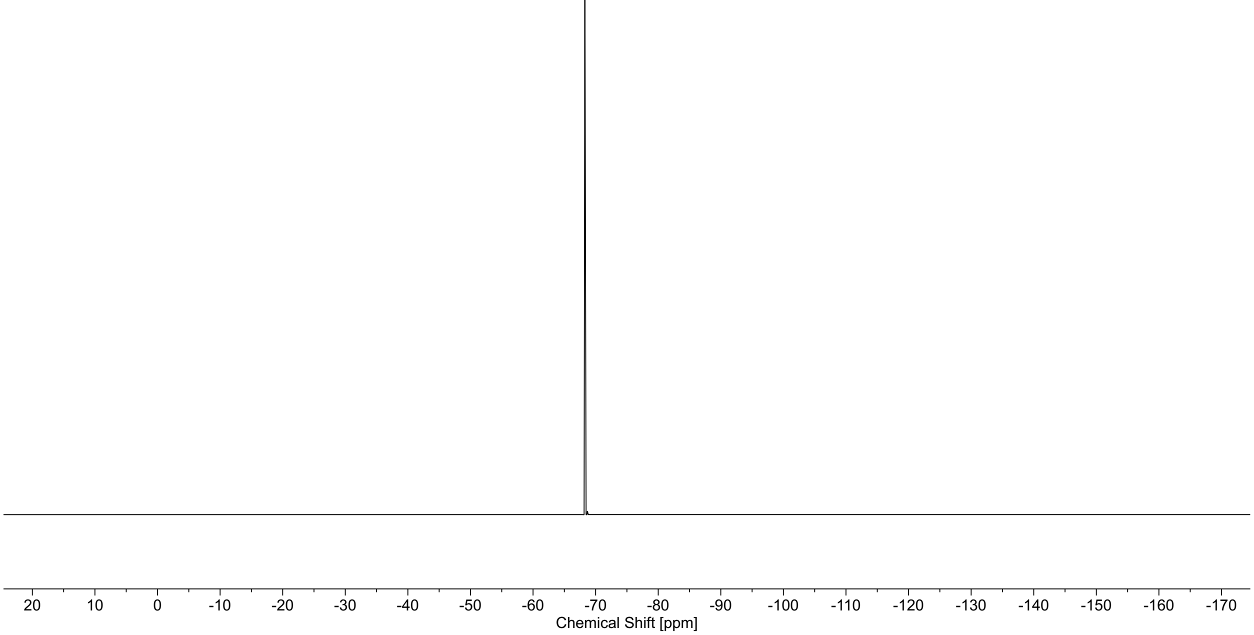

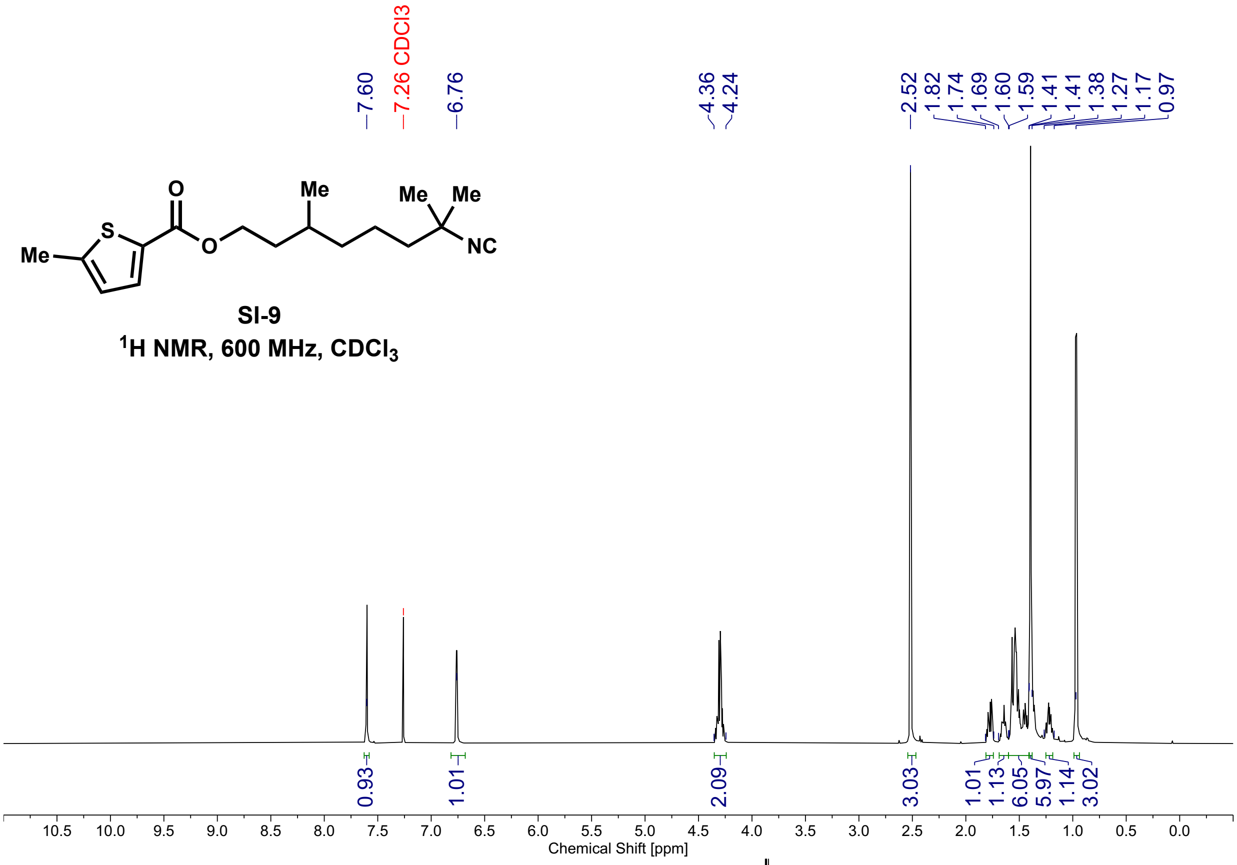
7.60
7.26 CDCl3
6.76
4.36
4.24
2.52
1.82
1.74
1.69
1.60
1.59
1.41
1.41
1.38
1.27
1.17
0.97
O
S
O
Me
Me
Me
Me
NC
SI-9
1H NMR, 600 MHz, CDCl3
0.93
1.01
2.09
3.03
1.01
1.13
6.05
5.97
1.14
3.02
10.5
10.0
9.5
9.0
8.5
8.0
7.5
7.0
6.5
6.0
5.5
5.0
4.5
4.0
3.5
3.0
2.5
2.0
1.5
1.0
0.5
0.0
Chemical Shift [ppm]

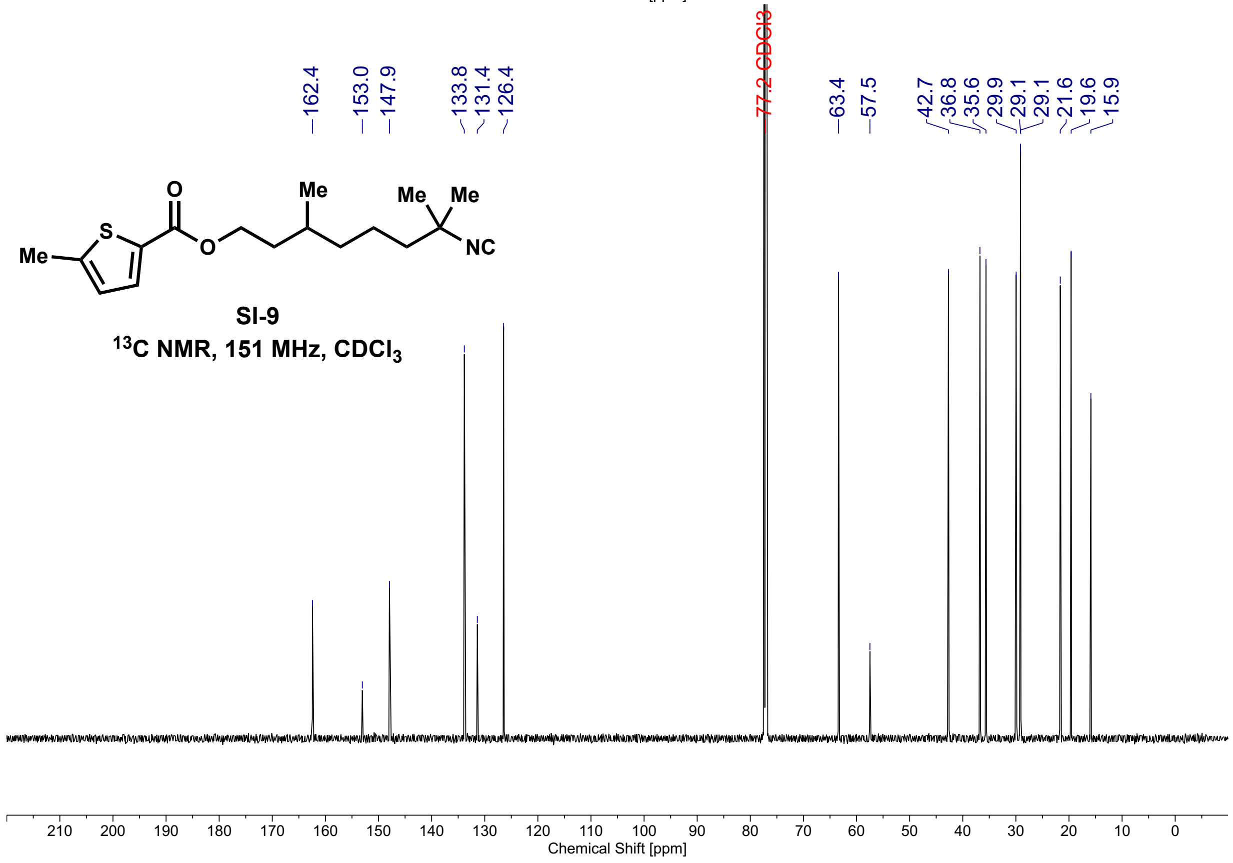
162.4
153.0
147.9
133.8
131.4
126.4
77.2 CDCl3
63.4
57.5
42.7
36.8
35.6
29.9
29.1
29.1
21.6
19.6
15.9
O
S
O
Me
Me
Me
Me
NC
SI-9
13C NMR, 151 MHz, CDCl3
210
200
190
180
170
160
150
140
130
120
110
100
90
80
70
60
50
40
30
20
10
0
Chemical Shift [ppm]

8.1.2. NMR Spectra of Products

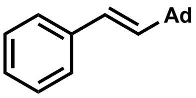

3a

^{1}H NMR, 600 MHz, $CDCl_3$

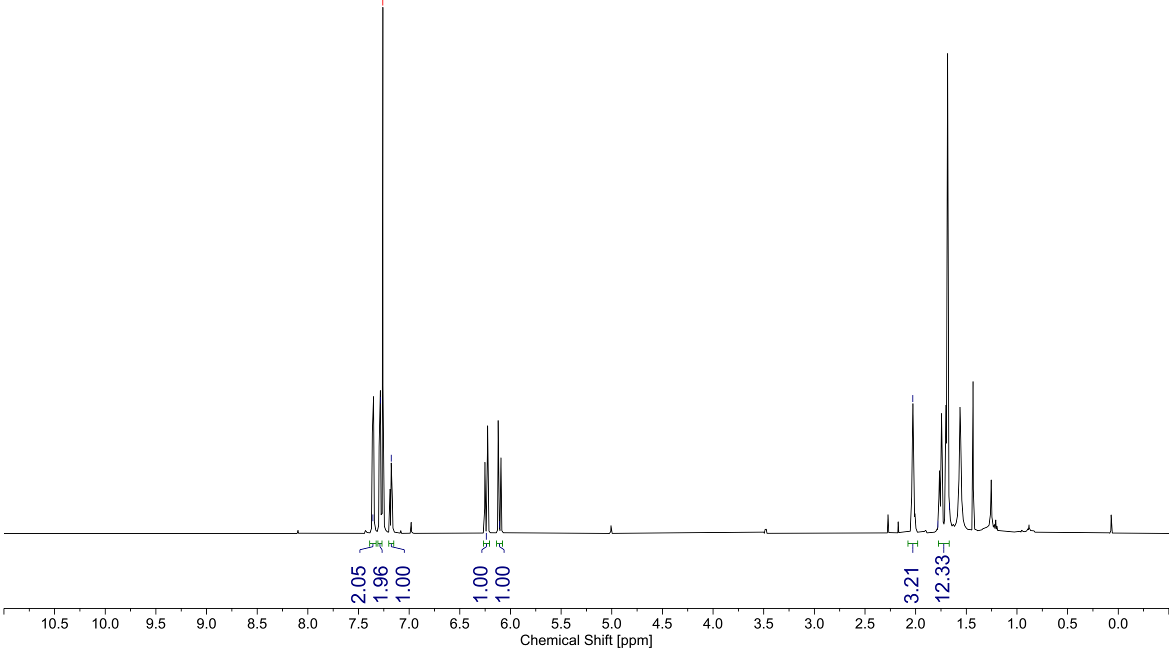

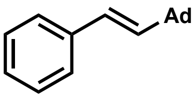

3a

^{13}C NMR, 151 MHz, $CDCl_3$

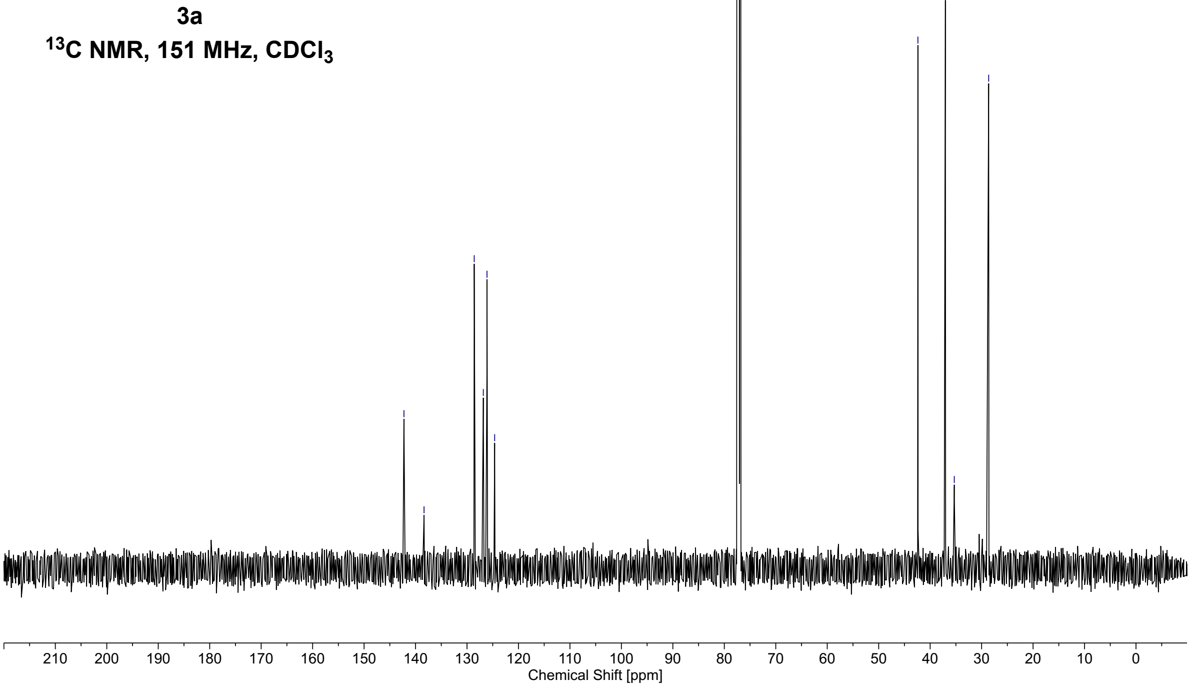

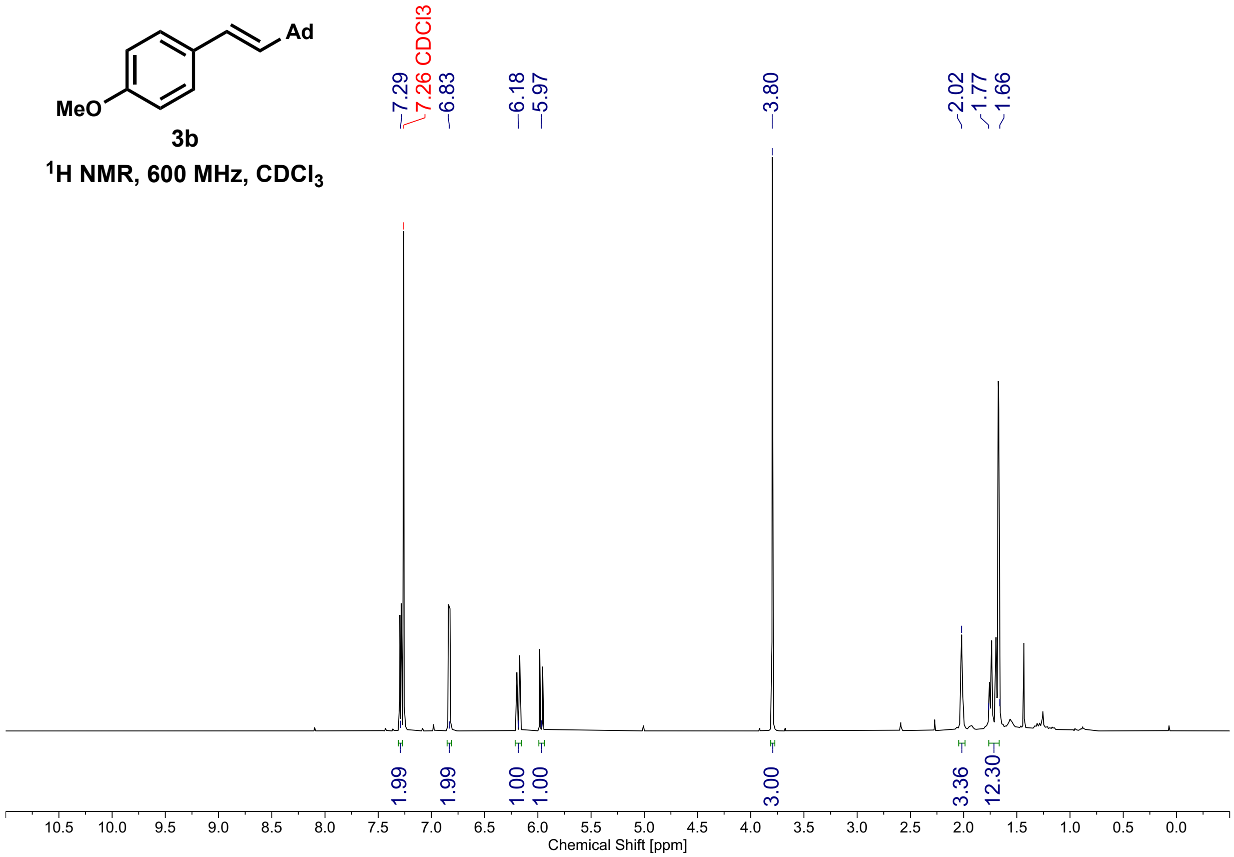

3b

^{1}H NMR, 600 MHz, $CDCl_3$

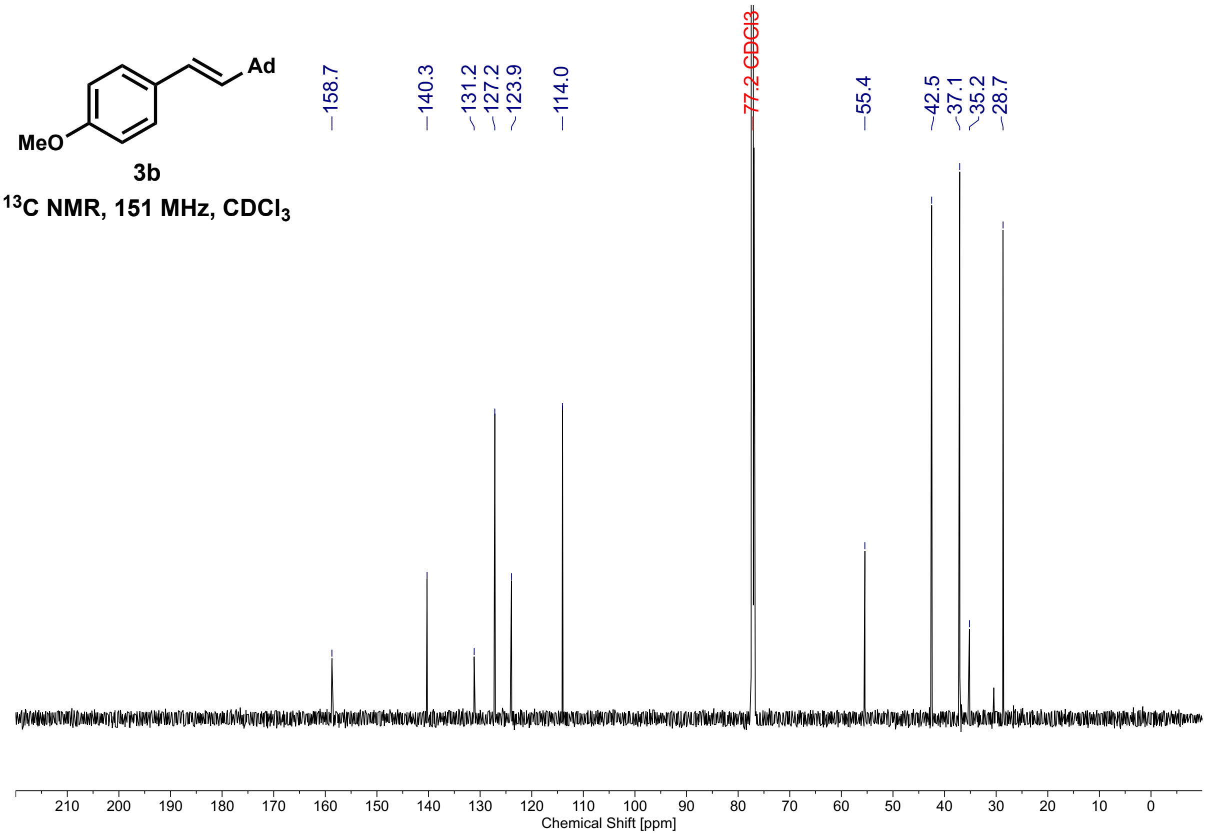

3b

^{13}C NMR, 151 MHz, $CDCl_3$

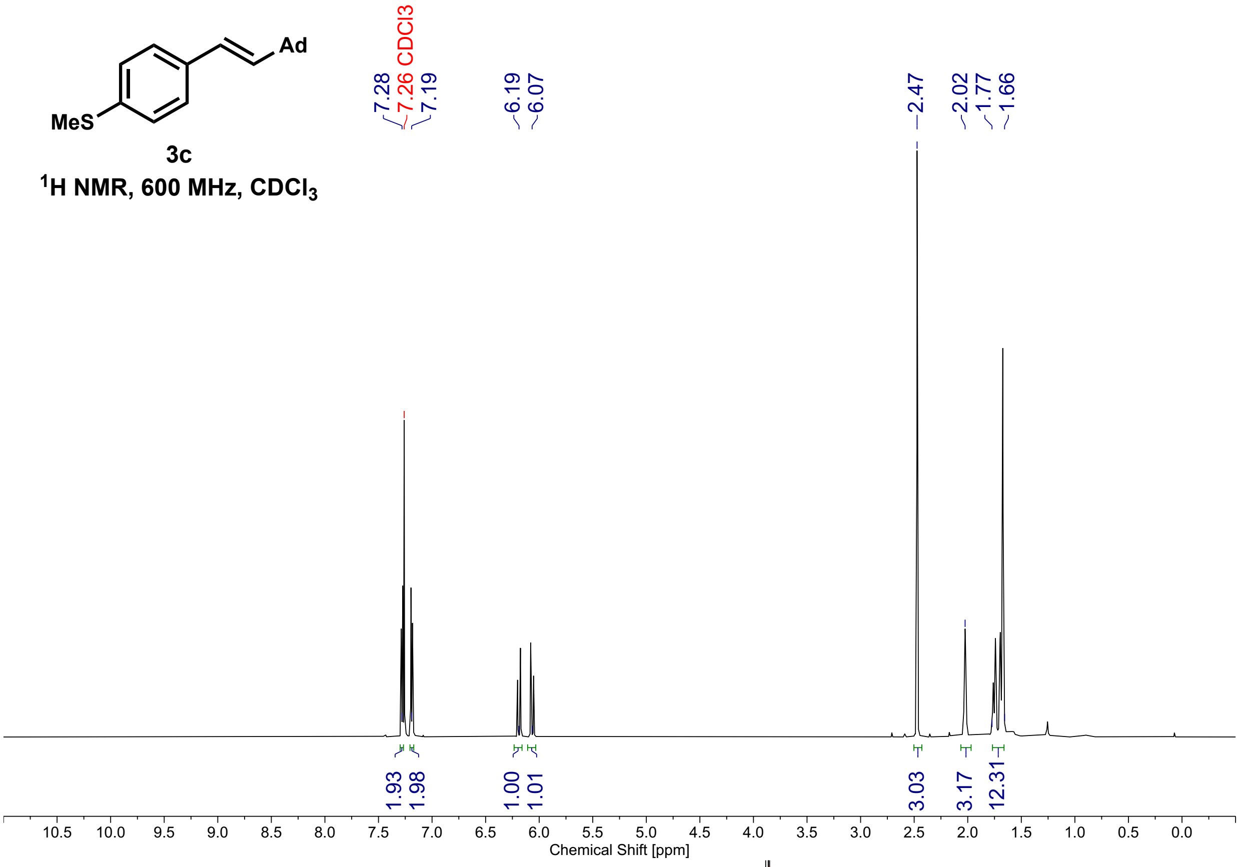

Ad
MeS
3c
^{1}H NMR, 600 MHz, CDCl$_3$
7.28
7.26 CDCl3
7.19
6.19
6.07
2.47
2.02
1.77
1.66
1.93
1.98
1.00
1.01
3.03
3.17
12.31
Chemical Shift [ppm]

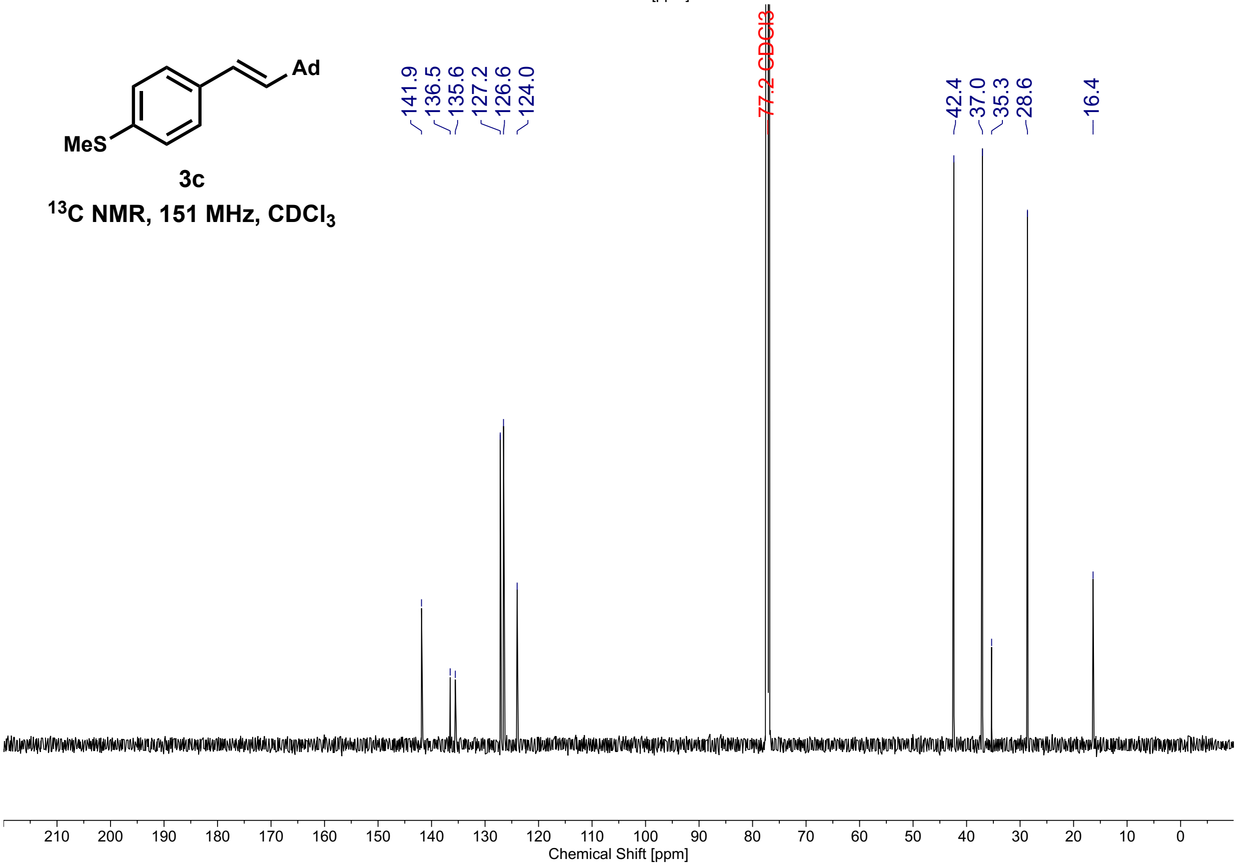

Ad
MeS
3c
^{13}C NMR, 151 MHz, CDCl$_3$
141.9
136.5
135.6
127.2
126.6
124.0
77.2 CDCl3
42.4
37.0
35.3
28.6
16.4
Chemical Shift [ppm]

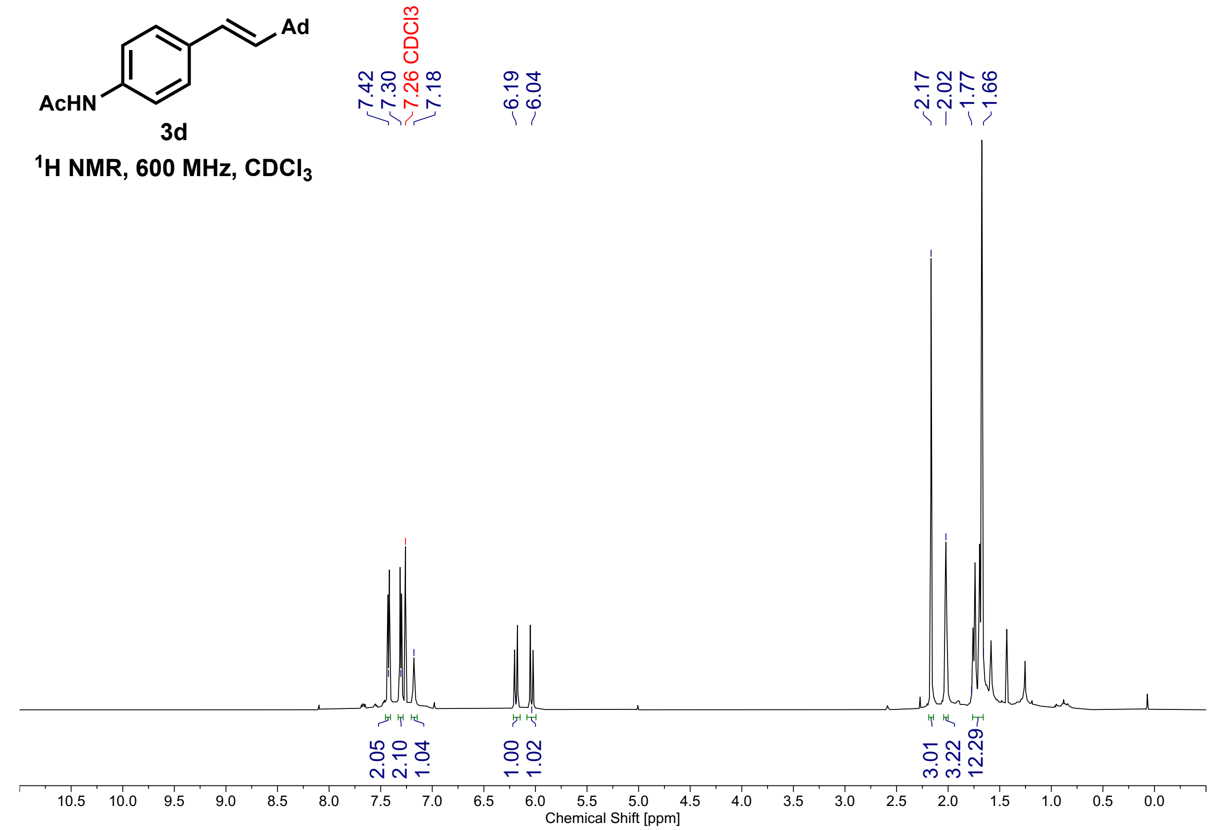
Ad
AcHN
3d
1H NMR, 600 MHz, CDCl3
7.42
7.30
7.26 CDCl3
7.18
6.19
6.04
2.17
2.02
1.77
1.66
2.05
2.10
1.04
1.00
1.02
3.01
3.22
12.29
10.5
10.0
9.5
9.0
8.5
8.0
7.5
7.0
6.5
6.0
5.5
5.0
4.5
4.0
3.5
3.0
2.5
2.0
1.5
1.0
0.5
0.0
Chemical Shift [ppm]

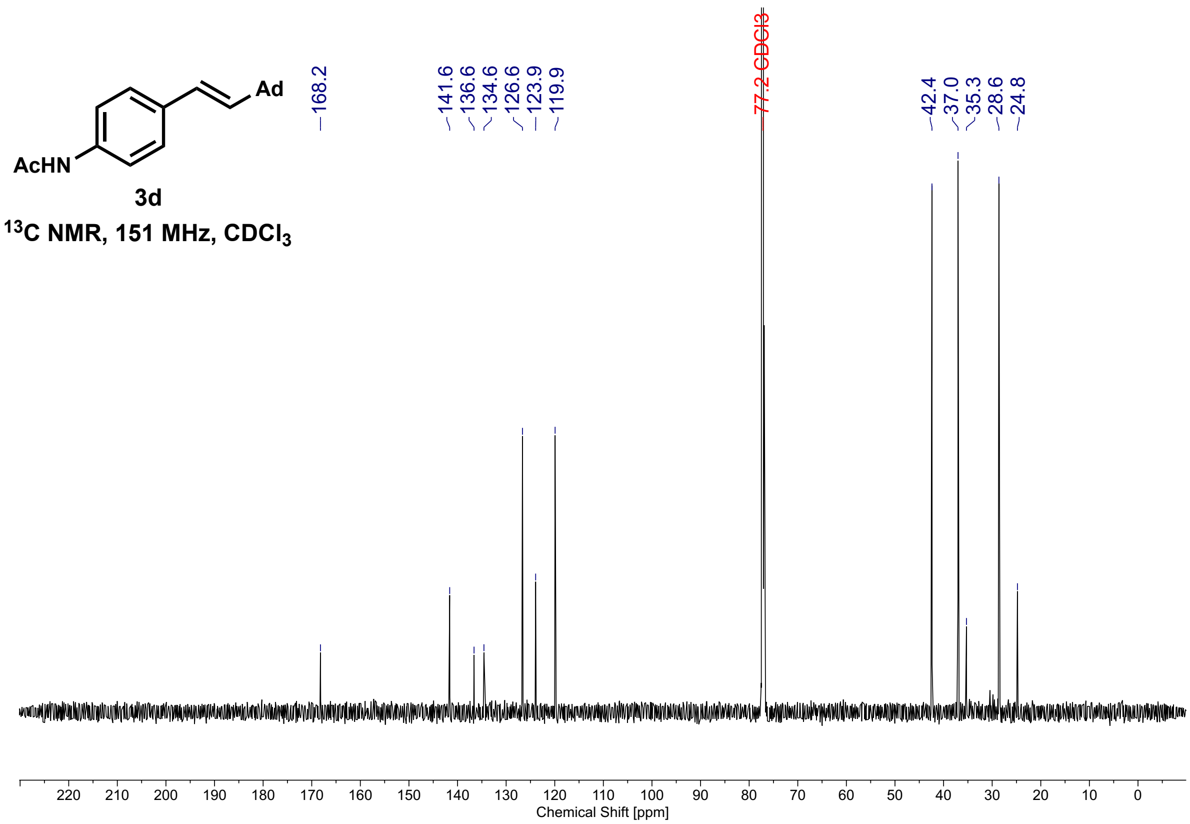
Ad
AcHN
3d
13C NMR, 151 MHz, CDCl3
168.2
141.6
136.6
134.6
126.6
123.9
119.9
77.2 CDCl3
42.4
37.0
35.3
28.6
24.8
220
210
200
190
180
170
160
150
140
130
120
110
100
90
80
70
60
50
40
30
20
10
0
Chemical Shift [ppm]

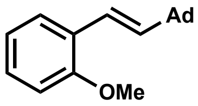

3e

^{1}H NMR, 600 MHz, $CDCl_3$

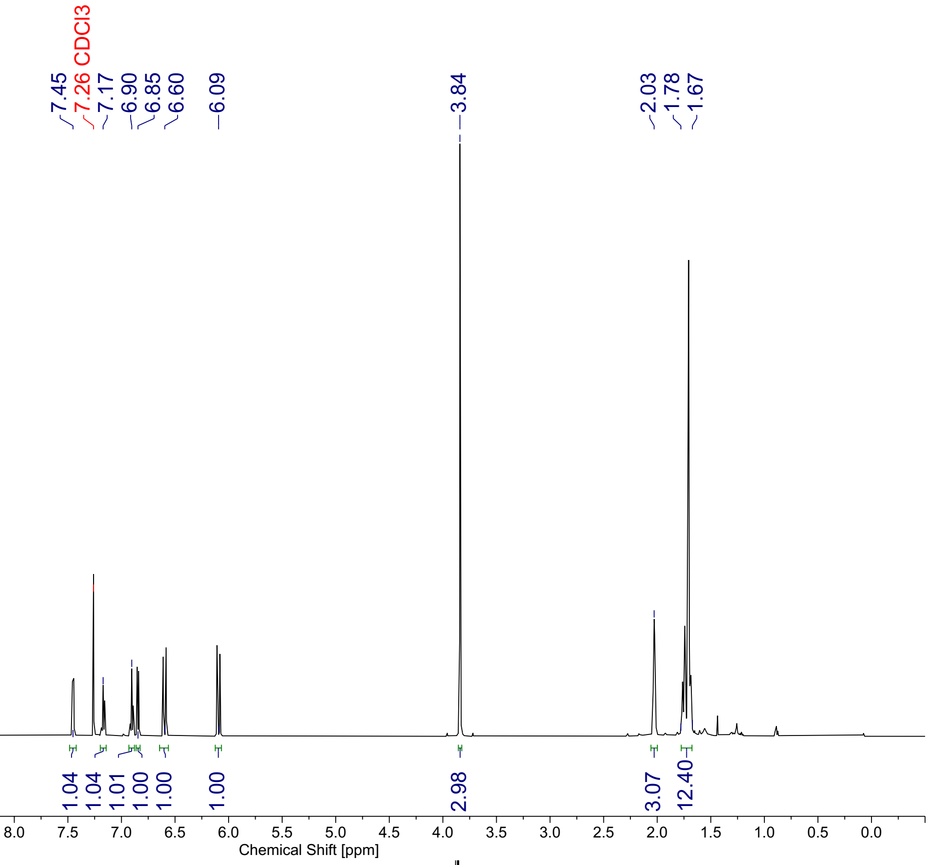

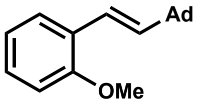

3e

^{13}C NMR, 151 MHz, $CDCl_3$

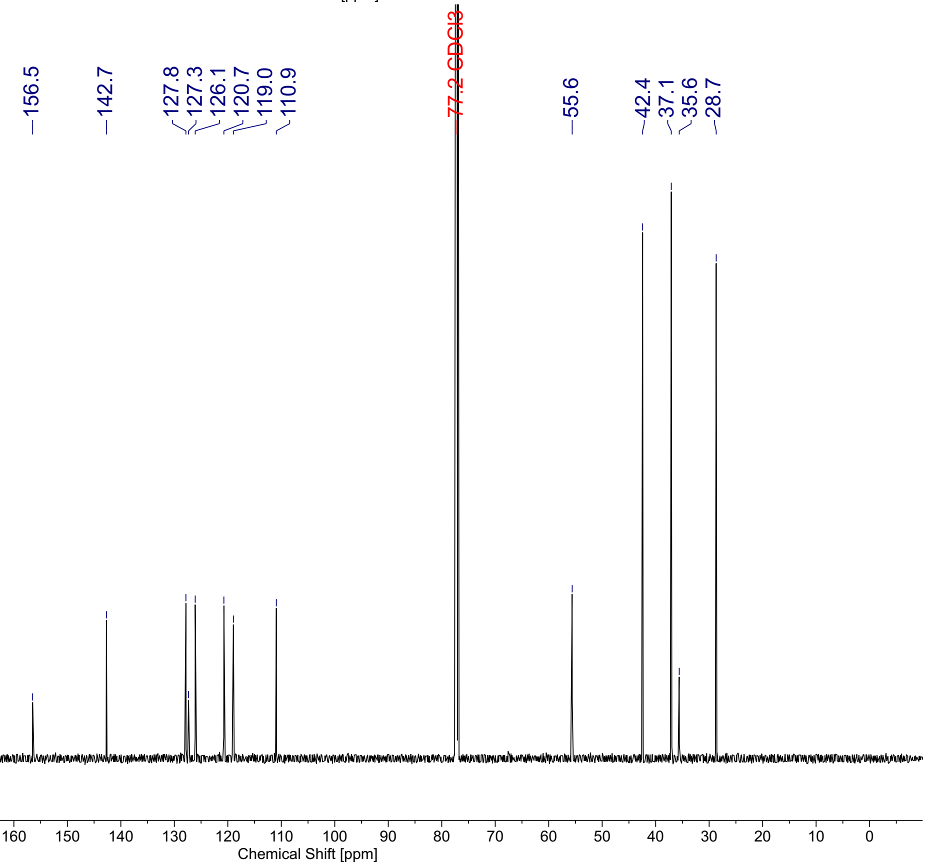

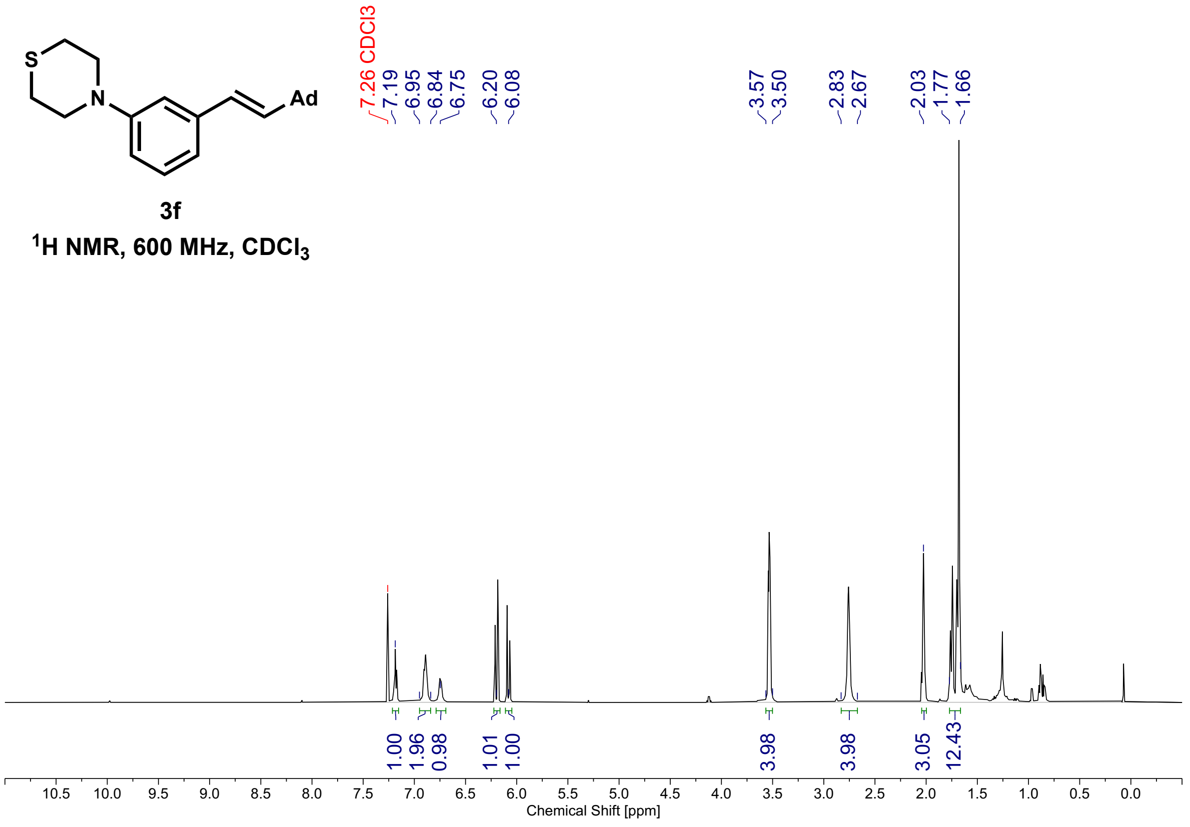
S
N
Ad
3f
¹H NMR, 600 MHz, CDCl₃
7.26 CDCl3
7.19
6.95
6.84
6.75
6.20
6.08
3.57
3.50
2.83
2.67
2.03
1.77
1.66
1.00
1.96
0.98
1.01
1.00
3.98
3.98
3.05
12.43
Chemical Shift [ppm]

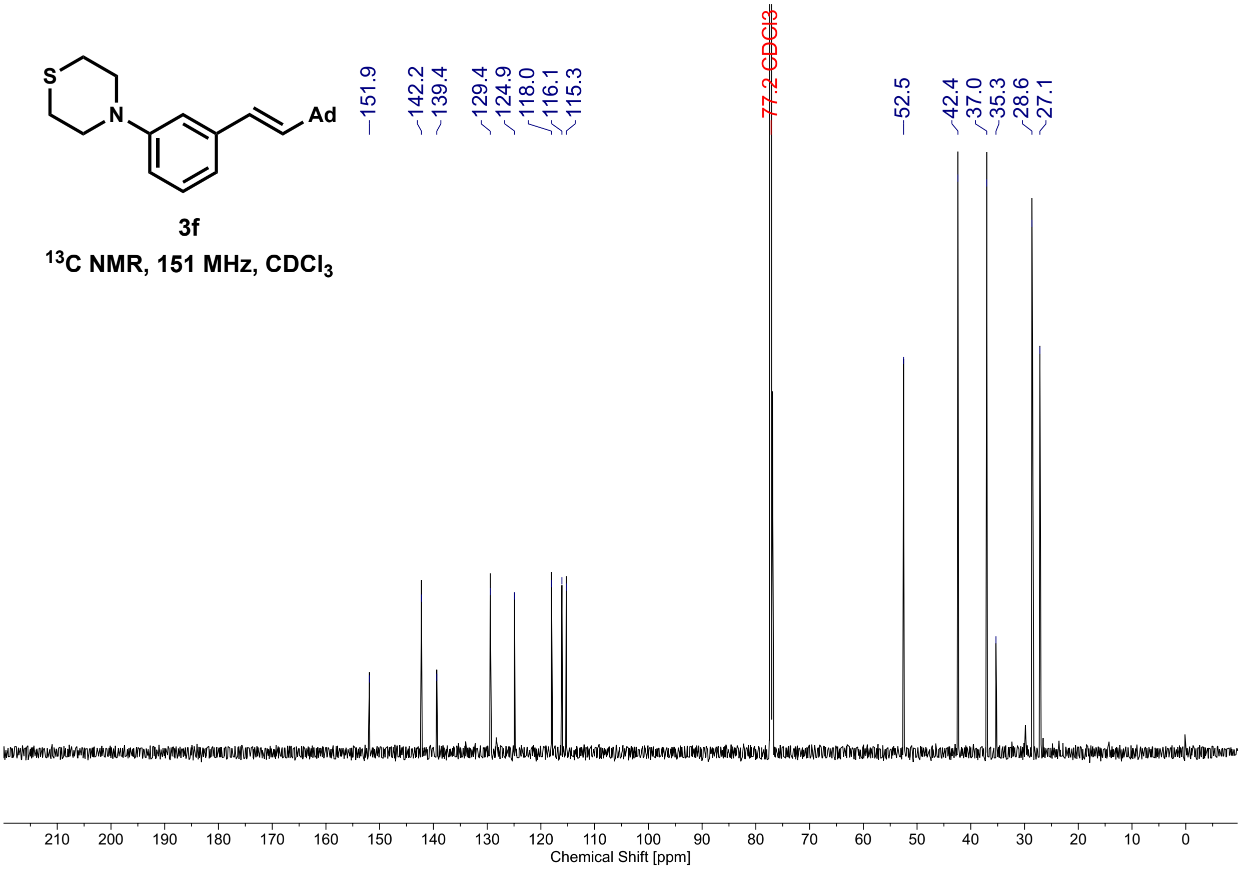
S
N
Ad
3f
¹³C NMR, 151 MHz, CDCl₃
151.9
142.2
139.4
129.4
124.9
118.0
116.1
115.3
77.2 CDCl3
52.5
42.4
37.0
35.3
28.6
27.1
Chemical Shift [ppm]

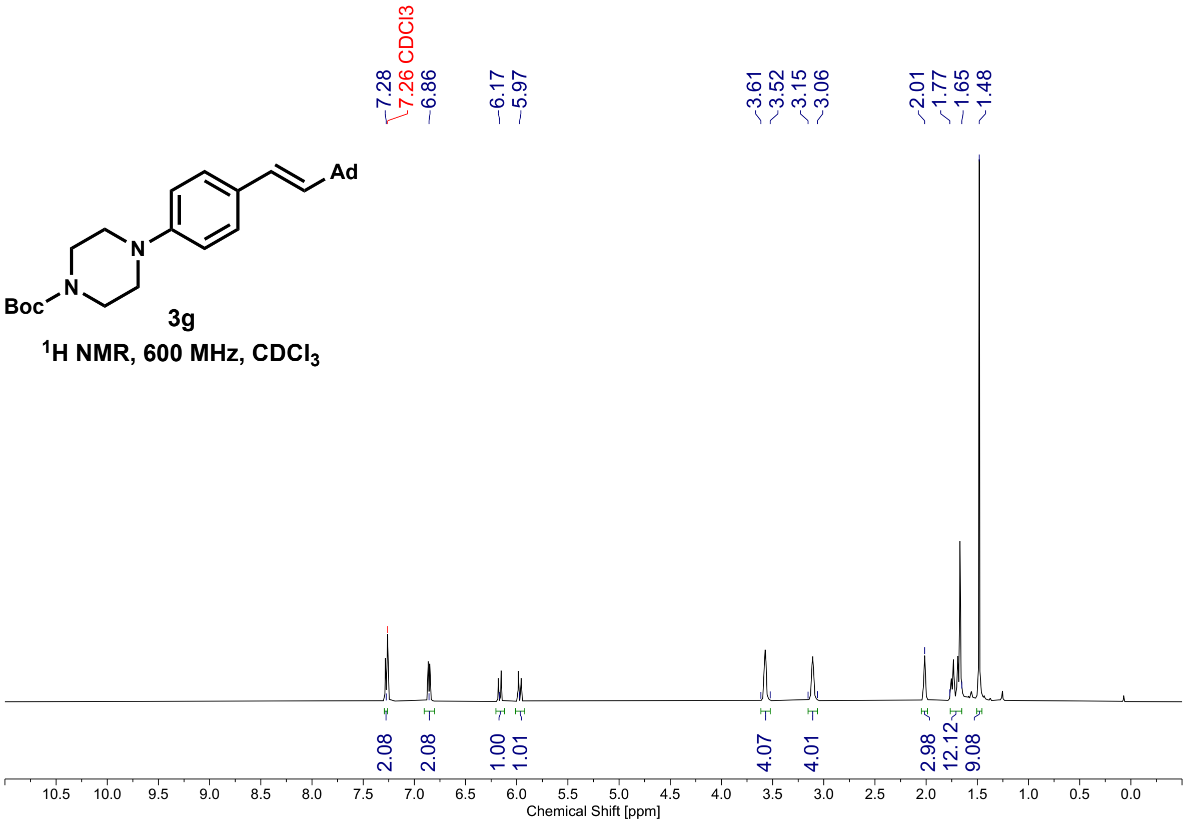
Ad
N
N
Boc
3g
^{1}H NMR, 600 MHz, CDCl$_3$
7.28
7.26 CDCl3
6.86
6.17
5.97
3.61
3.52
3.15
3.06
2.01
1.77
1.65
1.48
2.08
2.08
1.00
1.01
4.07
4.01
2.98
12.12
9.08
10.5 10.0 9.5 9.0 8.5 8.0 7.5 7.0 6.5 6.0 5.5 5.0 4.5 4.0 3.5 3.0 2.5 2.0 1.5 1.0 0.5 0.0
Chemical Shift [ppm]

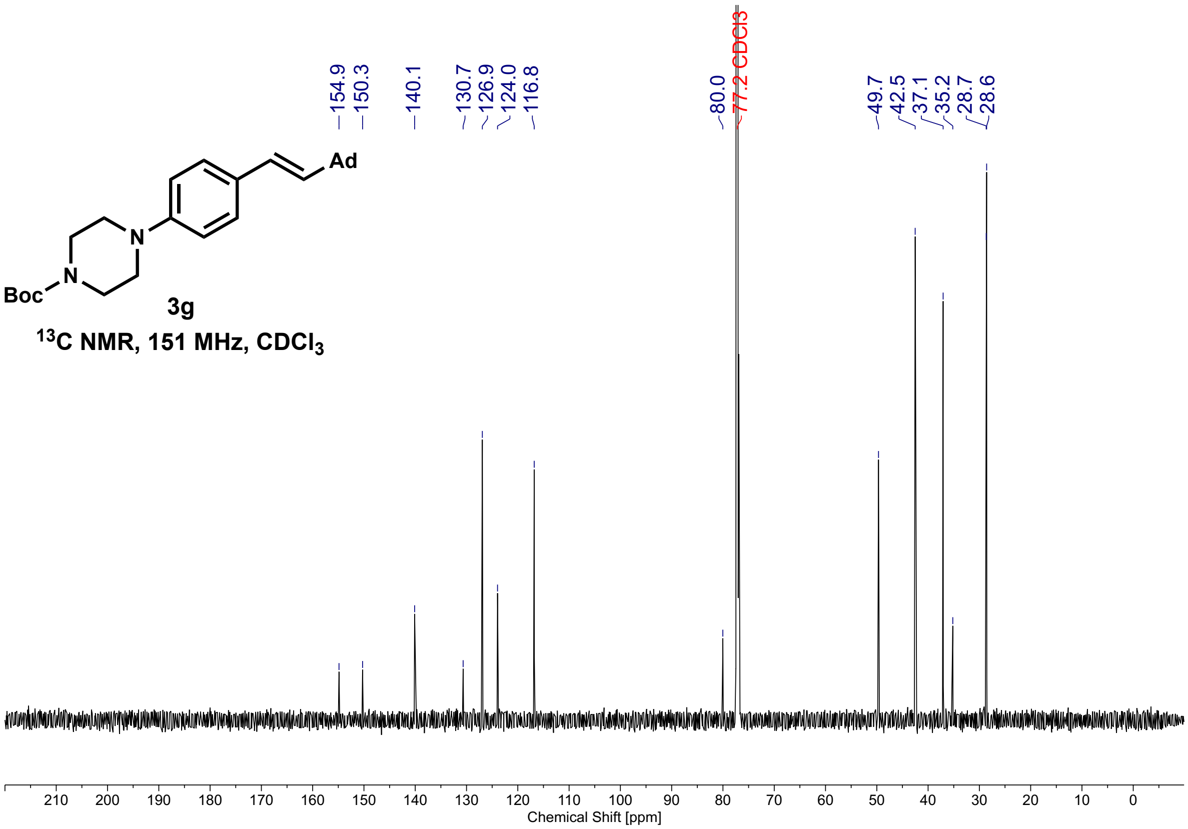
Ad
N
N
Boc
3g
^{13}C NMR, 151 MHz, CDCl$_3$
154.9
150.3
140.1
130.7
126.9
124.0
116.8
80.0
77.2 CDCl3
49.7
42.5
37.1
35.2
28.7
28.6
210 200 190 180 170 160 150 140 130 120 110 100 90 80 70 60 50 40 30 20 10 0
Chemical Shift [ppm]

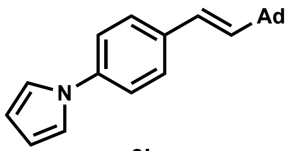

3h

^{1}H NMR, 600 MHz, $CDCl_3$

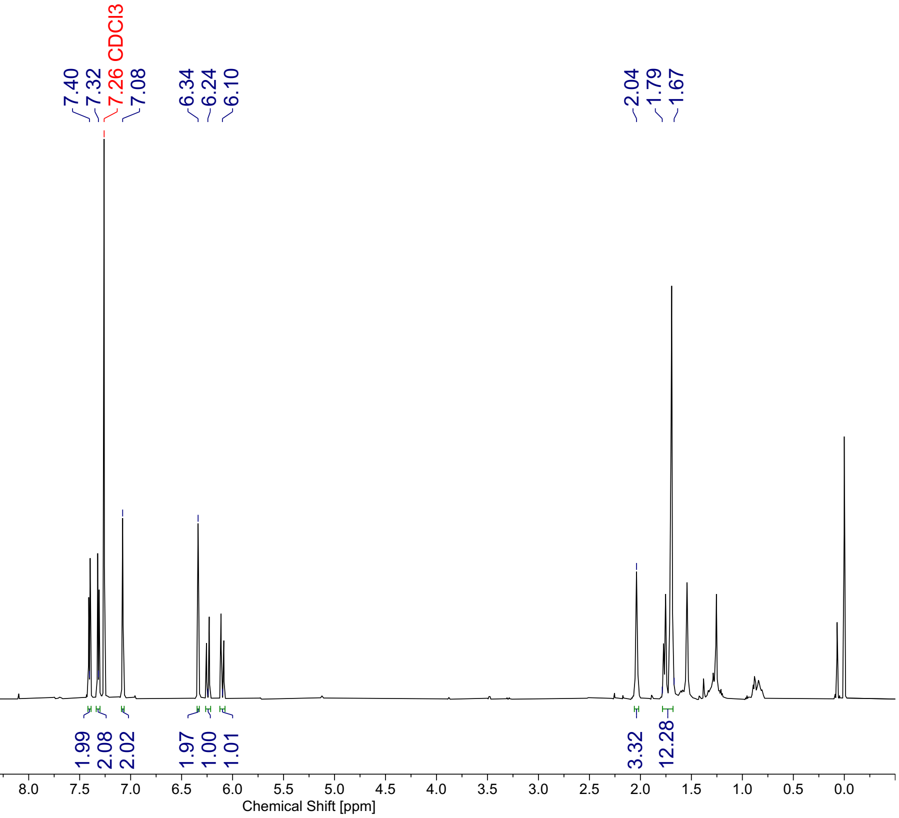

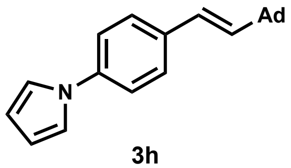

3h

^{13}C NMR, 151 MHz, $CDCl_3$

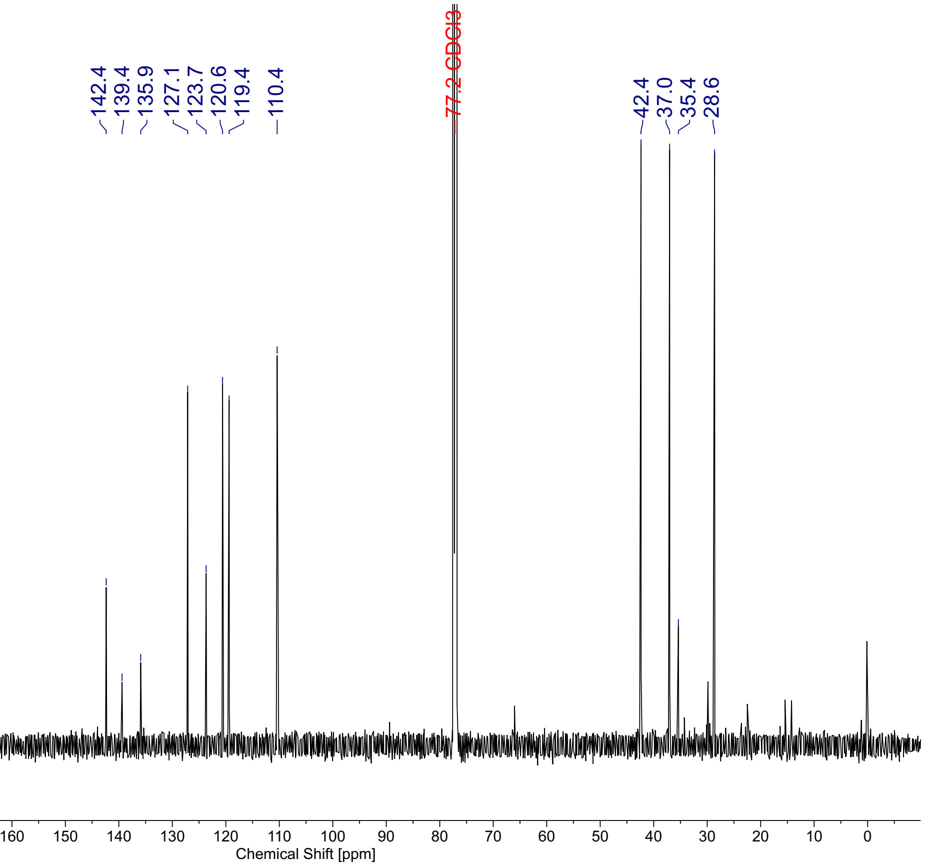

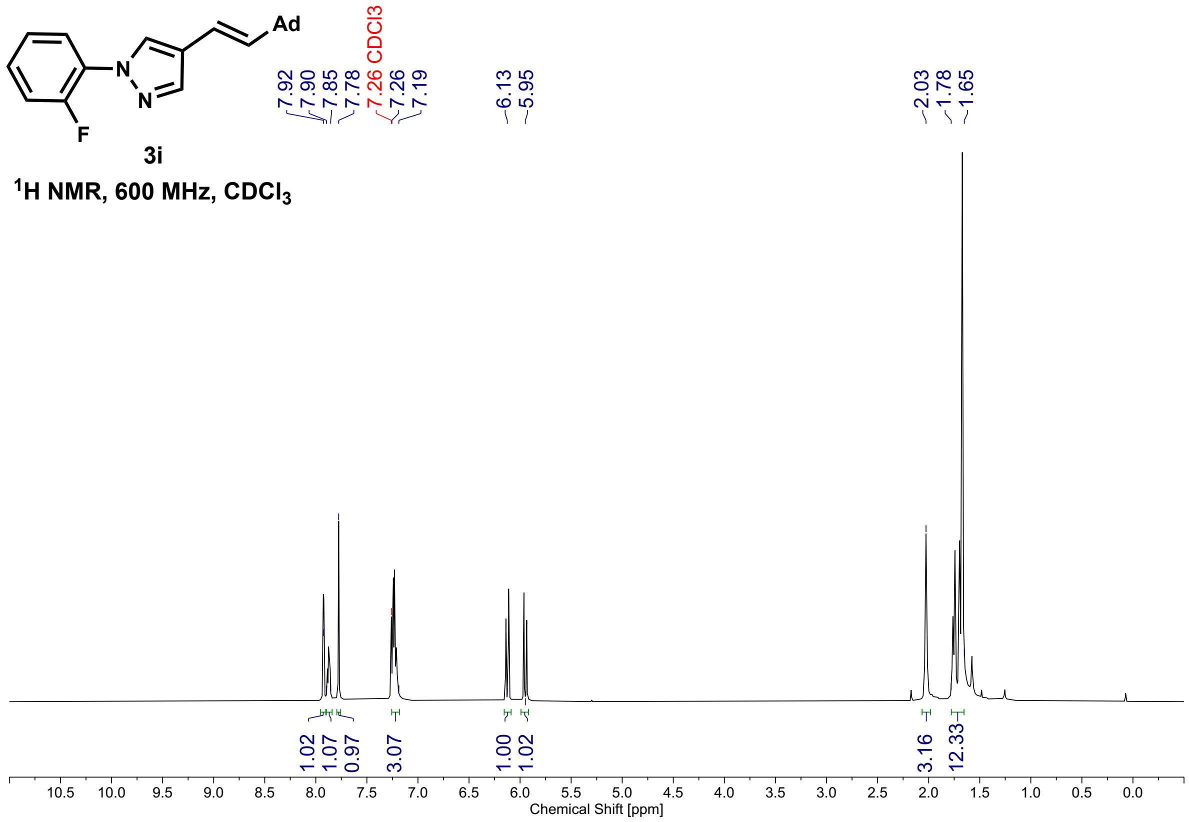
Ad
N
N
F
3i
1H NMR, 600 MHz, CDCl3
7.92
7.90
7.85
7.78
7.26 CDCl3
7.26
7.19
6.13
5.95
2.03
1.78
1.65
1.02
1.07
0.97
3.07
1.00
1.02
3.16
12.33
10.5 10.0 9.5 9.0 8.5 8.0 7.5 7.0 6.5 6.0 5.5 5.0 4.5 4.0 3.5 3.0 2.5 2.0 1.5 1.0 0.5 0.0
Chemical Shift [ppm]

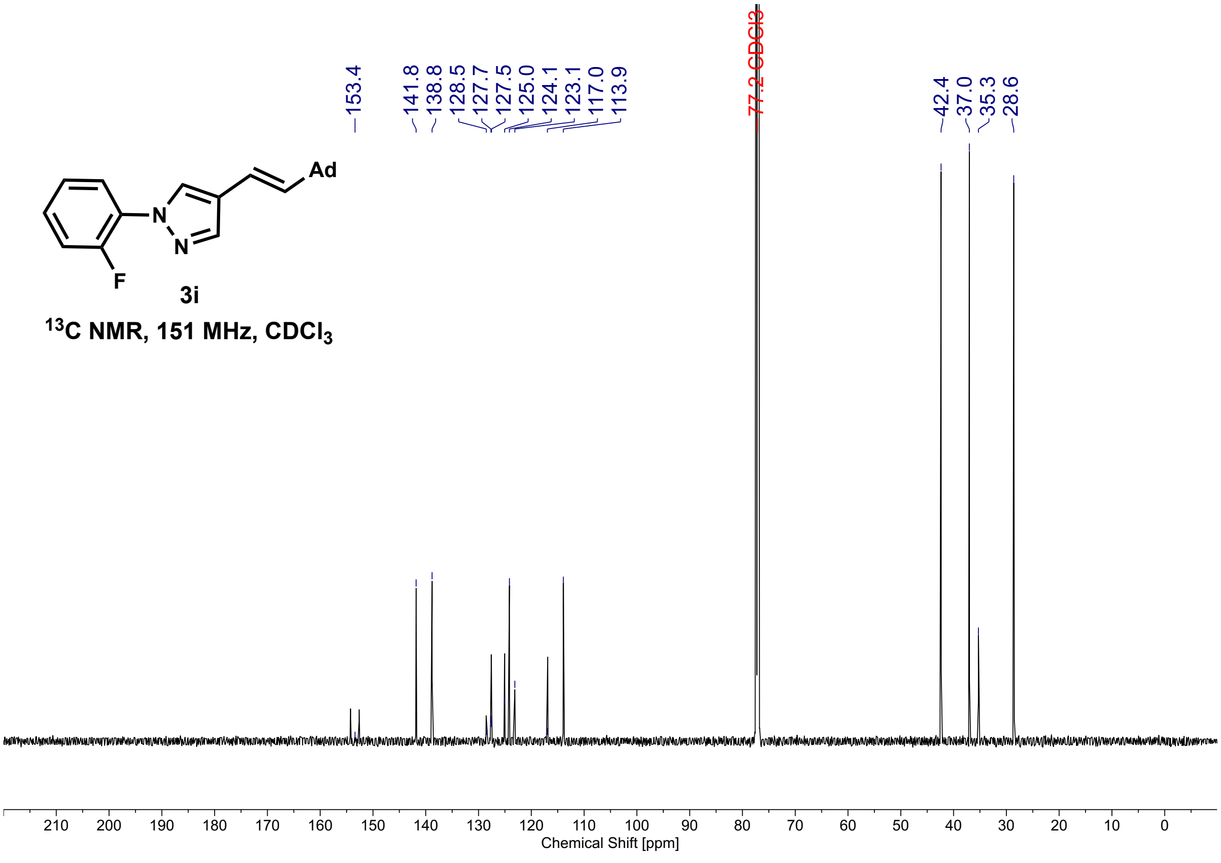
153.4
141.8
138.8
128.5
127.7
127.5
125.0
124.1
123.1
117.0
113.9
77.2 CDCl3
42.4
37.0
35.3
28.6
Ad
N
N
F
3i
13C NMR, 151 MHz, CDCl3
210 200 190 180 170 160 150 140 130 120 110 100 90 80 70 60 50 40 30 20 10 0
Chemical Shift [ppm]

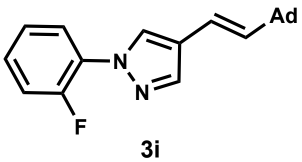

3i

^{19}F NMR, 471 MHz, $CDCl_3$

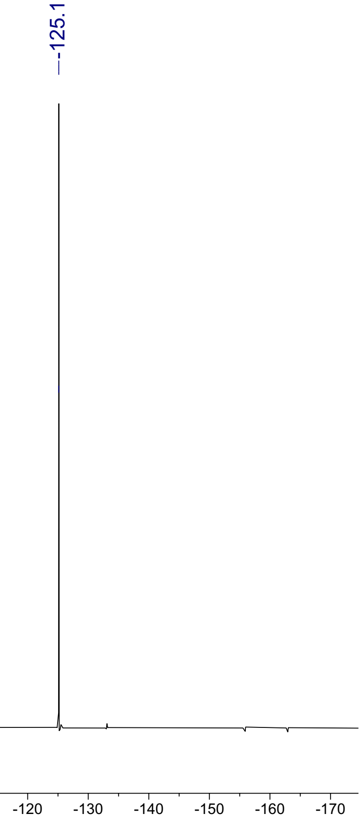

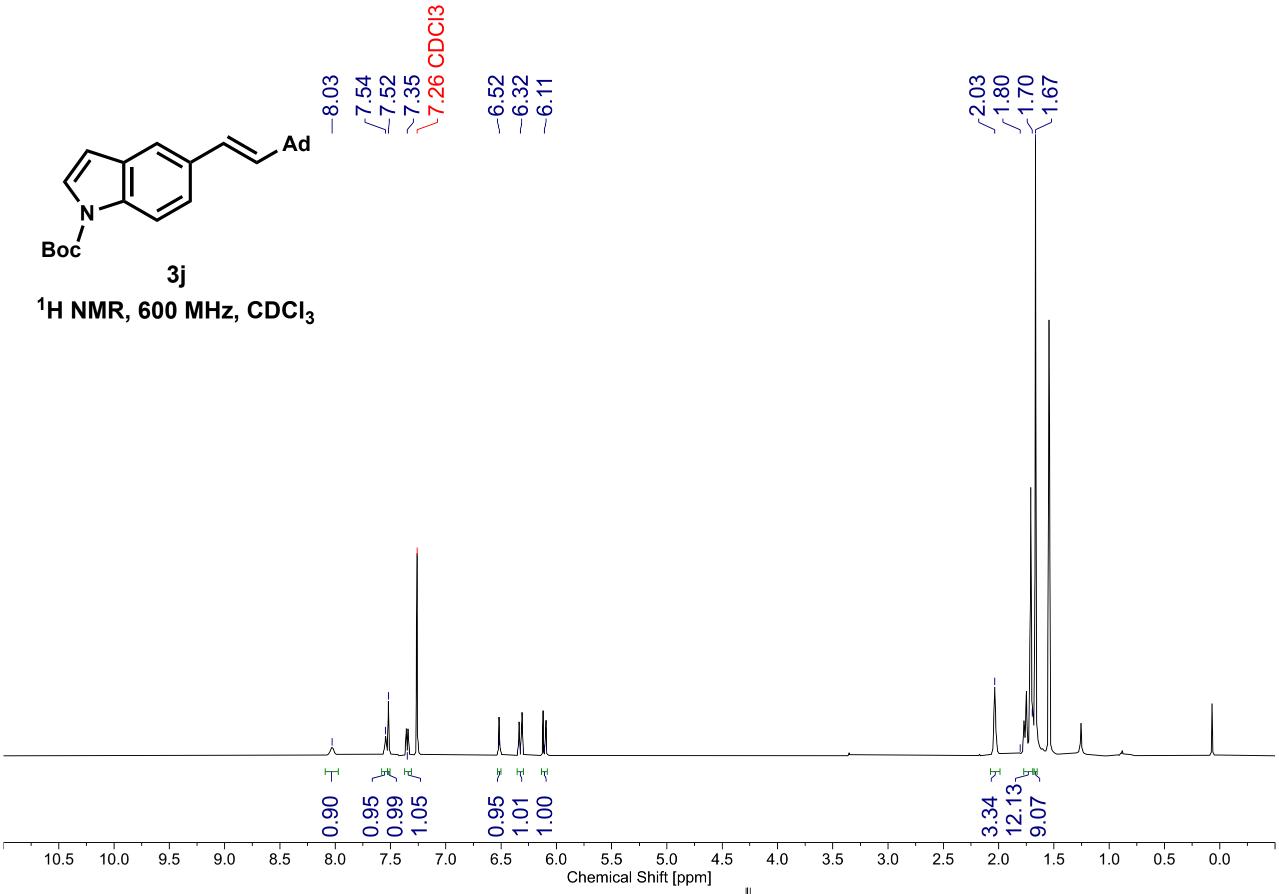

3j

^{1}H NMR, 600 MHz, $CDCl_3$

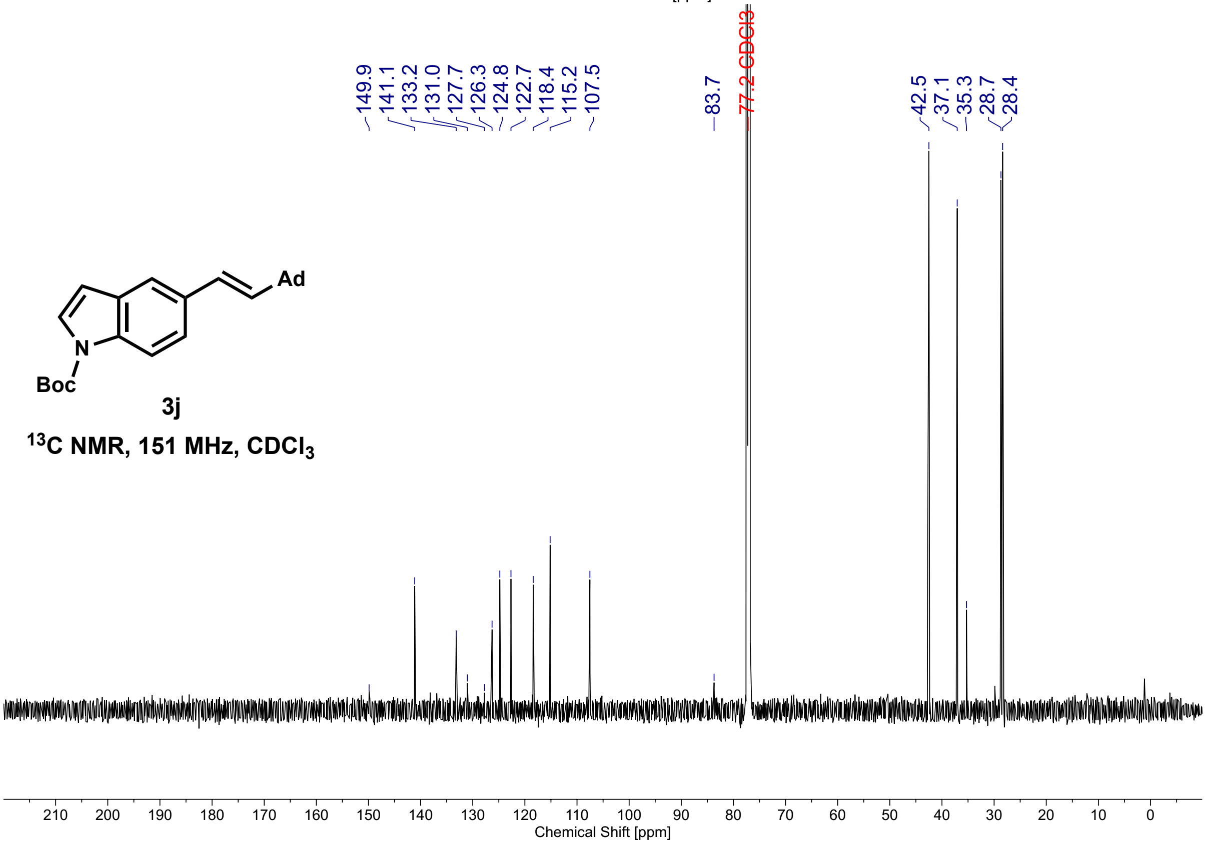

3j

^{13}C NMR, 151 MHz, $CDCl_3$

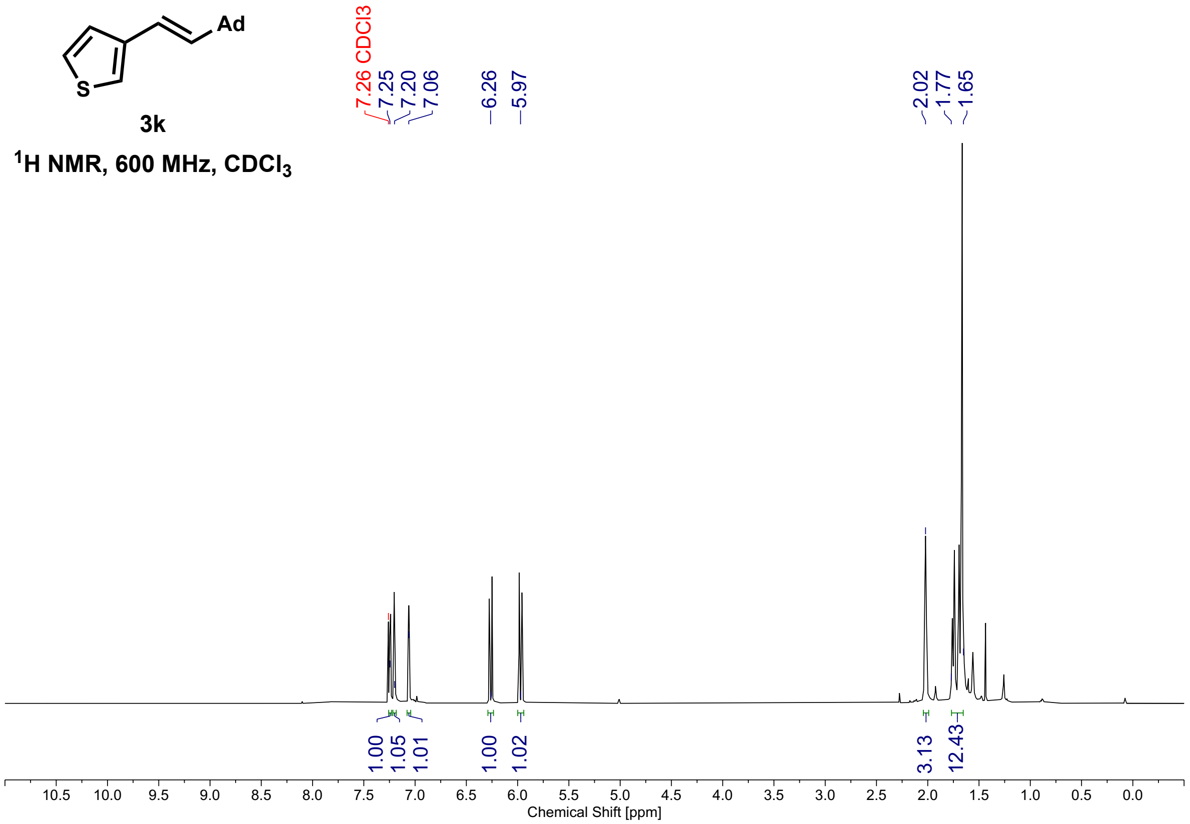
Ad
S
3k
1H NMR, 600 MHz, CDCl3
7.26 CDCl3
7.25
7.20
7.06
6.26
5.97
2.02
1.77
1.65
1.00
1.05
1.01
1.00
1.02
3.13
12.43
10.5 10.0 9.5 9.0 8.5 8.0 7.5 7.0 6.5 6.0 5.5 5.0 4.5 4.0 3.5 3.0 2.5 2.0 1.5 1.0 0.5 0.0
Chemical Shift [ppm]

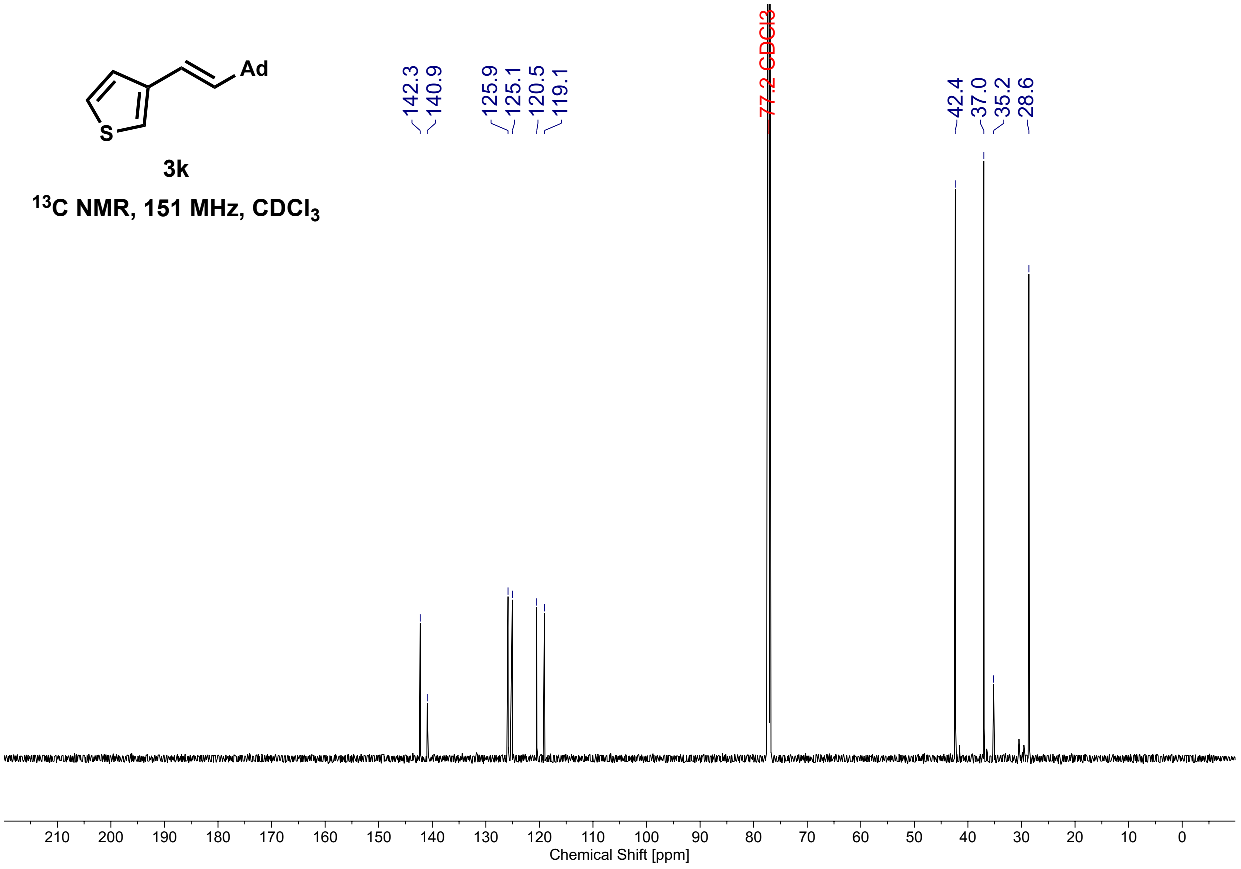
Ad
S
3k
13C NMR, 151 MHz, CDCl3
142.3
140.9
125.9
125.1
120.5
119.1
77.2 CDCl3
42.4
37.0
35.2
28.6
210 200 190 180 170 160 150 140 130 120 110 100 90 80 70 60 50 40 30 20 10 0
Chemical Shift [ppm]

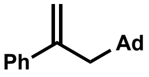

3l

^{1}H NMR, 600 MHz, $CDCl_3$

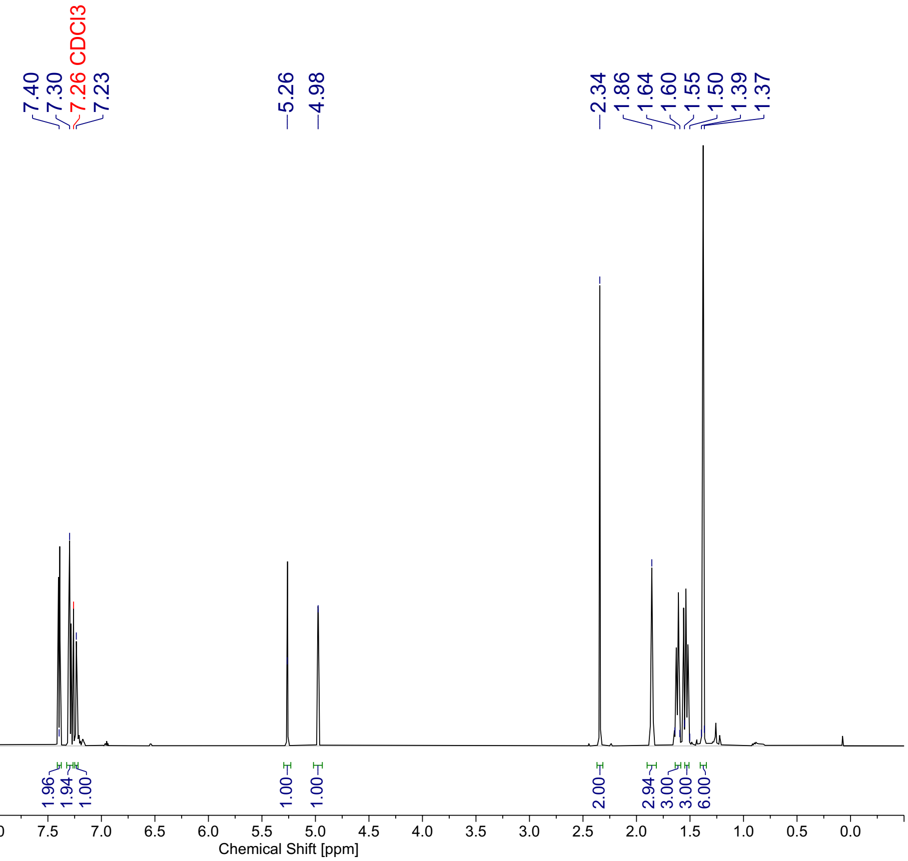

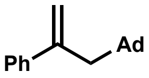

3l

^{13}C NMR, 151 MHz, $CDCl_3$

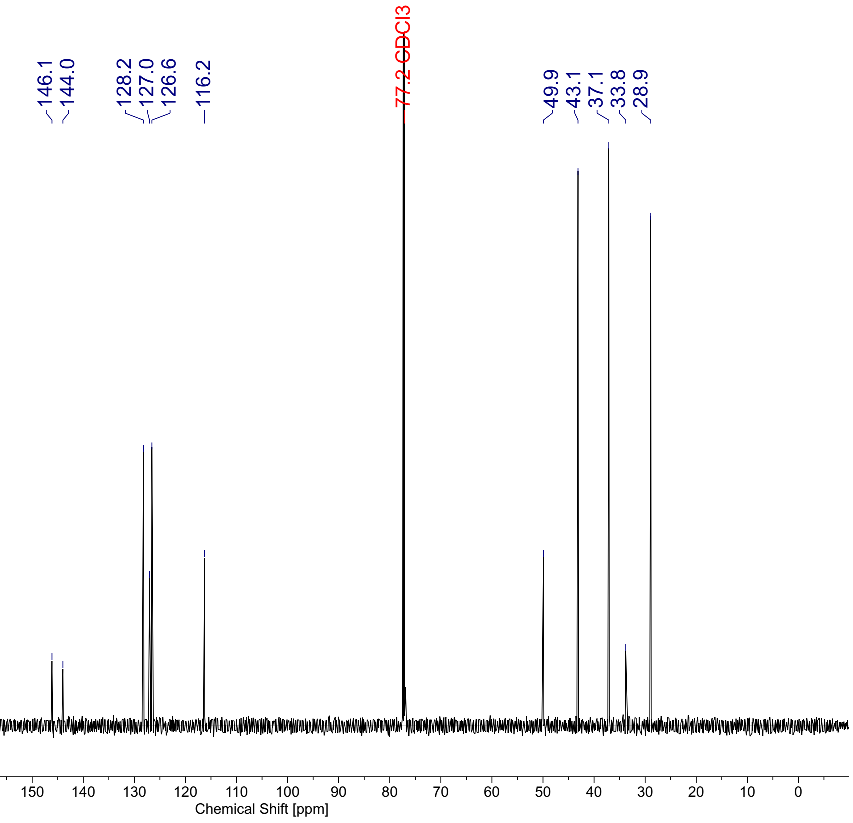

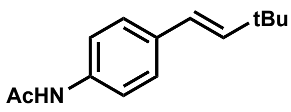

3m

^{1}H NMR, 600 MHz, $CDCl_3$

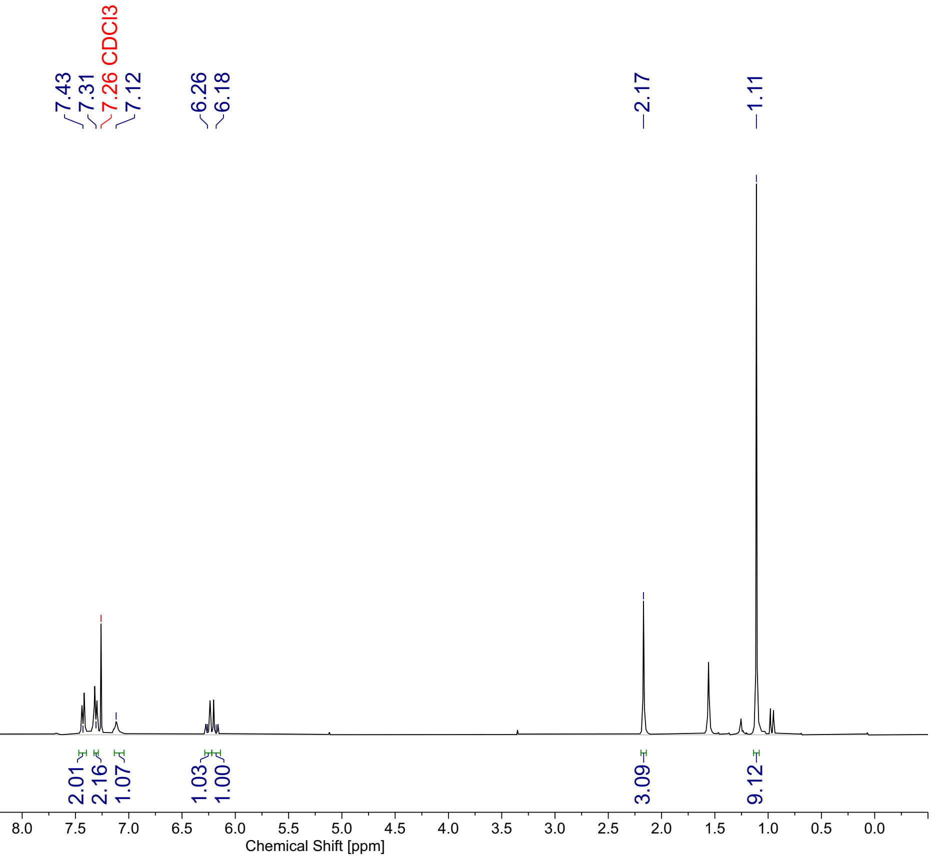

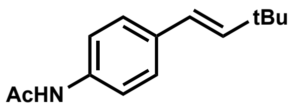

3m

^{13}C NMR, 151 MHz, $CDCl_3$

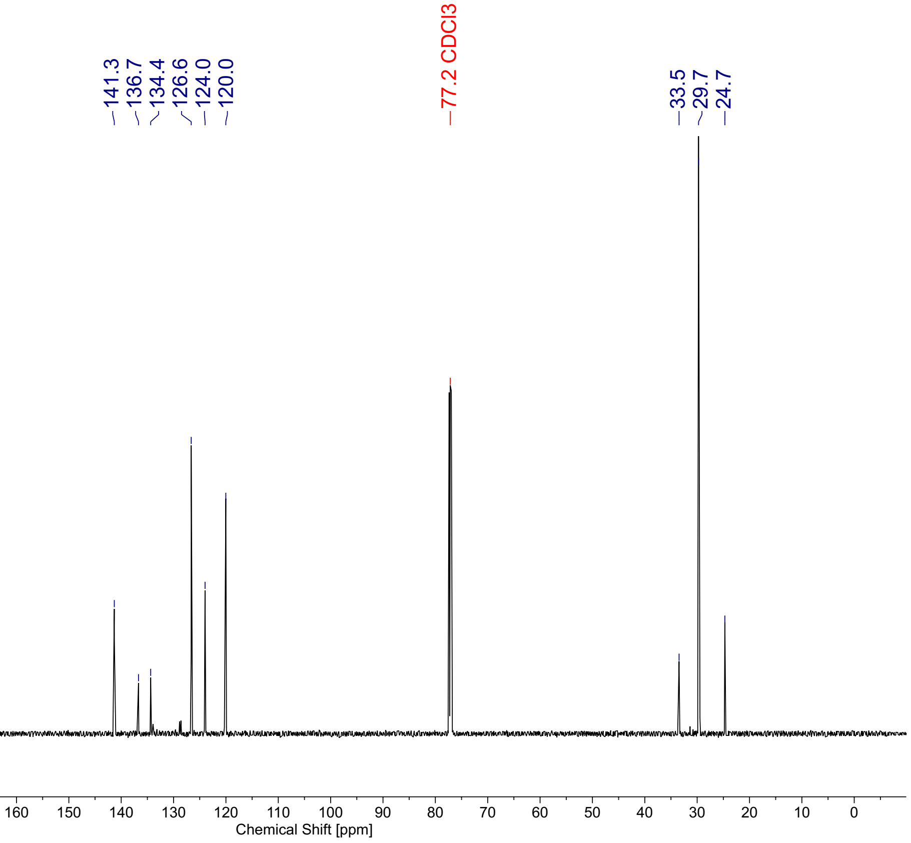

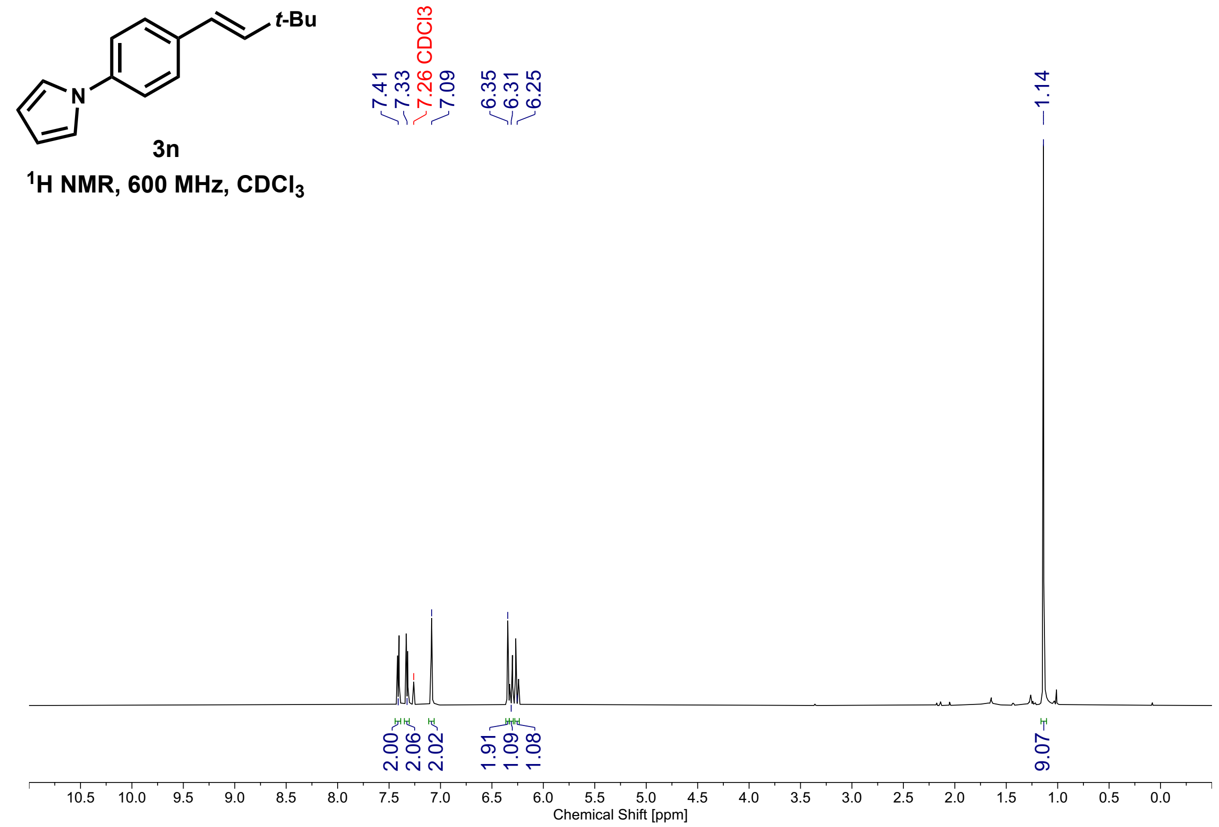
t-Bu
N
3n
1H NMR, 600 MHz, CDCl3
7.41
7.33
7.26 CDCl3
7.09
6.35
6.31
6.25
1.14
2.00
2.06
2.02
1.91
1.09
1.08
9.07
Chemical Shift [ppm]

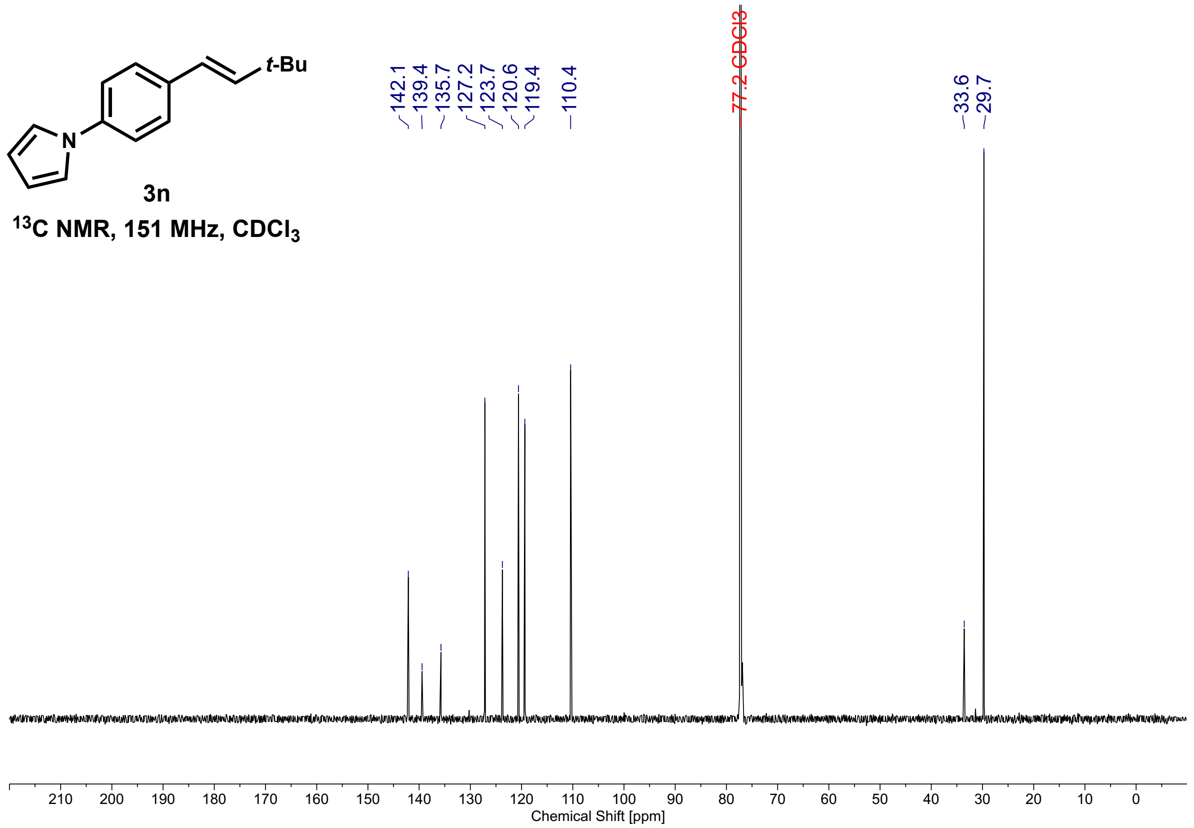
t-Bu
N
3n
13C NMR, 151 MHz, CDCl3
142.1
139.4
135.7
127.2
123.7
120.6
119.4
110.4
77.2 CDCl3
33.6
29.7
Chemical Shift [ppm]

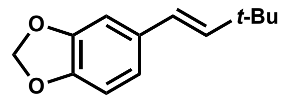

3o

^{1}H NMR, 600 MHz, CDCl$_3$

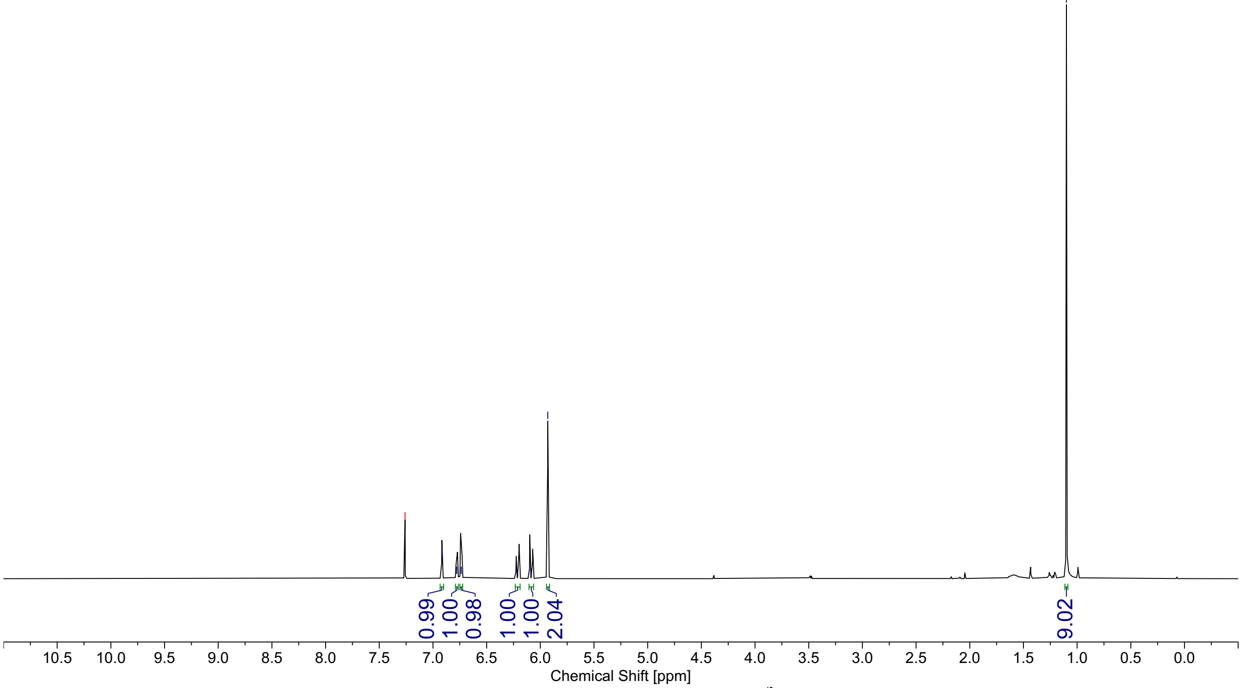

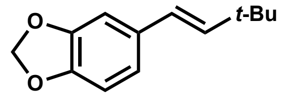

3o

^{13}C NMR, 151 MHz, CDCl$_3$

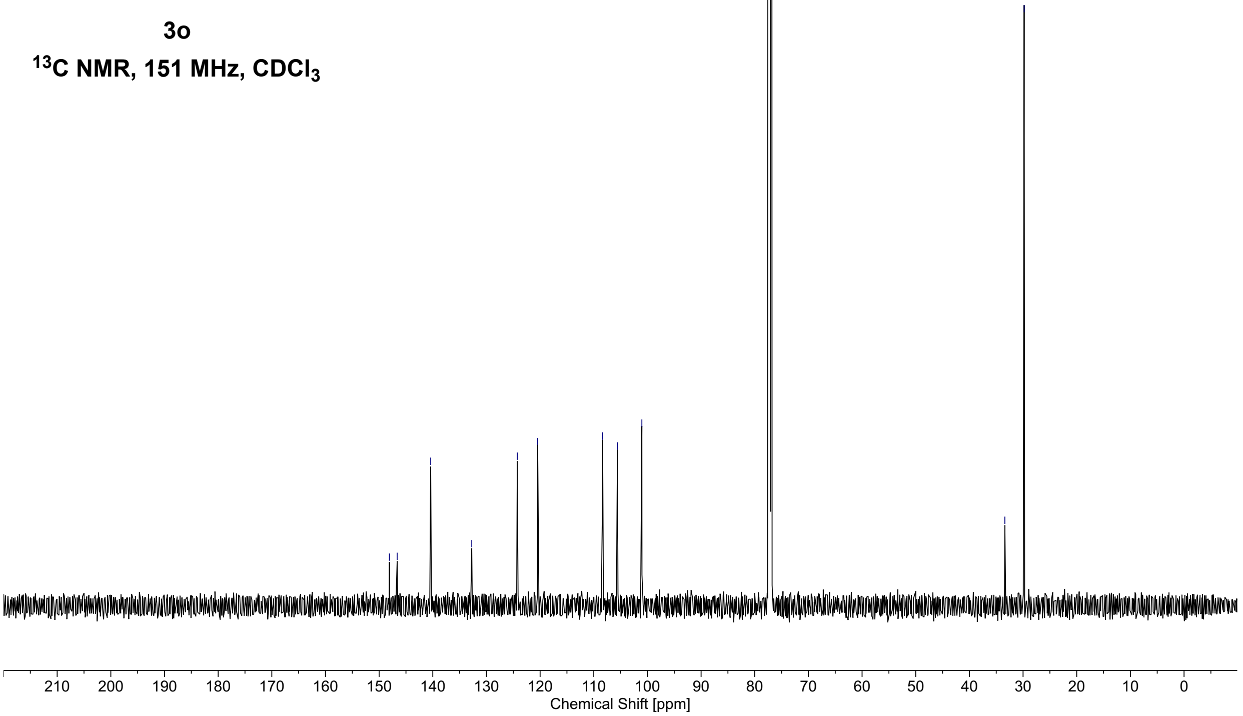

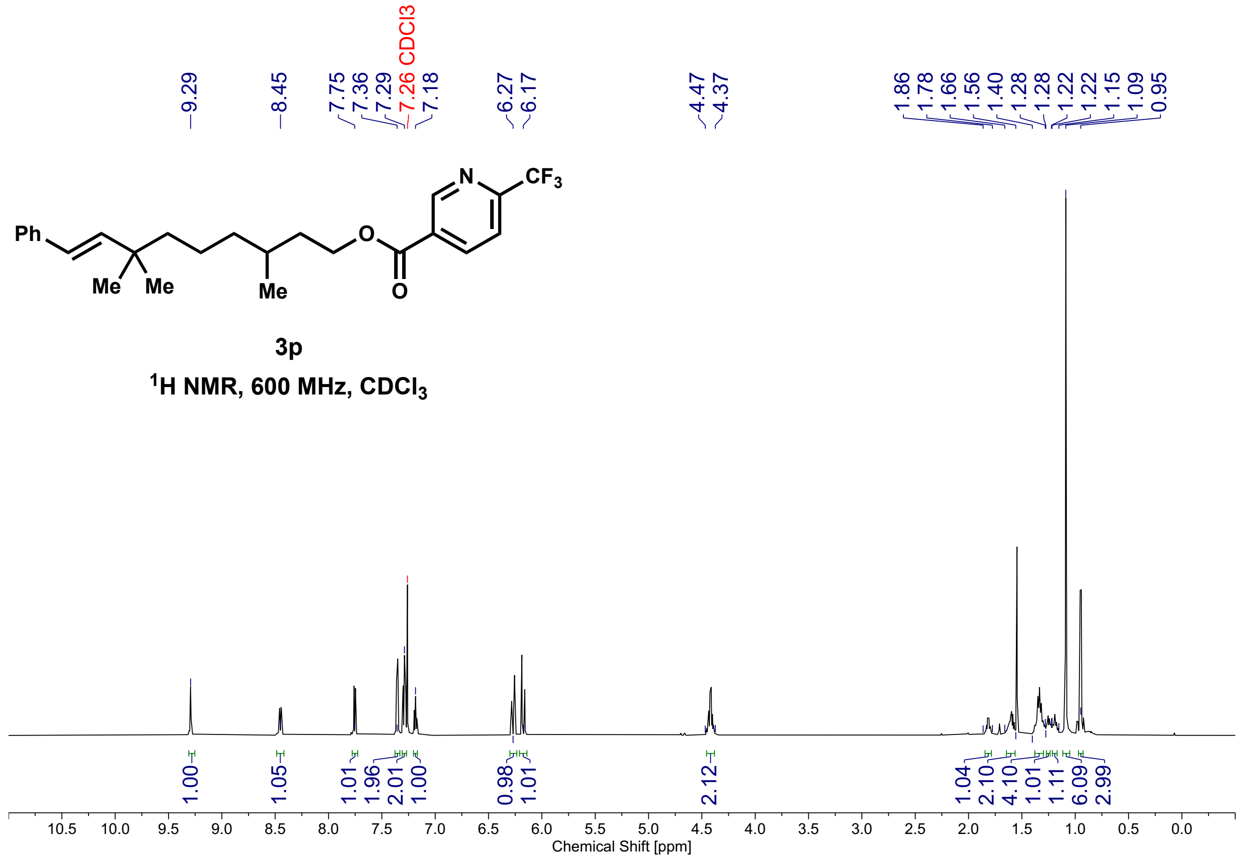
3p
^{1}H NMR, 600 MHz, $CDCl_3$
9.29
8.45
7.75
7.36
7.29
7.26 CDCl3
7.18
6.27
6.17
4.47
4.37
1.86
1.78
1.66
1.56
1.40
1.28
1.28
1.22
1.22
1.15
1.09
0.95
1.00
1.05
1.01
1.96
2.01
1.00
0.98
1.01
2.12
1.04
2.10
4.10
1.01
1.11
6.09
2.99
Chemical Shift [ppm]

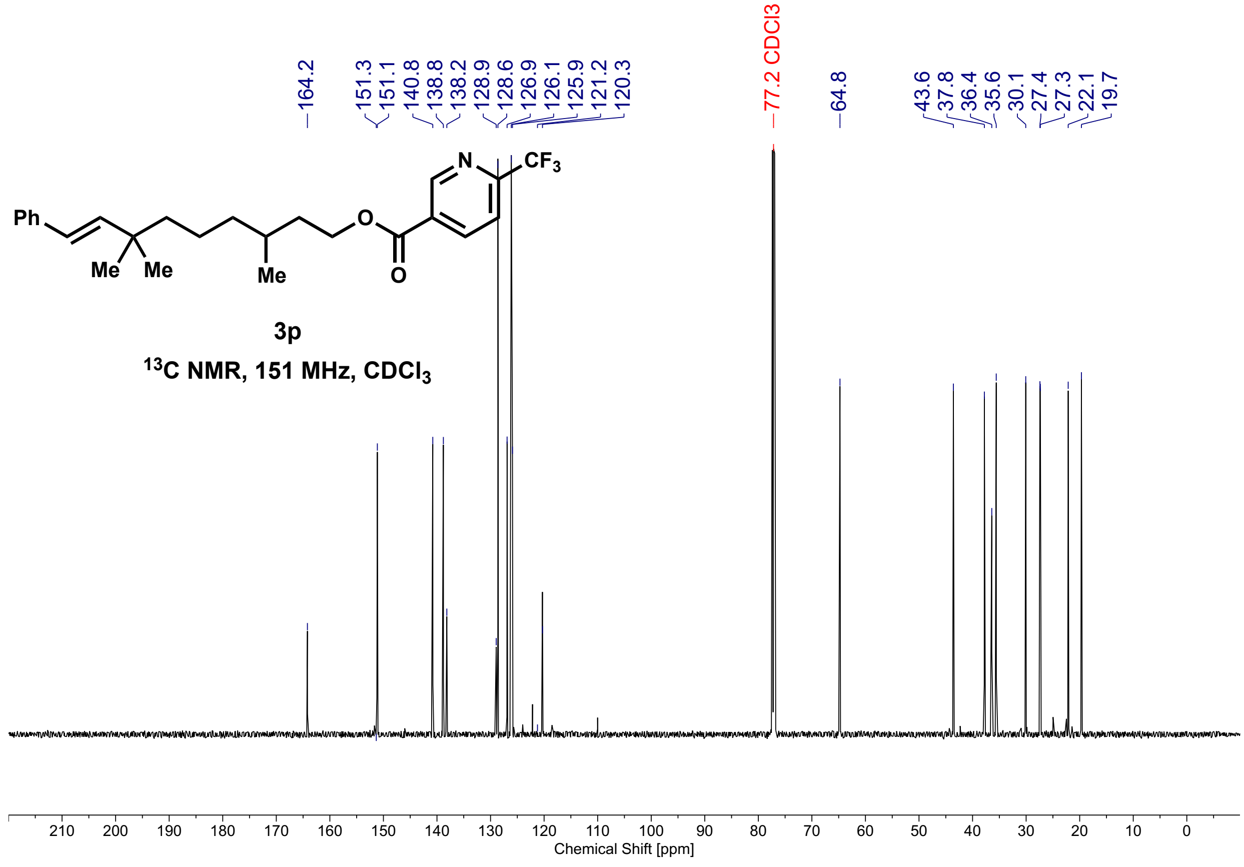
3p
^{13}C NMR, 151 MHz, $CDCl_3$
164.2
151.3
151.1
140.8
138.8
138.2
128.9
128.6
126.9
126.1
125.9
121.2
120.3
77.2 CDCl3
64.8
43.6
37.8
36.4
35.6
30.1
27.4
27.3
22.1
19.7
Chemical Shift [ppm]

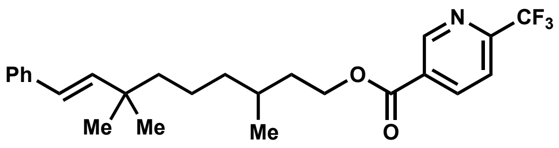

3p

^{19}F NMR, 471 MHz, $CDCl_3$

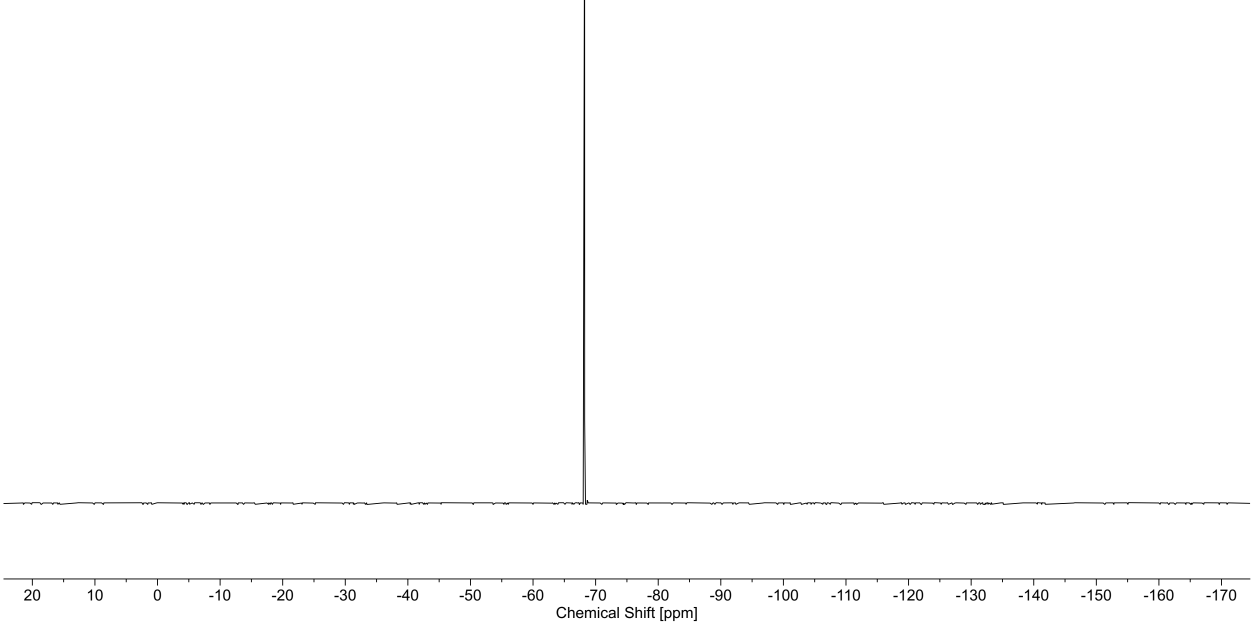

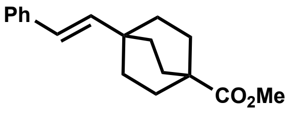

3q

^{1}H NMR, 600 MHz, $CDCl_3$

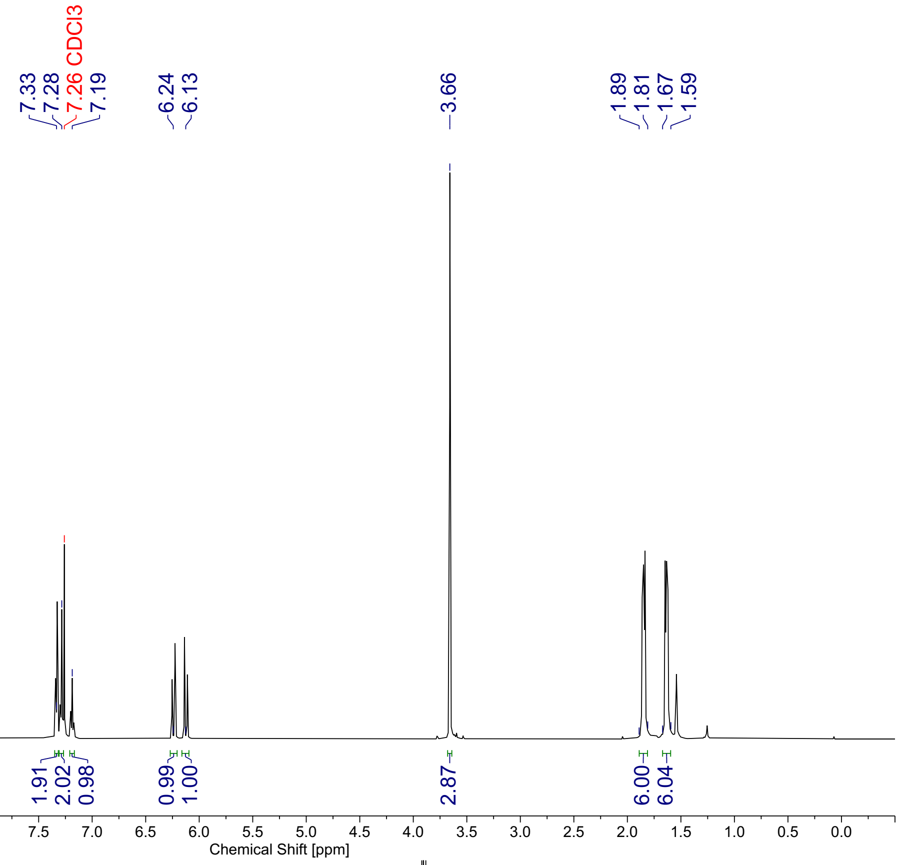

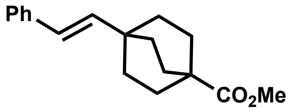

3q

^{13}C NMR, 151 MHz, $CDCl_3$

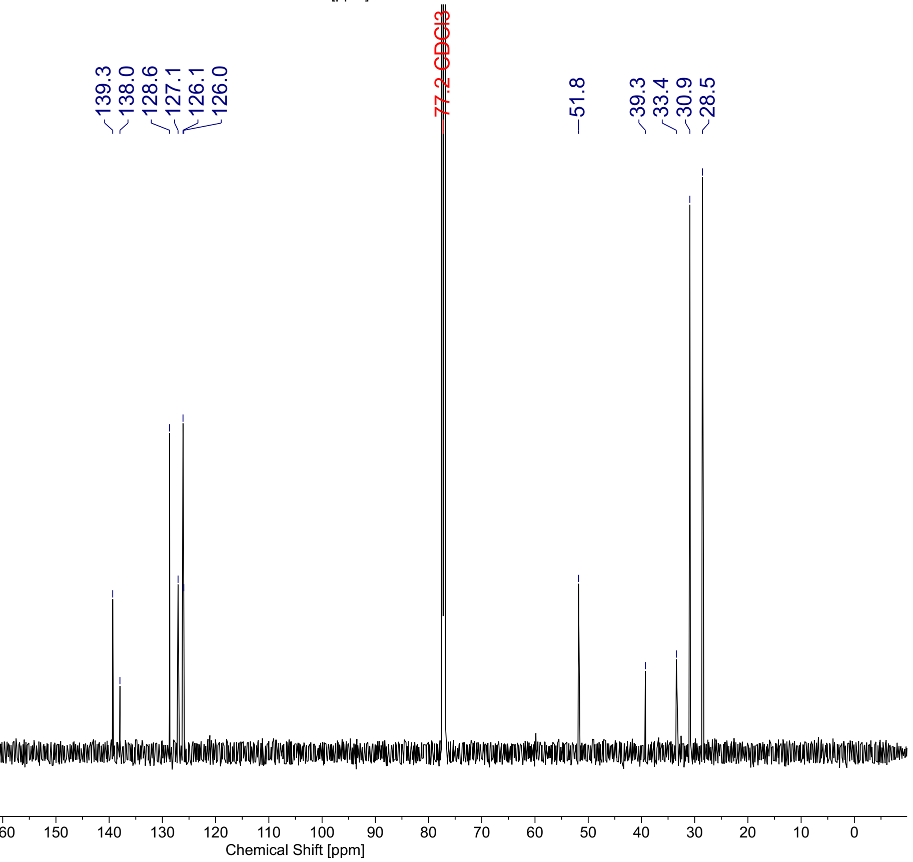

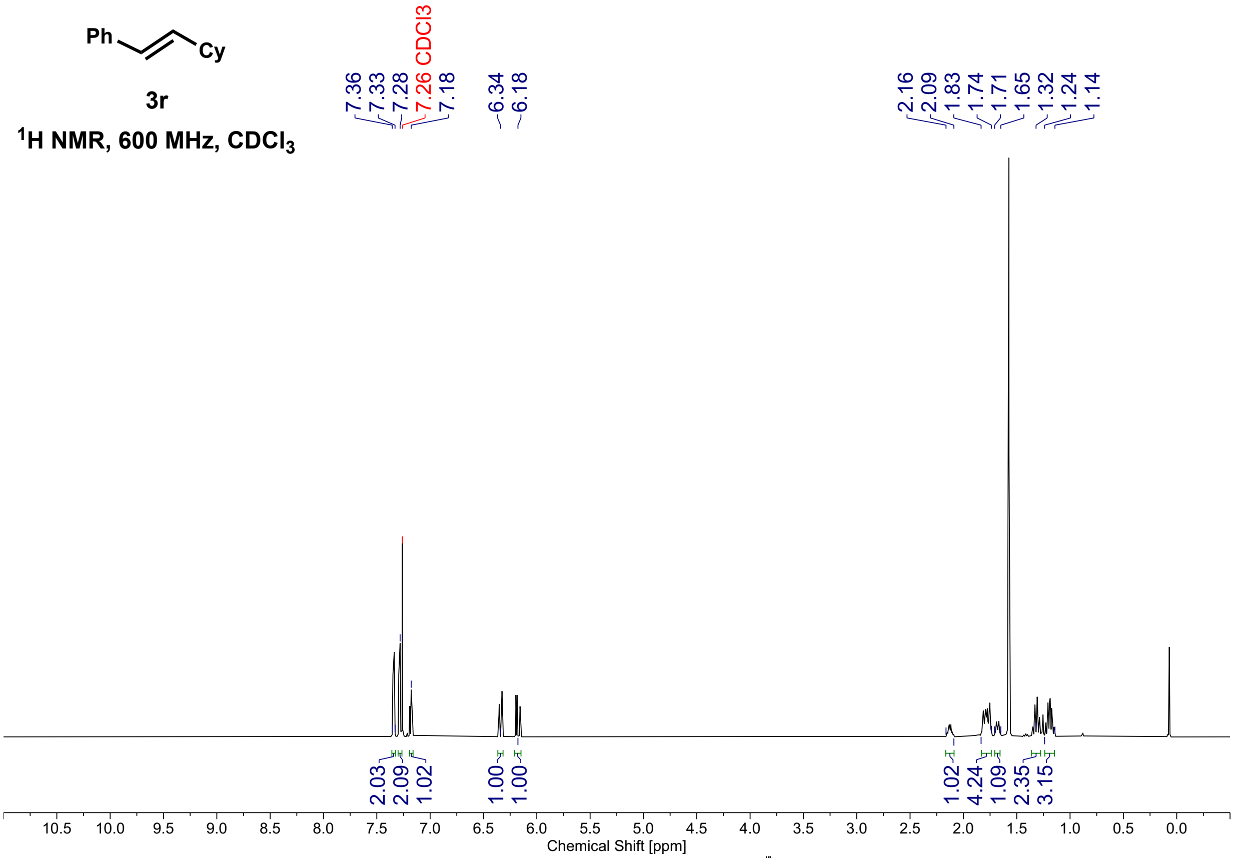

3r

^{1}H NMR, 600 MHz, $CDCl_3$

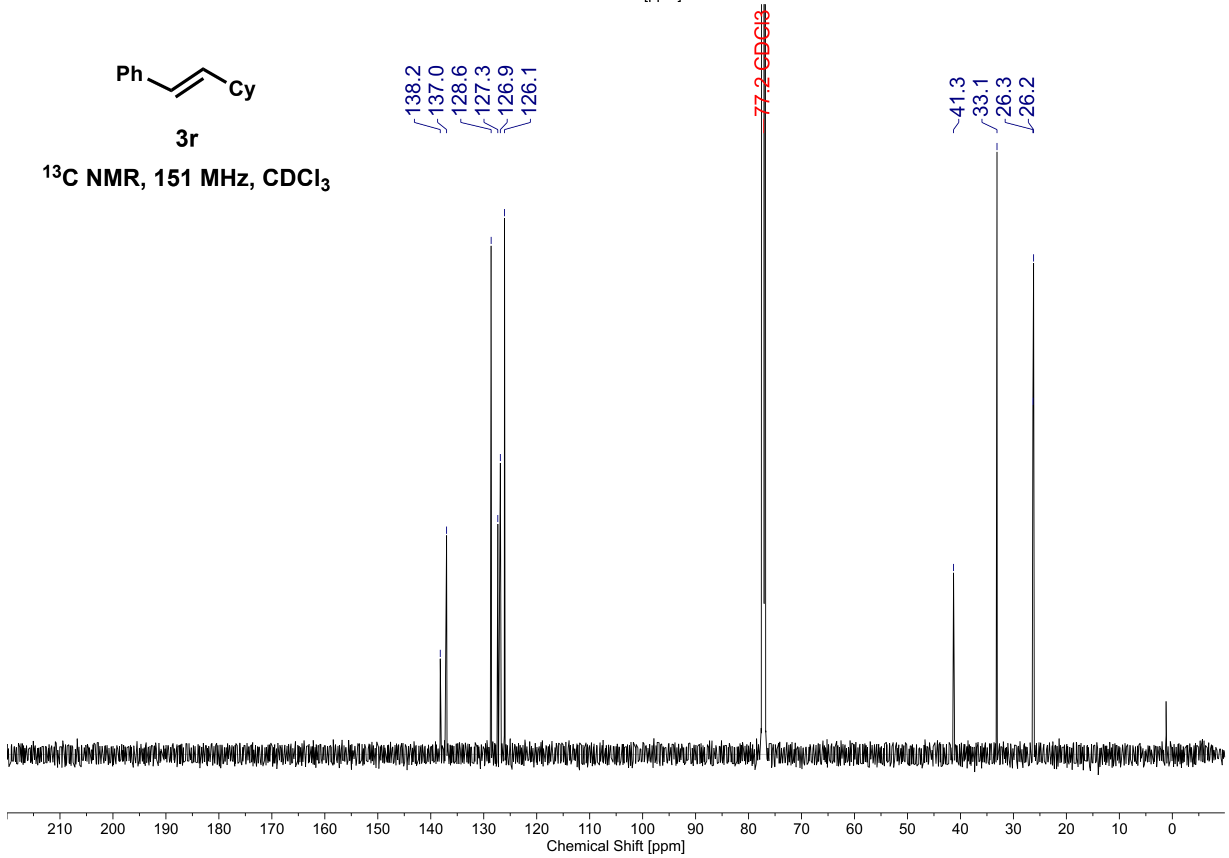

3r

^{13}C NMR, 151 MHz, $CDCl_3$

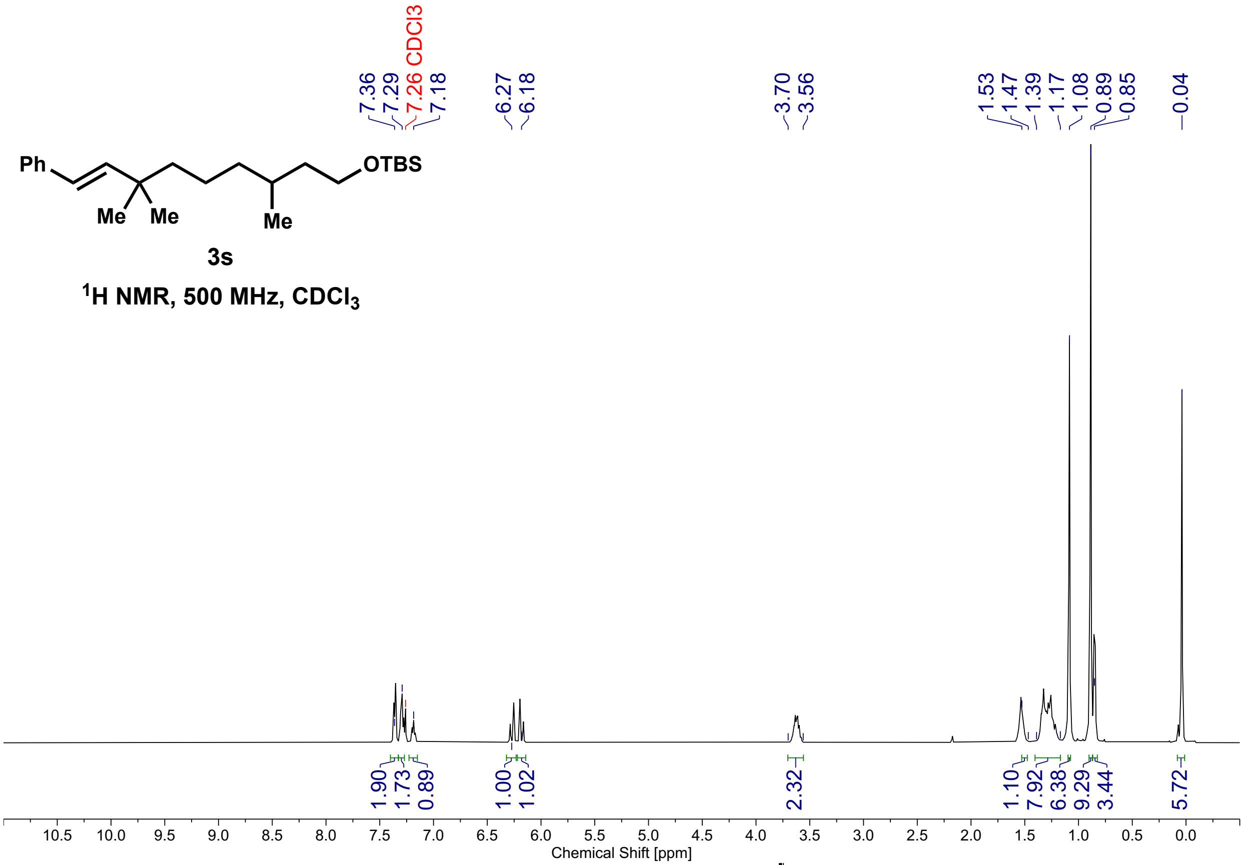
7.36
7.29
7.26 CDCl3
7.18
6.27
6.18
3.70
3.56
1.53
1.47
1.39
1.17
1.08
0.89
0.85
-0.04
Ph
OTBS
Me
Me
Me
3s
1H NMR, 500 MHz, CDCl3
1.90
1.73
0.89
1.00
1.02
2.32
1.10
7.92
6.38
9.29
3.44
5.72
Chemical Shift [ppm]

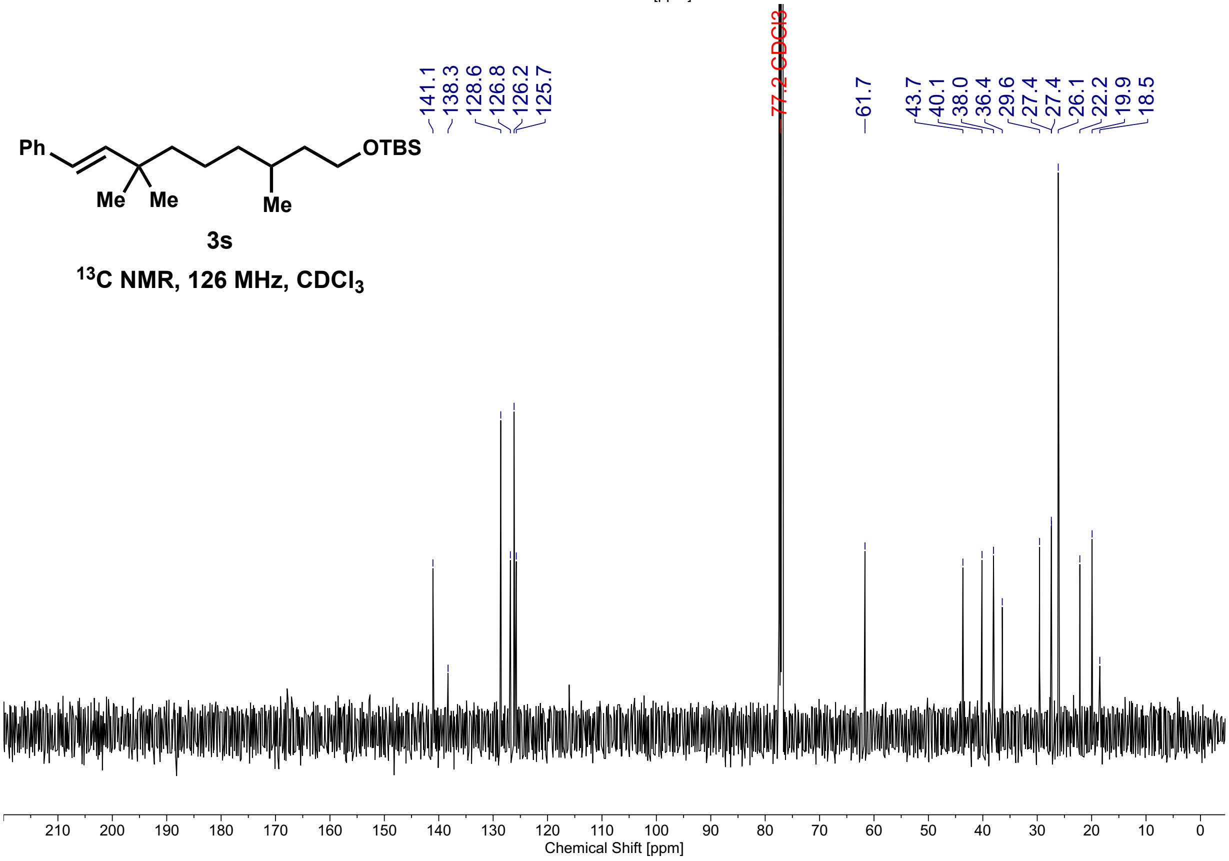
141.1
138.3
128.6
126.8
126.2
125.7
77.2 CDCl3
61.7
43.7
40.1
38.0
36.4
29.6
27.4
27.4
26.1
22.2
19.9
18.5
Ph
OTBS
Me
Me
Me
3s
13C NMR, 126 MHz, CDCl3
Chemical Shift [ppm]

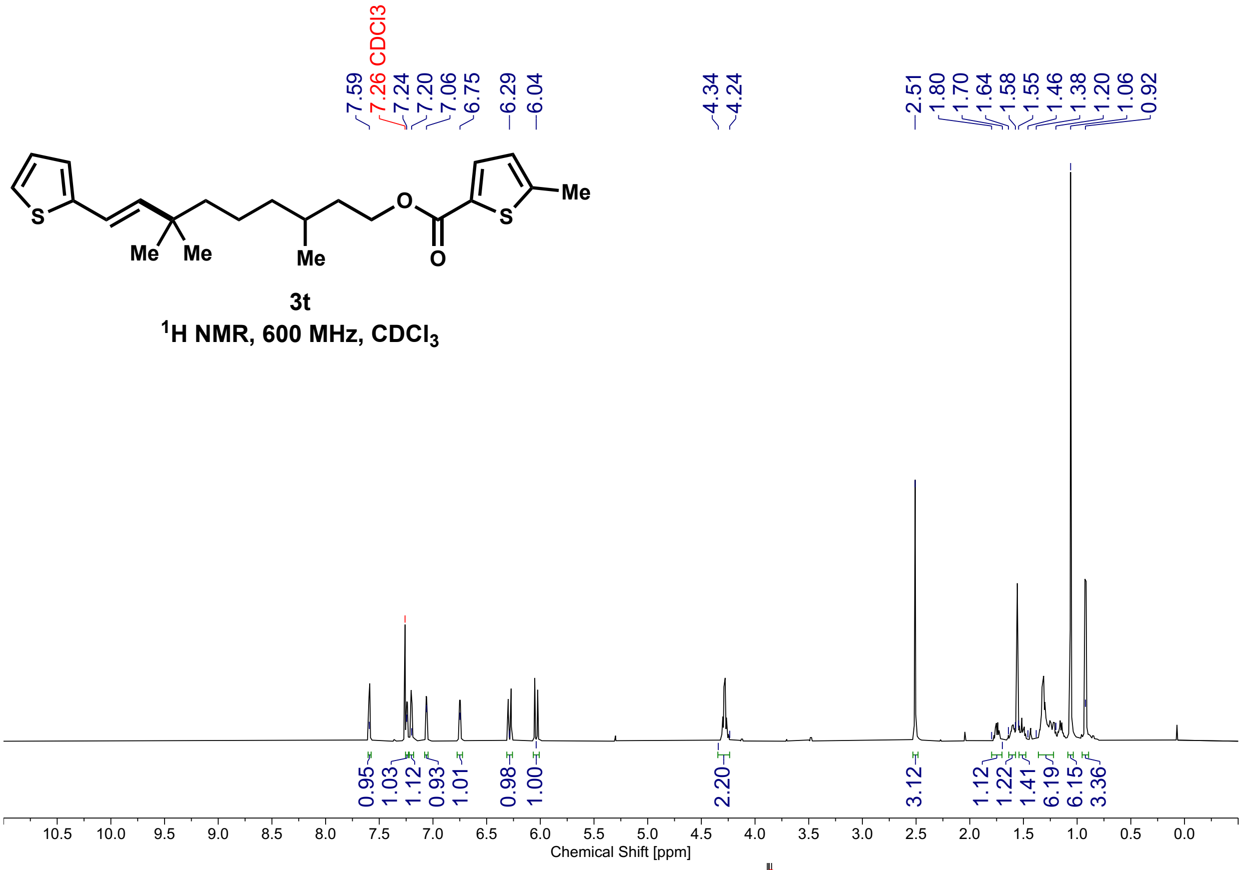

3t

^{1}H NMR, 600 MHz, $CDCl_3$

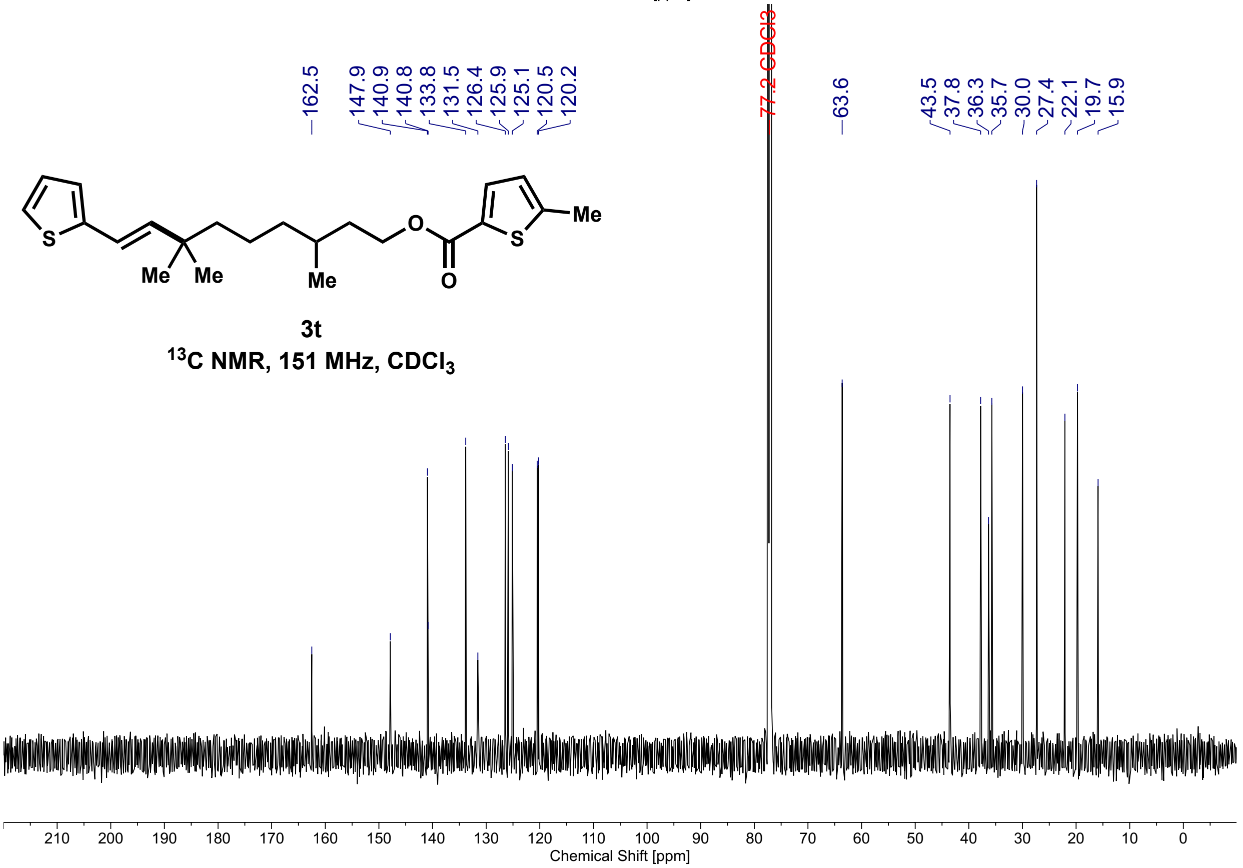

3t

^{13}C NMR, 151 MHz, $CDCl_3$

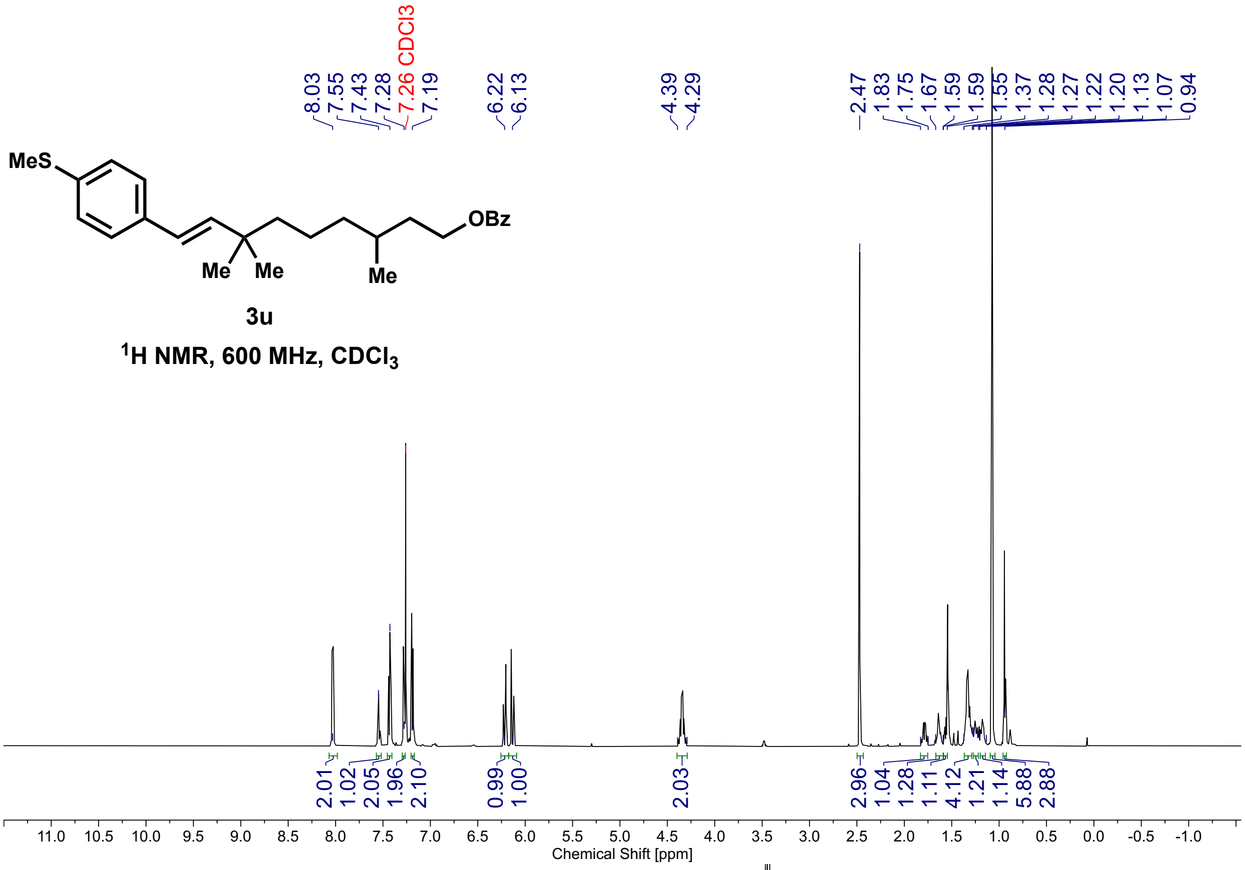
MeS
OBz
Me Me
Me
3u
1H NMR, 600 MHz, CDCl3
8.03
7.55
7.43
7.28
7.26 CDCl3
7.19
6.22
6.13
4.39
4.29
2.47
1.83
1.75
1.67
1.59
1.59
1.55
1.37
1.28
1.27
1.22
1.20
1.13
1.07
0.94
2.01
1.02
2.05
1.96
2.10
0.99
1.00
2.03
2.96
1.04
1.28
1.11
4.12
1.21
1.14
5.88
2.88
11.0 10.5 10.0 9.5 9.0 8.5 8.0 7.5 7.0 6.5 6.0 5.5 5.0 4.5 4.0 3.5 3.0 2.5 2.0 1.5 1.0 0.5 0.0 -0.5 -1.0
Chemical Shift [ppm]

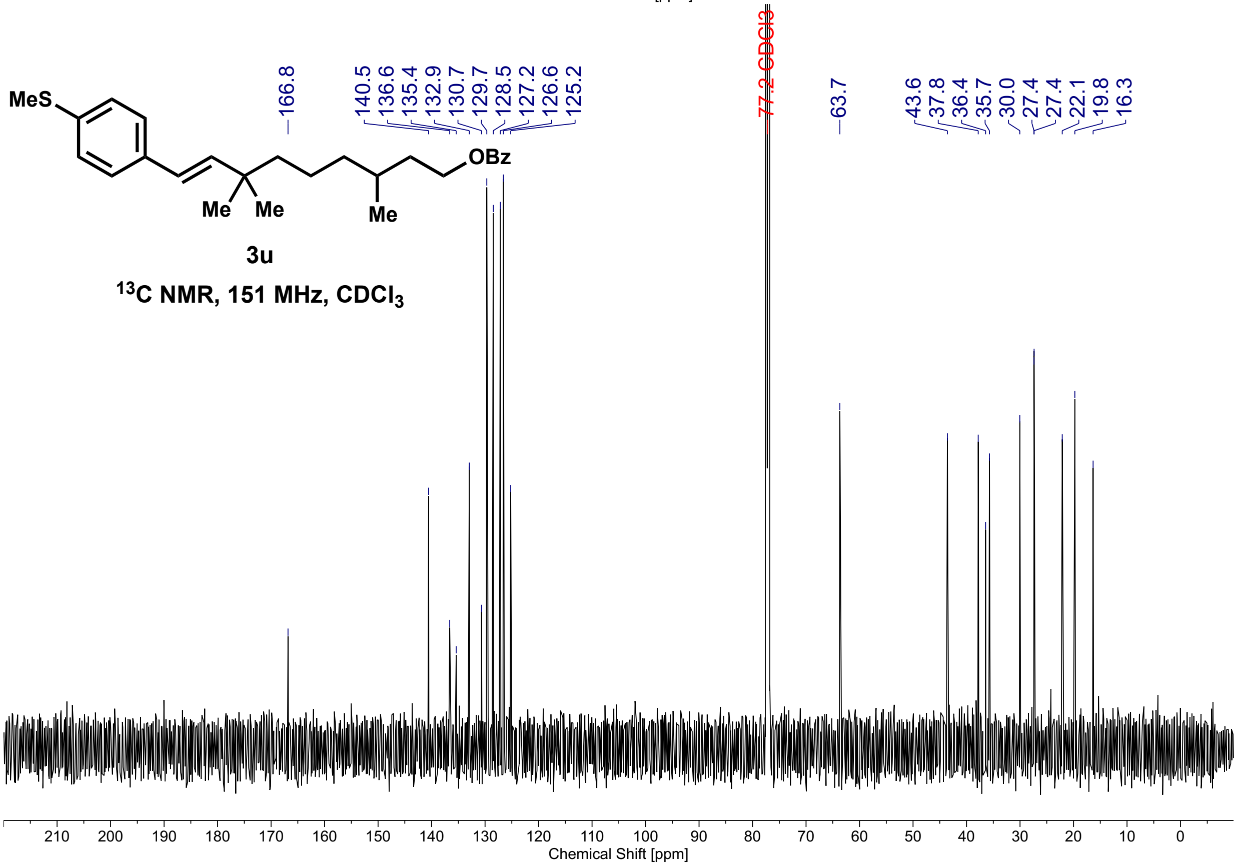
MeS
OBz
Me Me
Me
3u
13C NMR, 151 MHz, CDCl3
166.8
140.5
136.6
135.4
132.9
130.7
129.7
128.5
127.2
126.6
125.2
77.2 CDCl3
63.7
43.6
37.8
36.4
35.7
30.0
27.4
27.4
22.1
19.8
16.3
210 200 190 180 170 160 150 140 130 120 110 100 90 80 70 60 50 40 30 20 10 0
Chemical Shift [ppm]

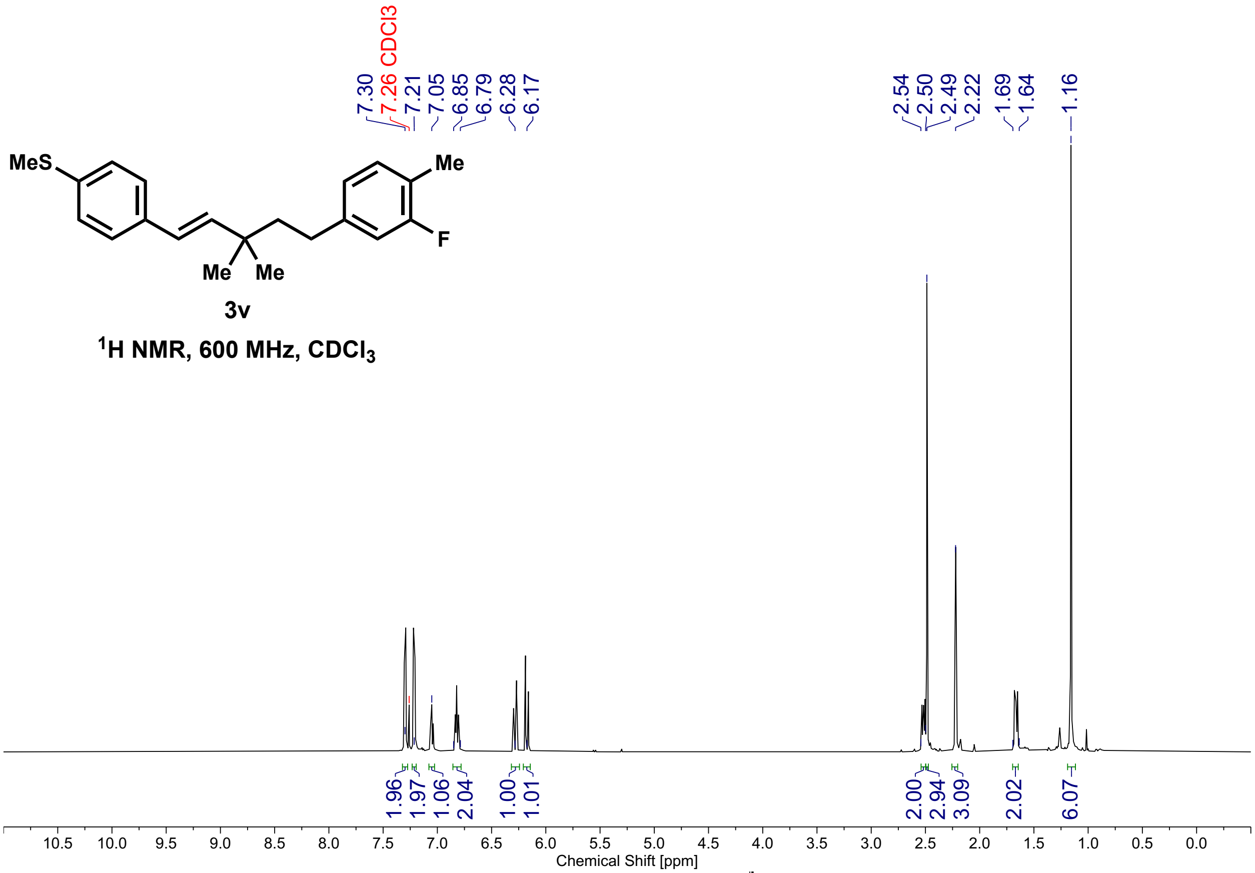
7.30
7.26 CDCl3
7.21
7.05
6.85
6.79
6.28
6.17
2.54
2.50
2.49
2.22
1.69
1.64
1.16
MeS
Me
F
Me
Me
3v
^{1}H NMR, 600 MHz, $CDCl_3$
1.96
1.97
1.06
2.04
1.00
1.01
2.00
2.94
3.09
2.02
6.07
Chemical Shift [ppm]

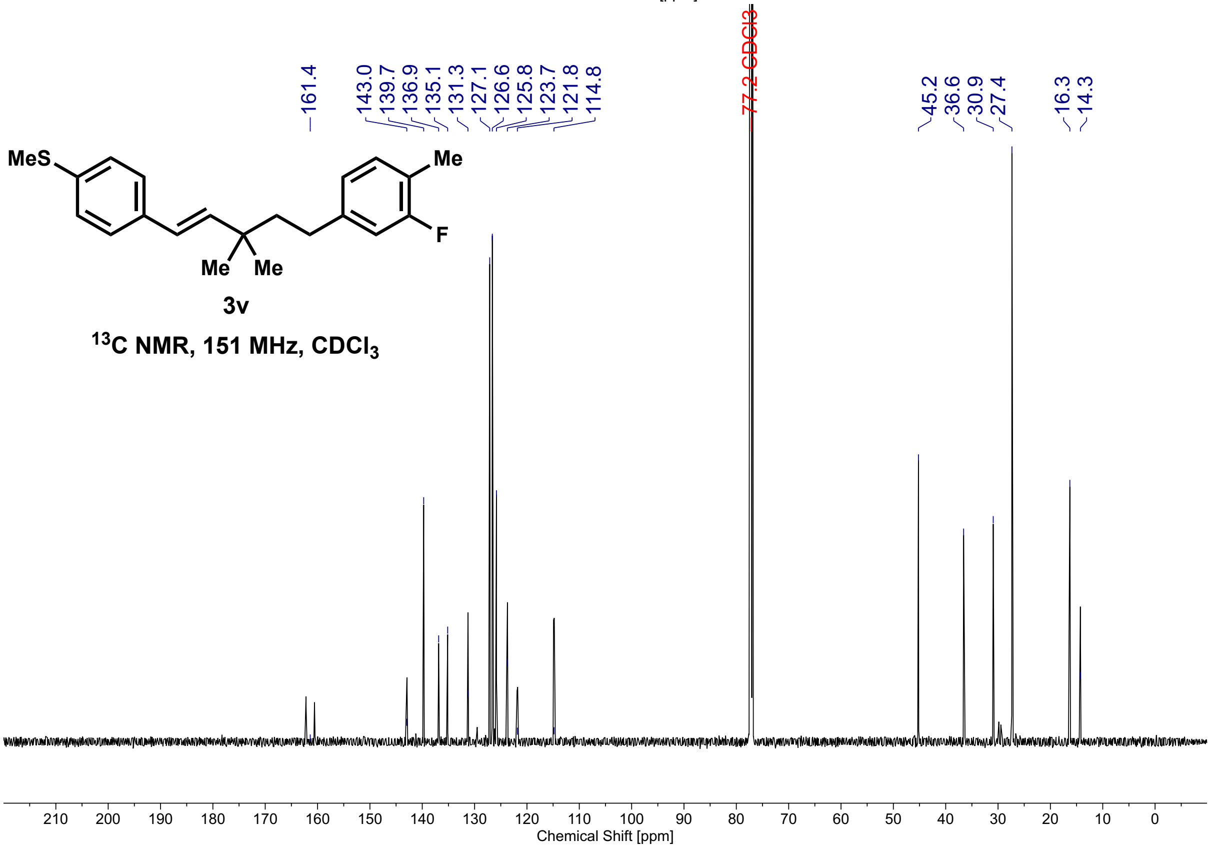
161.4
143.0
139.7
136.9
135.1
131.3
127.1
126.6
125.8
123.7
121.8
114.8
77.2 CDCl3
45.2
36.6
30.9
27.4
16.3
14.3
MeS
Me
F
Me
Me
3v
^{13}C NMR, 151 MHz, $CDCl_3$
Chemical Shift [ppm]

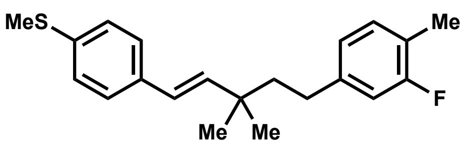

3v

^{19}F NMR, 471 MHz, $CDCl_3$

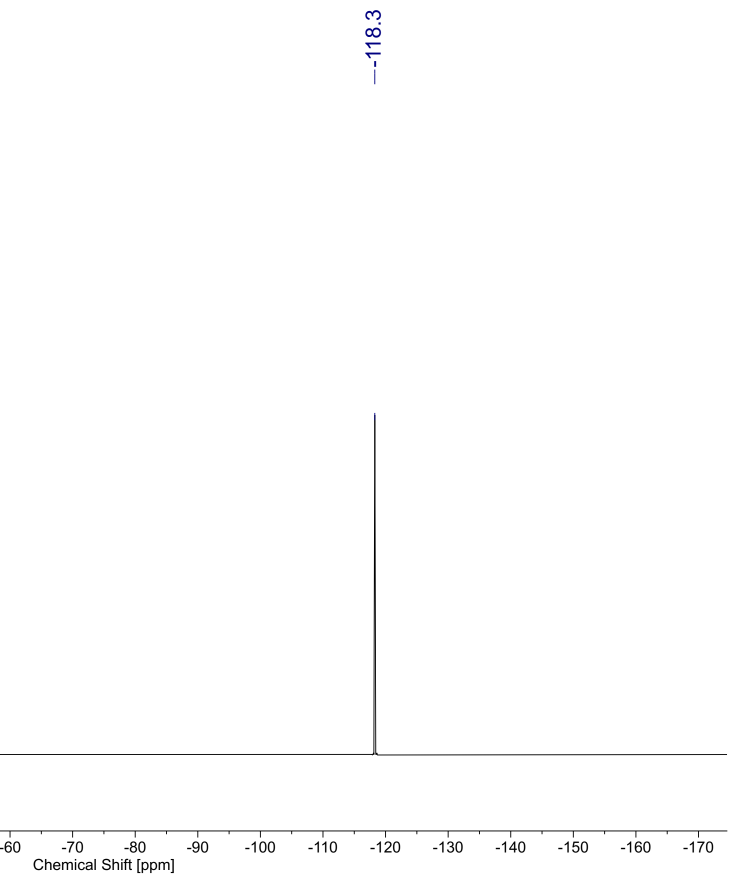

9. References

1) Yuan, P.; Yang, Z.; Zhang, S.; Zhu, C.; Yang, X.; Meng, Q. Deconstructive Carboxylation of Activated Alkenes with Carbon Dioxide. *Angew. Chem. Int. Ed.* **2024**, *63* (5), e202313030.
2) Golfmann, M.; Glagow, L.; Giakoumidakis, A.; Golz, C.; Walker, J. C. L. Organophotocatalytic [2+2] Cycloaddition of Electron-Deficient Styrenes. *Chem. Eur. J.* **2023**, *29* (3), e202202373.
3) Schuppe, A. W.; Knippel, J. L.; Borrajo-Calleja, G. M.; Buchwald, S. L. Enantioselective Hydroalkenylation of Olefins with Enol Sulfonates Enabled by Dual Copper Hydride and Palladium Catalysis. *J. Am. Chem. Soc.* **2021**, *143* (14), 5330–5335.
4) Rakesh; Sun, D.; Lee, R. B.; Tangallapally, R. P.; Lee, R. E. Synthesis, Optimization and Structure–Activity Relationships of 3,5-Disubstituted Isoxazolines as New Anti-Tuberculosis Agents. *European Journal of Medicinal Chemistry* **2009**, *44* (2), 460–472.
5) Seo, H.; Liu, A.; Jamison, T. F. Direct β-Selective Hydrocarboxylation of Styrenes with CO2 Enabled by Continuous Flow Photoredox Catalysis. *J. Am. Chem. Soc.* **2017**, *139* (40), 13969–13972.
6) Wienhöfer, G.; Westerhaus, F. A.; Jagadeesh, R. V.; Junge, K.; Junge, H.; Beller, M. Selective Iron-Catalyzed Transfer Hydrogenation of Terminal Alkynes. *Chem. Commun.* **2012**, *48* (40), 4827–4829.
7) Gribble, M. W.; Pirnot, M. T.; Bandar, J. S.; Liu, R. Y.; Buchwald, S. L. Asymmetric Copper Hydride-Catalyzed Markovnikov Hydrosilylation of Vinylarenes and Vinyl Heterocycles. *J. Am. Chem. Soc.* **2017**, *139* (6), 2192–2195.
8) Aslam, S. N.; Stevenson, P. C.; Phythian, S. J.; Veitch, N. C.; Hall, D. R. Synthesis of Cicerfuran, an Antifungal Benzofuran, and Some Related Analogues. *Tetrahedron* **2006**, *62* (17), 4214–4226.
9) Jiao, Z.; Jaunich, K. T.; Tao, T.; Gottschall, O.; Hughes, M. M.; Turlik, A.; Schuppe, A. W. Unified Approach to Deamination and Deoxygenation Through Isonitrile Hydrodecyanation: A Combined Experimental and Computational Investigation. *Angew. Chem. Int. Ed*. **2024**, *63* (25), e202405779.
10) Pérez-Sánchez, C.; Rigotti, T.; Tortosa, M. Visible-Light-Mediated Deaminative Alkylation of Primary Amines with Silacarboxylic Acids via Isonitrile Formation. *Org. Lett.* **2025**, *27* (2), 583–587.
11) Wang, G.-Z.; Shang, R.; Fu, Y. Irradiation-Induced Palladium-Catalyzed Decarboxylative Heck Reaction of Aliphatic *N* -(Acyloxy)Phthalimides at Room Temperature. *Org. Lett.* **2018**, *20* (3), 888–891.
12) Jang, Y.-J.; Yan, M.-C.; Lin, Y.-F.; Yao, C.-F. A Simple Radical Addition−Elimination Route to Geometrically Pure (E)-Alkene and Chromanone Derivatives via β-Nitrostyrene. *J. Org. Chem.* **2004**, *69* (11), 3961–3963.
13) Zhao, H.; Zong, Y.; Sun, Y.; An, G.; Wang, J. An Organocatalytic System for Z-Alkene Synthesis via a Hydrogen-Bonding-Assisted Photoinduced Electron Donor–Acceptor Complex. *Org. Lett.* **2024**, *26* (8), 1739–1744.
14) Cao, H.; Jiang, H.; Feng, H.; Kwan, J. M. C.; Liu, X.; Wu, J. Photo-Induced Decarboxylative Heck-Type Coupling of Unactivated Aliphatic Acids and Terminal

Alkenes in the Absence of Sacrificial Hydrogen Acceptors. *J. Am. Chem. Soc.* **2018**, *140* (47), 16360–16367.

15) Zhou, Y.-B.; Wang, Y.-Q.; Ning, L.-C.; Ding, Z.-C.; Wang, W.-L.; Ding, C.-K.; Li, R.-H.; Chen, J.-J.; Lu, X.; Ding, Y.-J.; Zhan, Z.-P. Conjugated Microporous Polymer as Heterogeneous Ligand for Highly Selective Oxidative Heck Reaction. *J. Am. Chem. Soc.* **2017**, *139* (11), 3966–3969.
16) Toriyama, F.; Cornella, J.; Wimmer, L.; Chen, T.-G.; Dixon, D. D.; Creech, G.; Baran, P. S. Redox-Active Esters in Fe-Catalyzed C–C Coupling. *J. Am. Chem. Soc.* **2016**, *138* (35), 11132–11135.
17) Soltani, Y.; Dasgupta, A.; Gazis, T. A.; Ould, D. M. C.; Richards, E.; Slater, B.; Stefkova, K.; Vladimirov, V. Y.; Wilkins, L. C.; Willcox, D.; Melen, R. L. Radical Reactivity of Frustrated Lewis Pairs with Diaryl Esters. *CR-PHYS-SC* **2020**, *1* (2).
18) Yurino, T.; Tani, R.; Ohkuma, T. Pd-Catalyzed Allylic Isocyanation: Nucleophilic *N*-Terminus Substitution of Ambident Cyanide. *ACS Catal*. **2019**, *9* (5), 4434–4440.
